# Supplementary material for: Design, Synthesis, In Vitro Antifungal Activity and Mechanism Study of the Novel 4-Substituted Mandelic Acid Derivatives
Source: Int J Mol Sci. 2023 May 17;24(10):8898. doi: 10.3390/ijms24108898 (PMC10219514; doi:10.3390/ijms24108898)
Supplement: Supplementary file 1 [file ijms-24-08898-s001.zip › ijms-2373867-supplementary.pdf]

Supplementary Materials

Design, Synthesis, In Vitro Antifungal Activity and Mechanism Study of the Novel 4-Substituted Mandelic Acid Derivatives

Biao Chen [1], Dandan Song [2], Huabin Shi [2], Kuai Chen [2], Zhibing Wu [2,*] and Huifang Chai [1,*]

[1] School of Pharmacy, Guizhou University of Traditional Chinese Medicine, Guiyang 550025, China; gzdxchenbiao@163.com

[2] State Key Laboratory Breeding Base of Green Pesticide and Agricultural Bioengineering, Key Laboratory of Green Pesticide and Agricultural Bioengineering, Ministry of Education, Center for R&D of Fine Chemicals of Guizhou University, Guiyang 550025, China; 15284637047@163.com (D.S.); shb1126@163.com (H.S.); m15185174268@163.com (K.C.)

* Correspondence: zbwu@gzu.edu.cn (Z.W.); hfchai@gzy.edu.cn (H.C.)

1. ^{1}H, ^{13}C NMR and HRMS data of the title compounds…………..………..………..……… 2-11

2. Copies of ^{1}H, ^{13}C, ^{19}F NMR, HRMS, and HPLC of the title compounds………….………12-76

3. Crystallographic data of compound **E$_9$** ……………..………..………..………..……….......77

4. The regression equation of the title compounds with EC$_{50}$ values ………………………..…78-82

1. ^{1}H, ^{13}C NMR, and HRMS data of the title compounds

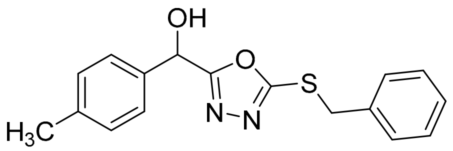

data for **E_1**: A yellow solid, yield 56%, purity 99.5%, m.p. 95-96 °C. ^{1}H NMR (400 MHz, DMSO-*d_6*) δ 7.35-7.26 (m, 7 H, benzene H), 7.19 (d, *J* = 8.0 Hz, 2H benzene H), 6.69 (d, *J* = 8.0 Hz, 1H, OH), 5.95 (d, *J* = 4.0 Hz, 1H, CH), 4.44 (s, 2H, CH_2), 2.29 (s, 3H, CH_3); ^{13}C NMR (101 MHz, DMSO-*d_6*) δ 169.1, 163.9, 138.0, 136.9, 136.7, 129.5, 129.4, 129.0, 128.2, 126.8, 66.7, 36.2, 21.2; HRMS: calcd for $C_{17}H_{16}N_2O_2S$, $[M+H]^+$ 313.1005, found 313.1002.

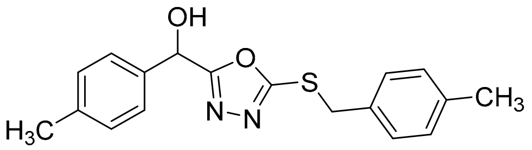

Data for **E_2**: A yellow solid, yield 55%, purity 95.4%, m.p. 135-136 °C. ^{1}H NMR (400 MHz, DMSO-*d_6*) δ 7.32 (d, *J* = 12.0 Hz, 2H, benzene H), 7.20 (t, *J* = 8.0 Hz, 4H, benzene H), 7.07 (d, *J* = 8.0 Hz, 2H, benzene H), 6.66 (d, *J* = 4.0 Hz, 1H, OH), 5.94 (d, *J* = 8.0 Hz, 1H, CH), 4.40 (s, 2H, CH_2), 2.30 (s, 3H, CH_3), 2.25 (s, 3H, CH_3); ^{13}C NMR (101 MHz, DMSO-*d_6*) δ 169.1, 163.9, 137.9, 137.5, 136.7, 133.8, 129.6, 129.5, 129.3, 126.8, 66.8, 36.1, 21.2; HRMS: calcd for $C_{18}H_{18}N_2O_2S$, $[M+H]^+$ 327.1162, found 327.1152.

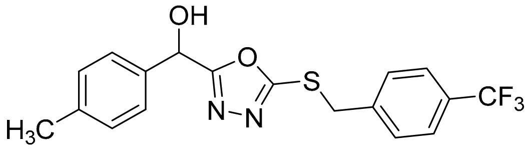

data for **E_3**: A white solid, yield 38%, purity 99.7%, m.p. 117-118 °C. ^{1}H NMR (400 MHz, DMSO-*d_6*) δ 7.59 (dd, *J* = 20.0, 8.0 Hz, 4 H, benzene H), 7.3 (d, *J* = 8.0 Hz, 2 H, benzene H), 7.18 (d, *J* = 8.0 Hz, 2H, benzene H), 6.65 (d, *J* = 8.0 Hz, 1H, CH), 5.92 (d, *J* = 8.0 Hz, 1H, OH), 4.53 (s, 2H, CH_2), 2.29 (s, 3H, CH_3); ^{13}C NMR (101 MHz, DMSO-*d_6*) δ 169.2, 163.6, 142.2, 137.9, 136.6, 130.2, 129.5, 128.6 (q, *J* = 31.7 Hz), 126.8, 125.8 (q, *J* = 3.8 Hz), 66.6, 35.4, 21.2; ^{19}F NMR (376 MHz, DMSO-*d_6*) δ -61.05; HRMS: calcd for $C_{18}H_{15}F_3N_2O_2S$, $[M+H]^+$ 381.0879, found 381.0868.

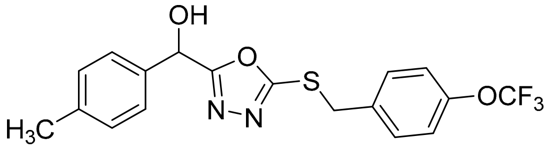

data for **E_4**: A yellow solid, yield 56%, purity 93.5%, m.p.140-141 °C. ^{1}H NMR (400 MHz, DMSO-*d_6*) δ 7.48 (dd, *J* = 8.0, 4.0 Hz, 2H, benzene H), 7.31 (d, *J* = 8.0 Hz, 2H, benzene H), 7.25 (d, *J* = 8.0 Hz, 2H, benzene H), 7.19 (d, *J* = 8.0 Hz, 2H, benzene H), 6.65 (d, *J* = 8.0 Hz, 1H, OH), 5.93 (d, *J* = 4.0 Hz, 1H, CH), 4.48 (s, 2H, CH_2), 2.29 (s, 3H, CH_3); ^{13}C NMR (101 MHz, DMSO-*d_6*) δ 169.2, 163.7, 148.2,137.9, 136.7 (d, *J* = 5.0 Hz), 131.4, 129.5, 126.8, 121.5, 66.6, 35.2, 21.2; ^{19}F NMR (377 MHz, DMSO-*d_6*) δ -56.8; HRMS: calcd for $C_{18}H_{16}F_3N_2O_3S$, $[M+H]^+$ 397.0828, found 397.0818.

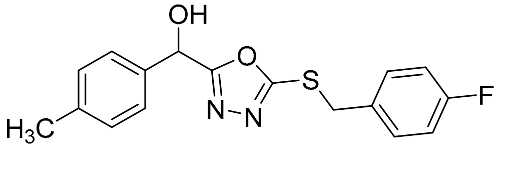

data for **E_5**: A white solid, yield 58%, purity 96.1%, m.p. 132-133 °C. ^{1}H NMR (400 MHz, DMSO-*d_6*) δ 7.41-7.36 (m, 2H, benzene H), 7.30 (d, *J* = 8.0 Hz, 2H, benzene H), 7.19 (d, *J* = 8.0 Hz, 2H, benzene H), 7.12-7.06 (m, 2H, benzene H), 6.65 (d, *J* = 4.0 Hz, 1H, OH), 5.92 (d, *J* = 8.0 Hz, 1H, CH), 4.44 (s, 2H, CH_2), 2.30 (s, 3H, CH_3); ^{13}C NMR (101 MHz, DMSO-*d_6*) δ 169.1, 163.8, 162.0 (d, *J* = 244.1 Hz), 138.0, 136.7, 133.3 (d, *J* = 3.0 Hz), 131.5 (d, *J* = 8.2 Hz), 129.5, 126.8, 115.8 (d, *J* = 21.4 Hz), 66.6, 35.4, 21.2; ^{19}F NMR (377 MHz, DMSO-*d_6*) δ -114.4; HRMS: calcd for $C_{17}H_{15}FN_2O_2S$, $[M+H]^+$ 331.0911, found 331.0902.

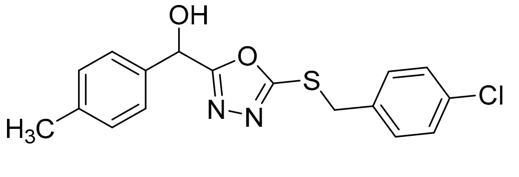

data for **E_6**: A white solid, yield 39%, purity 96.8%, m.p. 114-115 °C. ^{1}H NMR (400 MHz, DMSO-*d_6*) δ 7.40-7.29 (m, 6 H, benzene H), 7.19 (d, *J* = 8.0 Hz, 2 H, benzene H), 6.66 (d, *J* = 8.0 Hz, 1H, OH), 5.92 (d, *J* = 8.0 Hz, 1H, CH), 4.44 (s, 2H, CH_2), 2.30 (s, 3H, CH_3); ^{13}C NMR (101 MHz, DMSO-*d_6*) δ 169.2, 163.7, 138.0, 136.6, 136.3, 132.8, 131.3, 129.5, 128.9, 126.8, 66.6, 35.4, 21.2; HRMS: calcd for $C_{17}H_{15}ClN_2O_2S$, $[M+H]^+$ 347.0616, found 347.0606.

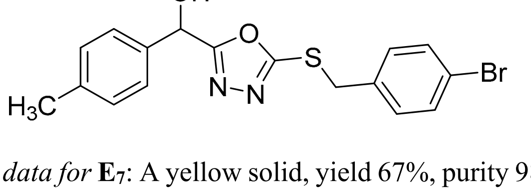

data for **E_7**: A yellow solid, yield 67%, purity 98.9%, m.p. 143-144 °C. ^{1}H NMR (400 MHz, DMSO-*d_6*) δ 7.37-7.29 (m, 6 H, benzene H), 7.19 (d, *J* = 8.0 Hz, 2 H, benzene H), 6.66 (d, *J* = 8.0 Hz, 1H, OH), 5.92 (d, *J* = 4.0 Hz, 1H, CH), 4.44 (s, 2H, CH_2), 2.30 (s, 3H, CH_3); ^{13}C NMR (101 MHz,

DMSO-d_6) δ 169.2, 163.7, 138.0, 136.6, 136.2, 132.8, 131.3, 129.5, 128.9, 126.8, 66.6, 35.4, 21.2; HRMS: calcd for $C_{17}H_{15}BrN_2O_2S$, $[M+H]^+$ 391.0110, found 391.0105.

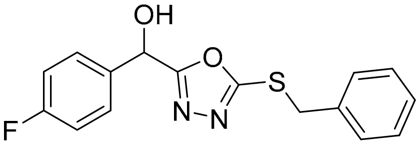

data for **E8**: A yellow solid, yield 77%, purity 99.4%, m.p. 92-93 °C. ^{1}H NMR (400 MHz, DMSO-d_6) δ 7.29-7.24 (m, 5 H, benzene H), 7.10-7.06 (m, 2H, benzene H), 7.00-6.95 (m, 2H, benzene H), 5.96 (d, J = 4.0 Hz, 1H, OH), 4.74 (d, J = 4.0 Hz, 1H, CH), 3.96 (s, 2H, CH_2); ^{13}C NMR (101 MHz, DMSO-d_6) δ 170.3, 163.0, 160.6, 138.3, 137.8 (d, J = 2.9 Hz), 129.1, 129.0 (d, J = 8.2 Hz), 128.5, 127.4, 114.9 (d, J = 21.3 Hz), 72.5, 59.9; ^{19}F NMR (377 MHz, DMSO-d_6) δ -115.6; HRMS: calcd for $C_{16}H_{13}FN_2O_2S$, $[M+H]^+$ 317.0755, found 317.0753.

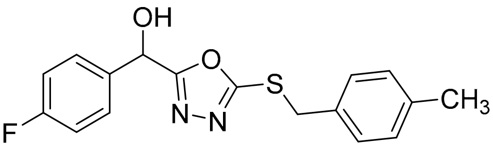

data for **E9**: A white solid, yield 78%, purity 99.5%, m.p. 169-170 °C. ^{1}H NMR (400 MHz, DMSO-d_6) δ 7.51-7.46 (m, 2H, benzene H), 7.25-7.19 (m, 4H, benzene H),7.08 (d, J = 8.0 Hz, 2H, benzene H), 6.78 (d, J = 4.0 Hz, 1H, OH), 6.00 (d, J = 4.0 Hz, 1H, CH), 4.41 (s, 2H, CH_2), 2.25 (s, 3H, CH_3); ^{13}C NMR (101 MHz, DMSO-d_6) δ 168.8, 164.1, 163.6, 159.3, 135.9 (d, J = 2.9 Hz), 130.7, 130.3, 129.1 (d, J = 8.4 Hz), 115.8 (d, J = 21.5 Hz), 114.4, 66.1, 55.5, 35.9; ^{19}F NMR (377 MHz, DMSO-d_6) δ -114.1; HRMS: calcd for $C_{17}H_{15}FN_2O_2S$, $[M+H]^+$ 331.0911, found 331.0902.

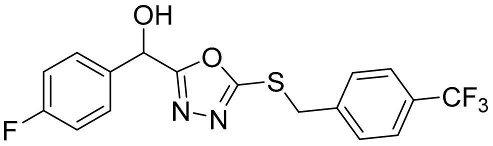

data for **E10**: A yellow solid, yield 88%, purity 96.9%, m.p. 74-75 °C. ^{1}H NMR (400 MHz, DMSO-d_6) δ 7.62 (dd, J = 20.0, 8.0 Hz, 4 H, benzene H), 7.49-7.45 (m, 2 H, benzene H), 7.24-7.18 (d, 2H, benzene H), 6.79 (d, J = 8.0 Hz, 1H, OH), 5.99 (d, J = 4.0 Hz, 1H, CH), 4.54 (s, 2H, CH_2), 2.29 (s, 3H, CH_3); ^{13}C NMR (101 MHz, DMSO-d_6) δ 169.0 (d, J = 0.5 Hz), 163.8, 162.4 (d, J = 244.1 Hz), 161.1, 142.2, 135.8 (d, J = 3.0 Hz), 130.2, 129.1 (d, J = 8.4 Hz), 125.8 (q, J = 3.8 Hz), 124.6 (q, J = 272.1 Hz), 115.7 (d, J = 21.6 Hz), 66.0, 35.4; ^{19}F NMR (376 MHz, DMSO-d_6) δ -61.0, -114.11; HRMS: calcd for $C_{17}H_{12}F_4N_2O_2S$, $[M+H]^+$ 385.0628, found 385.0619.

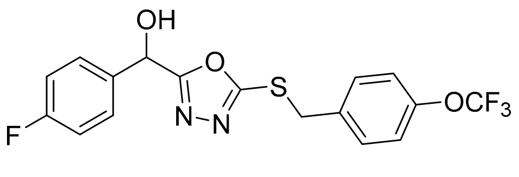

data for **E_{11}**: A white solid, yield 28%, purity 99.3%, m.p. 66-67 ºC. ^{1}H NMR (400 MHz, DMSO-*d_6*) δ 7.42-7.38 (m, 4H, benzene H), 7.17-7.13 (m, 4H, benzene H), 6.77 (d, *J* = 8.0 Hz, 1H, OH), 6.00 (d, *J* = 4.0 Hz, 1H, CH), 4.49 (s, 2H, CH_2); ^{13}C NMR (101 MHz, DMSO-*d_6*) δ 163.9, 160.9, 136.7, 131.4, 131.0, 129.2 (d, *J* = 8.2 Hz), 129.0, 121.5, 115.7 (d, *J* = 21.6 Hz), 115.2 (d, *J* = 21.3 Hz), 66.1, 35.2; ^{19}F NMR (377 MHz, DMSO-*d_6*) δ -56.8, -114.1; HRMS: calcd for $C_{17}H_{12}F_4N_2O_3S$, $[M+H]^+$ 401.0578, found 401.0570.

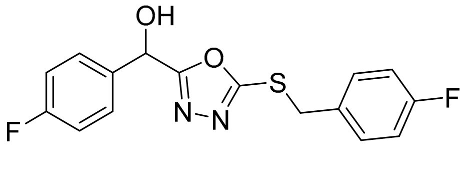

data for **E_{12}**: A white solid, yield 71%, purity 98.8%, m.p. 63-64 ºC. ^{1}H NMR (400 MHz, DMSO-*d_6*) δ 7.50-7.46 (m, 2H, benzene H), 7.40-7.33 (m, 2H, benzene H), 7.24-7.19 (m, 3H, benzene H), 7.12-7.08 (m, 1H, benzene H), 6.79 (d, *J* = 8.0 Hz, 1H, OH), 6.00 (d, *J* = 4.0 Hz, 1H, CH), 4.48 (s, 2H, CH_2); ^{13}C NMR (101 MHz, DMSO-*d_6*) δ 169.1, 163.6, 135.8 (d, *J* = 3.0 Hz), 131.8 (d, *J* = 3.4 Hz), 130.7 (d, *J* = 8.2 Hz), 129.1 (d, *J* = 8.3 Hz), 125.0 (d, *J* = 3.8 Hz), 123.9, 115.9 (d, *J* = 2.8 Hz), 115.6, 66.1, 30.2 (d, *J* = 3.3 Hz); ^{19}F NMR (377 MHz, DMSO-*d_6*) δ -115.7, -116.0; HRMS: calcd for $C_{16}H_{12}F_2N_2O_2S$, $[M+H]^+$ 335.0660, found 335.0655.

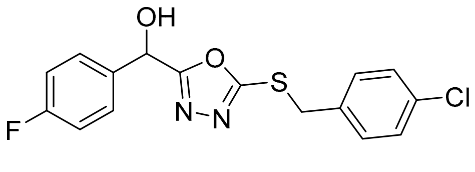

data for **E_{13}**: A white solid, yield 38%, purity 96.6%, m.p. 82-83 ºC. ^{1}H NMR (400 MHz, DMSO-*d_6*) δ 7.50-7.46 (m, 2H, benzene H), 7.39-7.32 (m, 4H, benzene H), 7.25-7.19 (m, 2H, benzene H), 6.78 (d, *J* = 4.0 Hz, 1H, OH), 6.00 (d, *J* = 4.0 Hz, 1H, CH), 4.45 (s, 2H, CH_2); ^{13}C NMR (101 MHz, DMSO-*d_6*) δ 168.9, 163.9, 162.4 (d, *J* = 244.2 Hz), 136.2, 135.8, 132.8, 131.3, 129.1 (d, *J* = 8.4 Hz), 128.9, 115.7 (d, *J* = 21.6 Hz), 66.1, 35.4; ^{19}F NMR (377 MHz, DMSO-*d_6*) δ -114.1; HRMS: calcd for $C_{16}H_{12}ClFN_2O_2S$, $[M+H]^+$ 351.0365, found 351.0354.

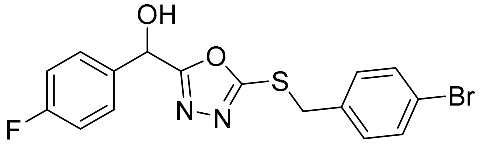

data for **E$_{14}$**: A yellow solid, yield 75%, purity 98.1%, m.p. 130-131 ºC. ^{1}H NMR (400 MHz, DMSO-*d$_6$*) δ 7.50-7.46 (dd, *J* = 12.0, 8.0 Hz, 4H, benzene H), 7.32 (d, *J* = 8.0 Hz, 2H, benzene H), 7.24-7.19 (m, 2H, benzene H), 6.78 (d, *J* = 4.0 Hz, 1H, OH), 6.00 (d, *J* = 4.0 Hz, 1H, CH), 4.43 (s, 2H, CH_2); ^{13}C NMR (101 MHz, DMSO-*d$_6$*) δ 68.9, 163.9, 162.4 (d, *J* = 244.2 Hz), 136.7, 135.8 (d, *J* = 2.9 Hz), 131.7 (d, *J* = 24.7 Hz), 131.4, 129.1 (d, *J* = 8.4 Hz), 121.4, 115.7 (d, *J* = 21.6 Hz), 66.1, 35.5; ^{19}F NMR (377 MHz, DMSO-*d$_6$*) δ -114.0; HRMS: calcd for $C_{16}H_{12}BrFN_2O_2S$, $[M+H]^+$ 394.9860, found 394.9851.

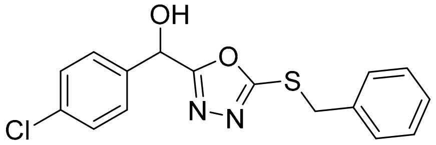

data for **E$_{15}$**: A light yellow soild, yield 13%, purity 99.8%, m.p. 89-90 ºC. ^{1}H NMR (400 MHz, DMSO-*d$_6$*) δ 7.46 (s, 4H, benzene H), 7.34-7.26 (m, 5H, benzene H), 6.86 (d, *J* = 4.0 Hz, 1H, OH), 6.00 (d, *J* = 4.0 Hz, 1H, CH), 4.45 (s, 2H, CH_2); ^{13}C NMR (125 MHz, DMSO-*d$_6$*) δ 168.7, 164.1, 138.6, 136.9, 133.3, 129.4, 129.0, 128.9, 128.8, 128.2, 66.0, 36.2; HRMS: calcd for $C_{16}H_{13}ClN_2O_2S$, $[M+H]^+$ 345.0459, found 345.0479.

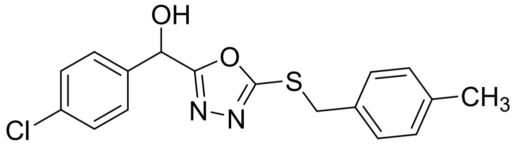

data for **E$_{16}$**: A white soild, yield 59%, purity 99.1%, m.p. 165-166 ºC. ^{1}H NMR (400 MHz, DMSO-*d$_6$*) ^{1}H NMR (400 MHz, DMSO-*d$_6$*) δ7.49 (dd, *J* = 4.0, 8.0 Hz, 2H, benzene H), 7.24-7.20 (m, 4H, benzene H), 7.07 (d, *J* = 8.0 Hz, 2H, benzene H), 6.78 (d, *J* = 4.0 Hz, 1H, OH), 6.00 (d, *J* = 8.0 Hz, 1H, CH), 4.41 (s, 2H, CH_2), 2.25 (s, 3H, CH_3); ^{13}C NMR (101 MHz, DMSO-*d$_6$*) δ 168.8, 164.0, 163.5, 161.2, 137.5, 135.9 (d, *J* = 3.0 Hz), 133.8, 129.4 (d, *J* = 22.4 Hz), 129.1 (d, *J* = 8.4 Hz), 115.7 (d, *J* = 21.6 Hz), 66.1, 36.1, 21.2; HRMS: calcd for $C_{17}H_{15}ClN_2O_2S$, $[M+H]^+$ 347.0616, found 347.0609.

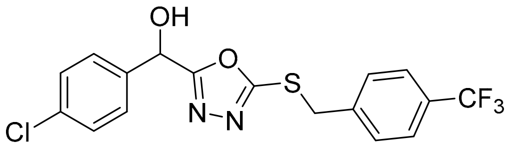

data for **E**$_{17}$: A yellow solid, yield 53%, purity 98.1%, m.p. 74-75 °C. ^{1}H NMR (400 MHz, DMSO-*d*$_6$) δ 7.63 (d, *J* = 8.0 Hz, 2 H, benzene H), 7.47-7.42 (m, 4H, benzene H), 7.13 (d, *J* = 8.0 Hz, 1H, benzene H), 7.01 (d, *J* = 8.0 Hz, 1H, benzene H), 6.85 (d, *J* = 4.0 Hz, 1H, OH), 6.01 (d, *J* = 8.0 Hz, 1H, CH), 4.54 (s, 2H, CH_2); ^{13}C NMR (101 MHz, DMSO-*d*$_6$) δ 170.2, 168.8, 163.9, 138.5, 130.2, 129.8, 128.9 (d, *J* = 10.5 Hz), 128.5, 128.0, 125.8 (q, *J* = 3.7 Hz), 125.2 (dd, *J* = 7.3, 3.6 Hz), 66.0, 35.4; ^{19}F NMR (377 MHz, DMSO-*d*$_6$) δ -60.9, HRMS: calcd for $C_{17}H_{13}ClF_3N_2O_2S$, $[M+H]^+$ 401.0333, found 401.0321.

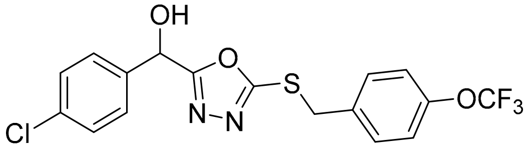

data for **E**$_{18}$: A yellow solid, yield 70%, purity 97.6%, m.p. 124-125 °C. ^{1}H NMR (400 MHz, DMSO-*d*$_6$) δ 7.43 (d, *J* = 12.0 Hz, 4 H, benzene H), 7.22-7.19 (m, 4H, benzene H), 7.07 (d, *J* = 4.0 Hz, 1H, OH), 6.01 (d, *J* = 4.0 Hz, 1H, CH), 4.01 (s, 2H, CH_2); ^{13}C NMR (101 MHz, DMSO-*d*$_6$) δ 170.3, 168.7, 164.0, 147.8, 140.4, 137.7, 130.8, 128.6, 128.0, 120.9, 72.5, 59.4; ^{19}F NMR (377 MHz, DMSO-*d*$_6$) δ -56.8, HRMS: calcd for $C_{17}H_{12}ClF_3N_2O_3S$, $[M+H]^+$ 417.0282, found 417.0280.

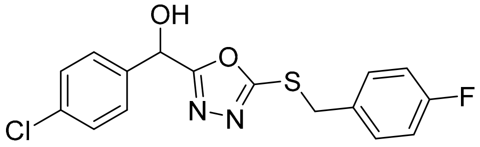

data for **E**$_{19}$: A yellow solid, yield 75%, purity 99.6%, m.p. 74-75 °C. ^{1}H NMR (400 MHz, DMSO-*d*$_6$) δ 7.51-7.46 (m, 2H, benzene H), 7.43-7.38 (m, 2H, benzene H),7.25-7.20 (m, 2H, benzene H), 7.13-7.07 (s, 2H, benzene H), 6.80 (d, *J* = 4.0 Hz, 1H, OH), 6.01 (d, *J* = 4.0 Hz,1H, CH), 4.45 (s, 2H, CH_2); ^{13}C NMR (101 MHz, DMSO-*d*$_6$) δ 168.9, 164.0, 163.4 (d, *J* = 33.3 Hz), 161.0 (d, *J* = 33.4 Hz), 135.8 (d, *J* = 3.0 Hz), 133.3 (d, *J* = 3.1 Hz), 131.5 (d, *J* = 8.4 Hz), 129.1 (d, *J* = 8.4 Hz), 115.9 (d, *J* = 4.0 Hz), 115.7 (d, *J* = 4.0 Hz), 66.1, 35.4; ^{19}F NMR (377 MHz, DMSO-*d*$_6$) δ -114.4; HRMS: calcd for $C_{16}H_{12}ClFN_2O_2S$, $[M+H]^+$ 351.0365, found 351.0357.

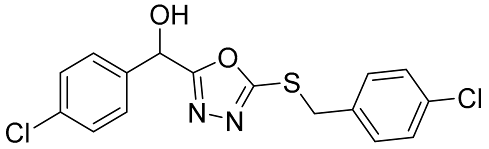

data for **E_{20}**: A white solid, yield 65%, purity 98.2%, m.p. 82-83 ºC. ^{1}H NMR (400 MHz, DMSO-d_6) δ 7.50-7.45 (m, 2H, benzene H), 7.40-7.32 (m, 4H, benzene H), 7.25-7.19 (m, 2H, benzene H), 6.78 (d, J = 8.0 Hz, 1H, OH), 6.00 (d, J = 4.0 Hz, 1H, CH), 4.45 (s, 2H, CH_2); ^{13}C NMR (101 MHz, DMSO-d_6) δ 168.9, 163.9, 162.4 (d, J = 244.2 Hz), 136.2, 135.8 (d, J = 3.0 Hz), 132.8, 131.3, 129.1 (d, J = 8.4 Hz), 128.9, 115.7 (d, J = 21.6 Hz), 66.1, 35.4; HRMS: calcd for $C_{16}H_{12}Cl_2N_2O_2S$, $[M+H]^+$ 367.0069, found 367.0064.

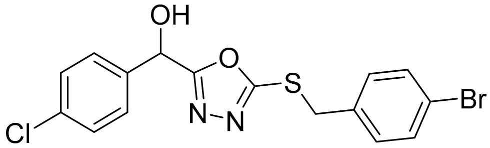

data for **E_{21}**: A white solid, yield 65%, purity 99.7%, m.p. 81-82 ºC. ^{1}H NMR (400 MHz, DMSO-d_6) δ7.45 (s, 4H, benzene H), 7.29-7.26 (m, 4H, benzene H), 6.98 (s, 1H, OH), 6.00 (s, 1H, CH), 4.42 (s, 2H, CH_2); ^{13}C NMR (101 MHz, DMSO-d_6) δ 170.1, 168.7, 163.9, 133.3, 132.3, 131.7 (d, J = 23.8 Hz), 131.3 (d, J = 9.2 Hz), 128.9 (d, J = 12.0 Hz), 128.3 (d, J = 53.7 Hz), 66.0, 35.4; HRMS: calcd for $C_{16}H_{12}BrClN_2O_2S$, $[M+H]^+$ 410.9564, found 410.9561.

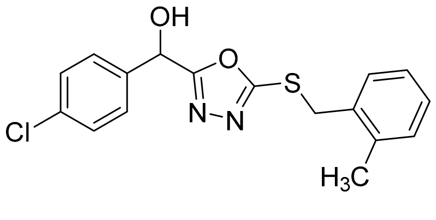

data for **E_{22}**: A yellow liquid, yield 47%, purity 98.5%. ^{1}H NMR (400 MHz, DMSO) δ 7.49-7.44 (m, 4H, phenyl H), 7.21 (d, J = 8.0 Hz, 1H, phenyl H), 7.18 (d, J = 4.0 Hz, 2H, phenyl H), 7.08-7.03 (m, 1H, phenyl H), 6.86 (d, J = 4.0 Hz, 1H, <u>CH</u>OH), 6.03 (d, J = 4.0 Hz, 1H, CH<u>OH</u>), 4.47 (s, 2H, SCH_2), 2.33 (s, 3H, CH_3); ^{13}C NMR (101 MHz, DMSO) δ 168.30, 163.51, 138.13, 136.80, 133.71, 132.87, 130.51, 129.91, 128.51, 128.40, 128.24, 126.08, 65.60, 34.38, 18.75. HRMS: calcd for $C_{17}H_{15}ClN_2O_2S$ $[M+H]^+$ 347.06155, found 347.06152.

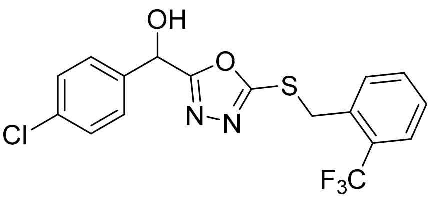

data for **E_{23}**: A yellow liquid, yield 47%, purity 97.5%. ^{1}H NMR (400 MHz, DMSO) δ 7.51-7.49 (m, 1H, phenyl H), 7.46 (s, 4H, phenyl H), 7.44-7.42 (m, 1H, phenyl H), 7.37-7.34 (m, 1H, phenyl H), 7.31-7.27 (m, 1H, phenyl H), 6.85 (d, J = 4.0 Hz, 1H, CHOH), 6.01 (d, J = 4.0 Hz, 1H, CHOH), 4.51 (s, 2H, SCH_2).^{113}C NMR (101 MHz, DMSO) δ 168.49, 163.17, 146.78, 138.08, 132.86, 131.65, 130.17, 128.52, 128.44 (d, J = 4.0Hz), 127.42, 121.39, 120.24, 118.83, 65.59, 30.62. ^{19}F NMR (376 MHz, DMSO) δ -55.94 (d, J = 3.8 Hz). HRMS: calcd for $C_{17}H_{12}ClF_3N_2O_3S$ $[M+H]^+$ 417.02820, found 417.02808.

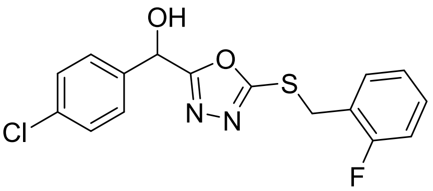

data for **E_{24}**: A white solid, yield 46%, purity 99.3%. ^{1}H NMR (400 MHz, DMSO) δ 7.49-7.44 (m, 4H, phenyl H), 7.39-7.31 (m, 2H, phenyl H), 7.20-7.15 (m, 1H, phenyl H), 7.11-7.07 (m, 1H, phenyl H), 6.86 (d, J = 4.0 Hz, 1H, CHOH), 6.02 (d, J = 4.0 Hz, 1H, CHOH), 4.48 (s, 2H, SCH_2). ^{13}C NMR (101 MHz, DMSO) δ 168.45, 163.22, 161.58, 159.13, 138.07, 132.86, 131.30 (d, J = 3.0 Hz), 130.26 (d, J = 8.1 Hz), 128.44 (d, J = 9.1 Hz), 124.50 (d, J = 4.0 Hz), 123.38 (d, J = 14.1 Hz), 115.53 (d, J = 21.2 Hz), 65.60, 29.72 (d, J = 3.0 Hz). ^{19}F NMR (376 MHz, DMSO) δ -116.67- -116.74 (m). HRMS: calcd for $C_{16}H_{12}ClFN_2O_2S$ $[M+H]^+$ 351.03648, found 351.03647.

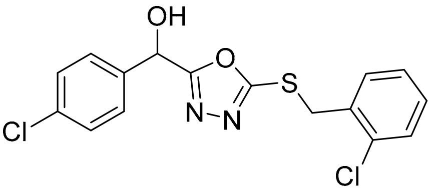

data for **E_{25}**: A yellow solid, yield 48%, purity 99.5%, m.p. 54-56 °C. ^{1}H NMR (400 MHz, DMSO) δ 7.49-7.42 (m, 6H, phenyl H), 7.34-7.30 (m, 1H, phenyl H), 7.25-7.21 (m, 1H, phenyl H), 6.87 (d, J = 4.0 Hz, 1H, CHOH), 6.03 (d, J = 4.0 Hz, 1H, CHOH), 4.53 (s, 2H, SCH_2). ^{13}C NMR (101 MHz,

DMSO) δ 168.50, 163.21, 138.09, 133.71, 133.32, 132.89, 131.44, 130.01, 129.66, 128.50, 128.41, 127.41, 65.61, 34.20. HRMS: calcd for $C_{16}H_{12}Cl_2N_2O_2S$ $[M+H]^+$ 367.00693, found 367.00690.

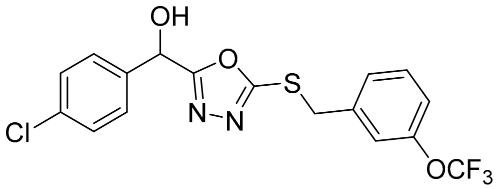

data for **E_{26}**: A yellow liquid, yield 39%, purity 99.6%. ^{1}H NMR (400 MHz, DMSO) δ 7.47-7.42 (m, 4H, phenyl H), 7.40-7.37 (m, 3H, phenyl H), 7.26 (d, J = 8.0 Hz, 1H, phenyl H), 6.85 (d, J = 4.0 Hz, 1H, CHOH), 6.01 (d, J = 4.0 Hz, 1H, CHOH), 4.51 (s, 2H, SCH_2). ^{13}C NMR (101 MHz, DMSO) δ 168.34, 163.51, 148.27 (q, J = 2.0 Hz), 139.58, 138.09, 132.87, 130.46, 128.47, 128.37, 128.09, 121.46, 120.24, 118.77, 65.58, 34.94. ^{19}F NMR (376 MHz, DMSO) δ -56.78 (s). HRMS: calcd for $C_{17}H_{12}ClF_3N_2O_3S$ $[M+H]^+$ 417.02820, found 417.02814.

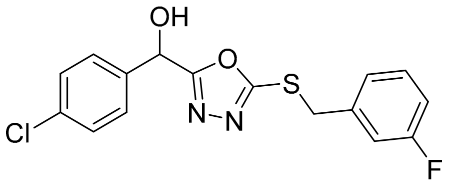

data for **E_{27}**: A yellow liquid, yield 49%, purity 98.7%. ^{1}H NMR (400 MHz, DMSO) δ 7.48-7.43 (m, 4H, phenyl H), 7.34-7.29 (m, 1H, phenyl H), 7.24-7.17 (m, 2H, phenyl H), 7.12-7.09 (m, 1H, phenyl H), 6.85 (d, J = 4.0 Hz, 1H, CHOH), 6.02 (d, J = 4.0 Hz, 1H, CHOH), 4.47 (s, 2H, SCH_2). ^{13}C NMR (101 MHz, DMSO) δ 168.32, 163.53, 163.16, 160.73, 139.47 (d, J = 8.1 Hz), 138.08, 132.86, 130.47 (d, J = 8.1 Hz), 128.42 (d, J = 11.1 Hz), 125.09 (d, J = 2.0 Hz), 115.77 (d, J = 22.2 Hz), 114.63 (d, J = 21.2 Hz), 65.57, 35.10 (d, J = 1.0 Hz). ^{19}F NMR (376 MHz, DMSO) δ -112.87 - -112.99 (m). HRMS: calcd for $C_{16}H_{12}FN_2O_2S$ $[M+H]^+$ 351.03648, found 351.03647.

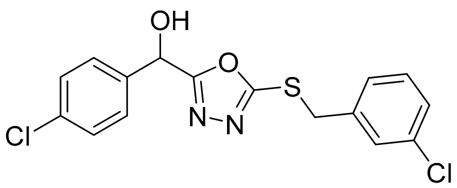

data for **E_{28}**: A yellow liquid, yield 47%, purity 99.6%. ^{1}H NMR (400 MHz, DMSO) δ 7.48-7.43 (m, 5H, phenyl H), 7.34-7.29 (m, 3H, phenyl H), 6.86 (d, J = 4.0 Hz, 1H, CHOH), 6.01 (d, J = 4.0 Hz, 1H, CHOH), 4.46 (s, 2H, SCH_2). ^{13}C NMR (101 MHz, DMSO) δ 168.33, 163.52, 139.25, 138.07,

133.01, 132.87, 130.34, 128.83, 128.49, 128.37, 127.72, 127.66, 65.58, 34.98. HRMS: calcd for $C_{16}H_{12}Cl_2N_2O_2S$ $[M+H]^+$ 367.00693, found 367.00693.

2. Copies of ^{1}H, ^{13}C NMR, ^{19}F NMR, HRMS and HPLC of the title compounds

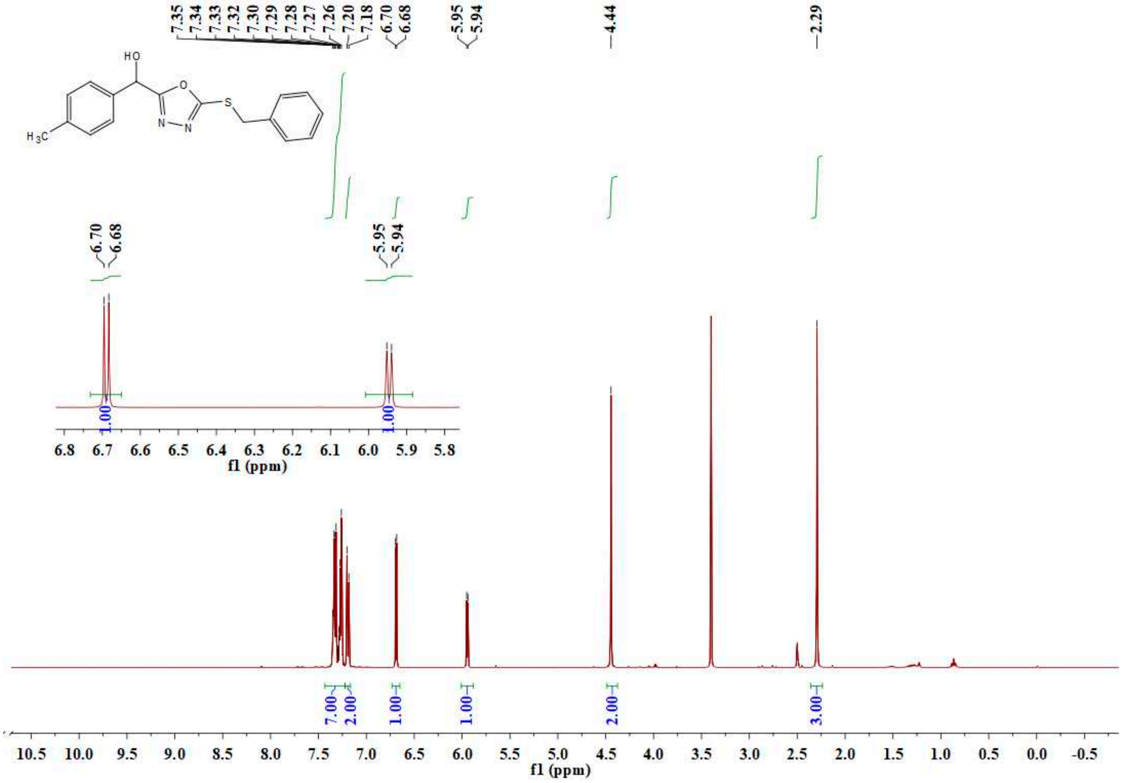

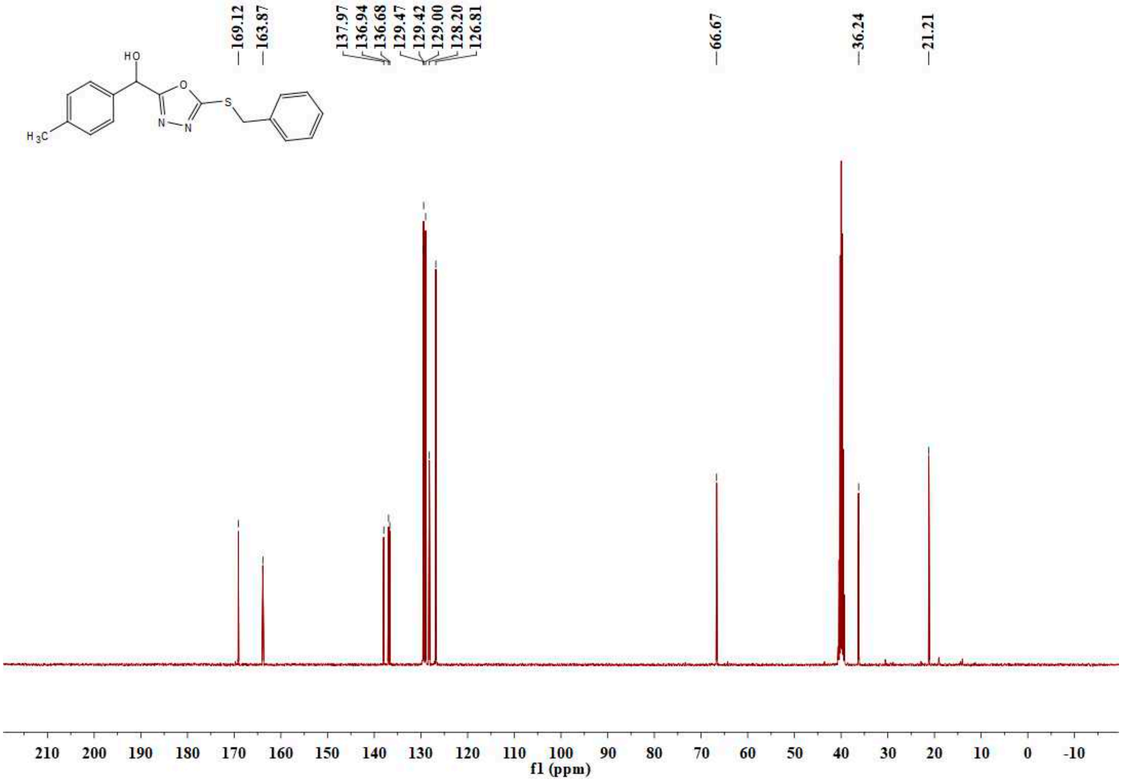

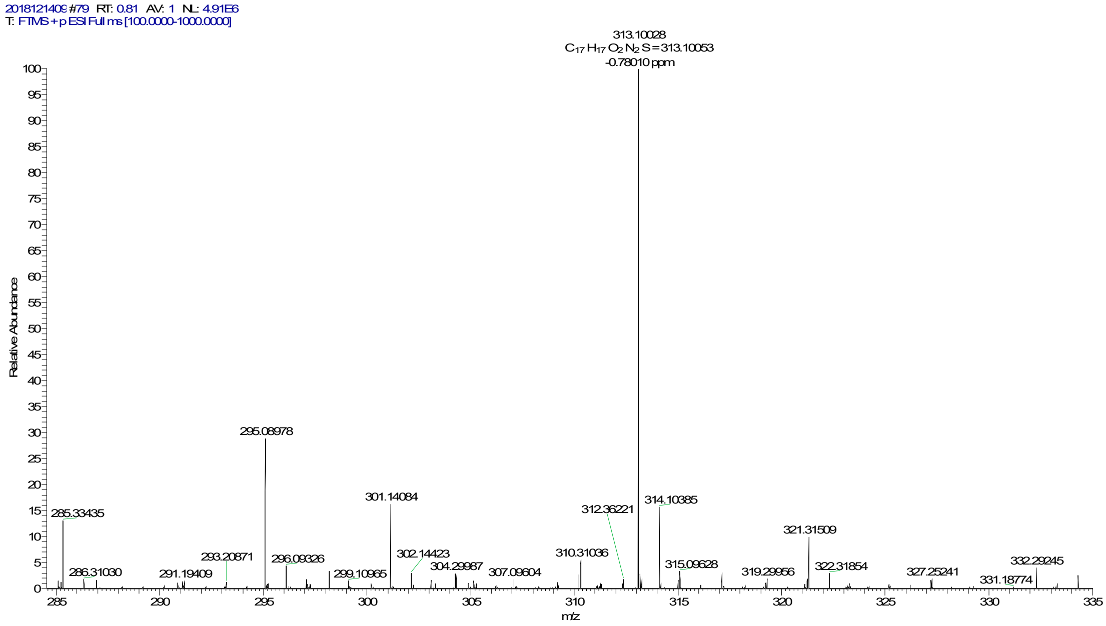

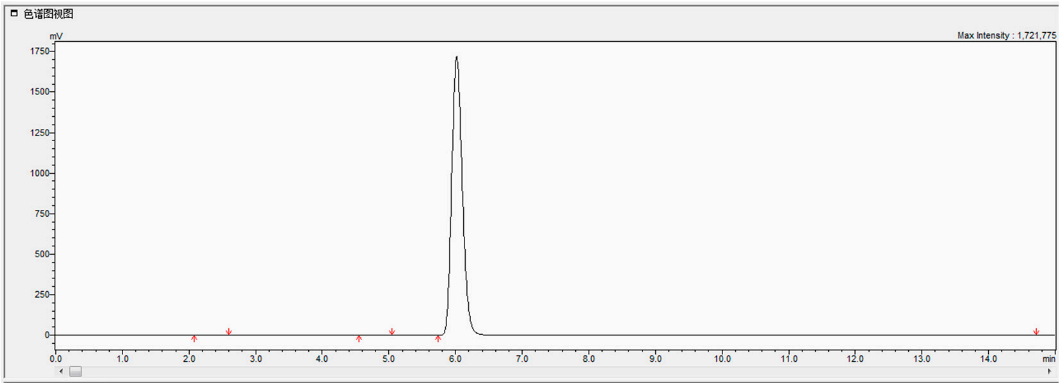

结果视图 - 峰表

峰表 | 化合物 | 组 | 校准曲线

峰号	保留时间	面积	峰宽 (高度50%)	分离度 (USP)	拖尾因	高度	面积%	理论塔板数 (USP)	拖尾因子
1	2.289	24141	0.258	--	0.000	1499	0.127	689	--
2	4.795	12541	0.152	8.063	0.000	1125	0.066	4937	--
3	6.015	18880295	0.169	4.300	1.176	1721883	99.464	6665	1.211
4	19.093	64987	0.958	14.012	0.955	1100	0.342	2361	0.929
总计		18981964				1725607	100.000		

Figure S1. ^{1}H NMR, ^{13}C NMR, HRMS, and HPLC for **E$_1$**.

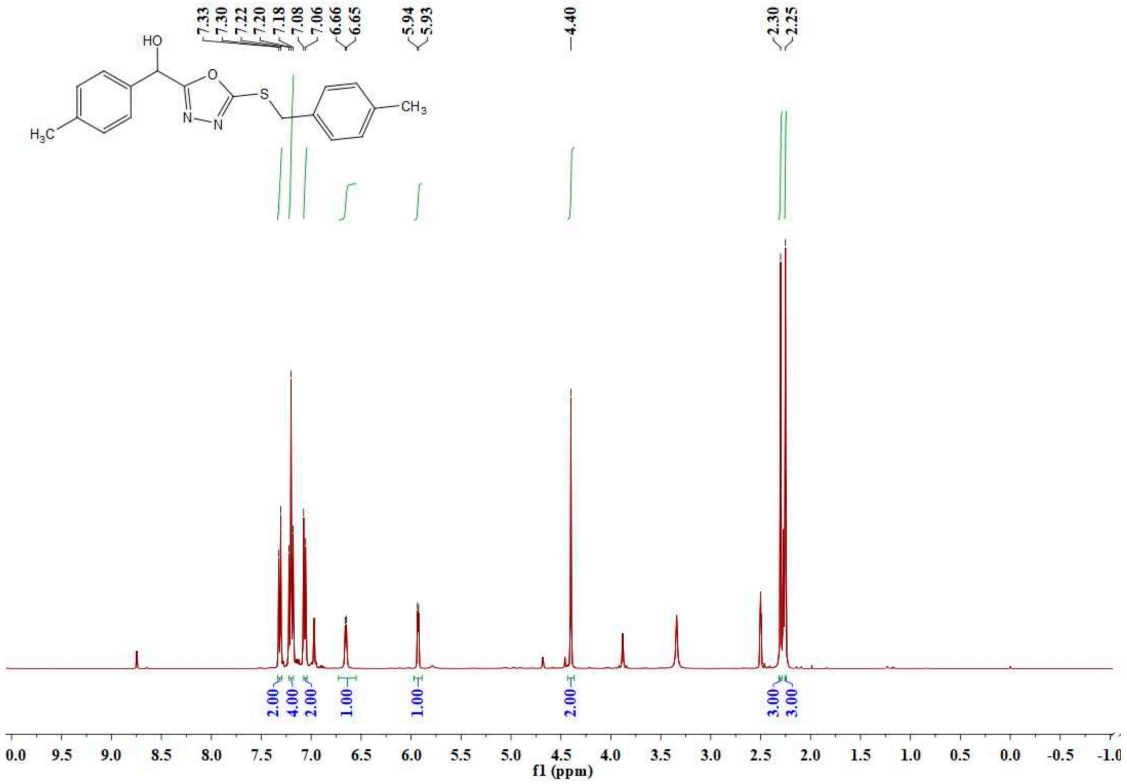
7.33
7.30
7.22
7.20
7.18
7.08
7.06
6.66
6.65
5.94
5.93
4.40
2.30
2.25
HO
O
S
N–N
H_3C
CH_3
2.00
4.00
2.00
1.00
1.00
2.00
3.00
3.00
f1 (ppm)

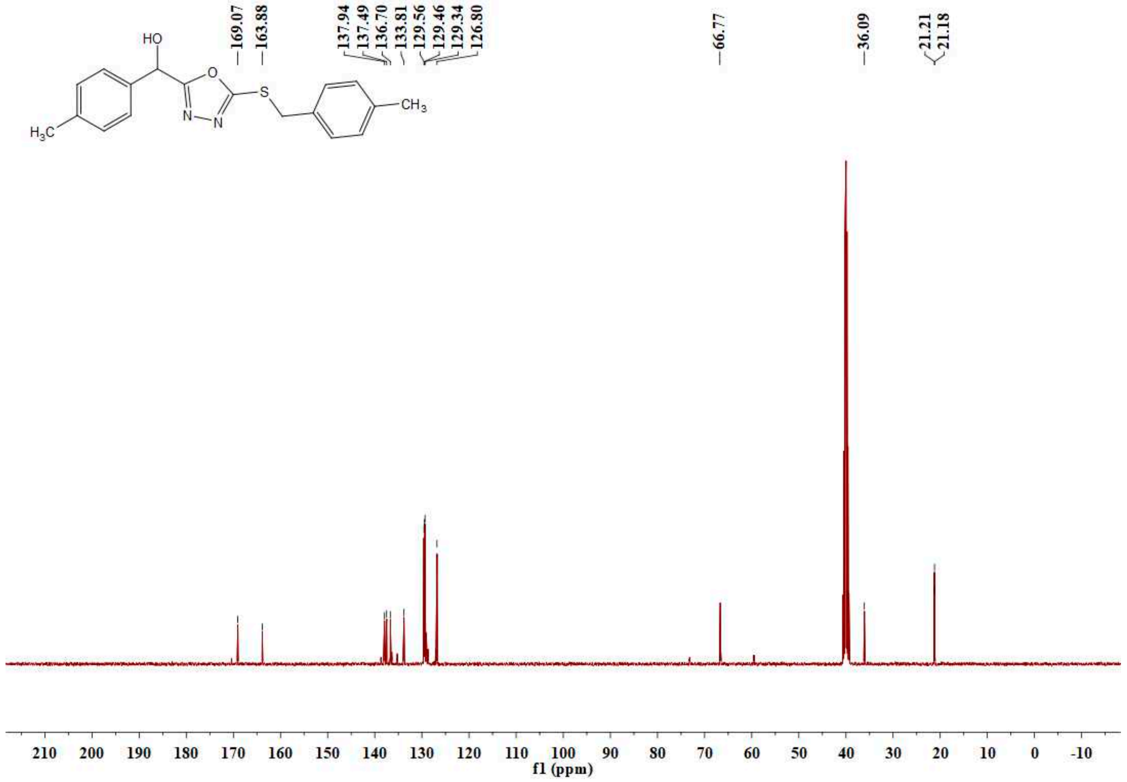
169.07
163.88
137.94
137.49
136.70
133.81
129.56
129.46
129.34
126.80
66.77
36.09
21.21
21.18
HO
O
S
N–N
H_3C
CH_3
f1 (ppm)

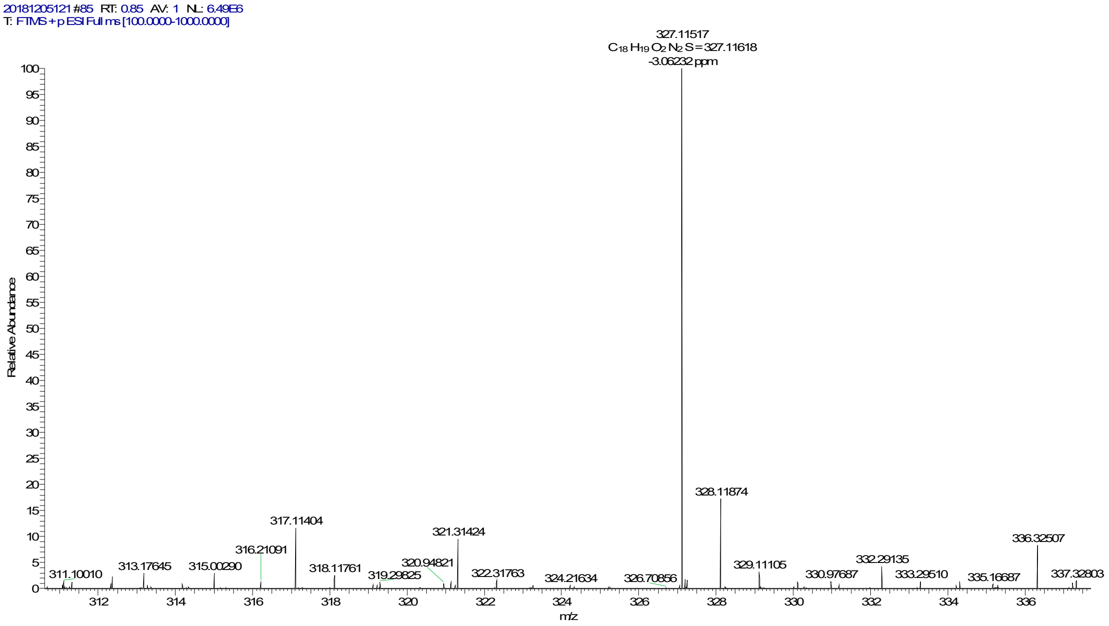

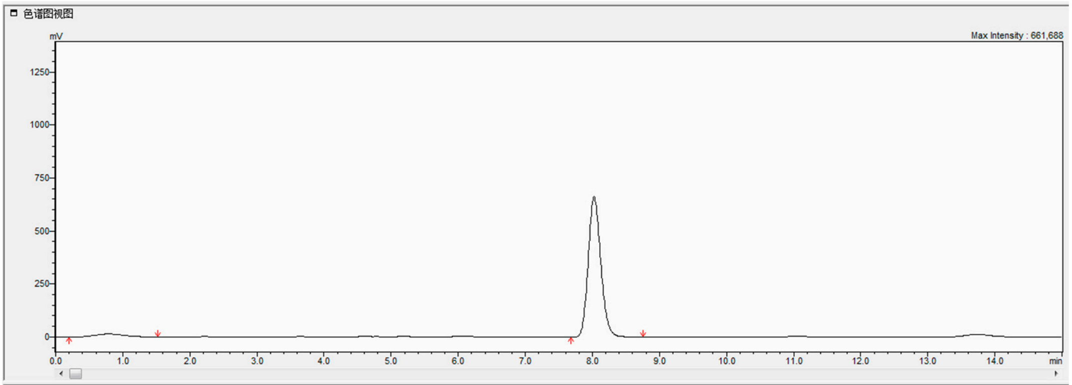

结果视图 - 峰表

峰表 | 化合物 | 组 | 校准曲线

峰号	保留时间	面积	峰宽 (高度50%)	分离度 (USP)	拖尾因	高度	面积%	理论塔板数 (USP)	拖尾因子
1	0.784	414984	0.470	--	1.111	13855	4.587	15	1.122
2	8.025	8632165	0.202	12.567	1.146	656349	95.413	8442	1.175
总计		9047149				670204	100.000		

Figure S2. ^{1}H NMR, ^{13}C NMR, ^{19}F NMR, HRMS and HPLC for **E_2**.

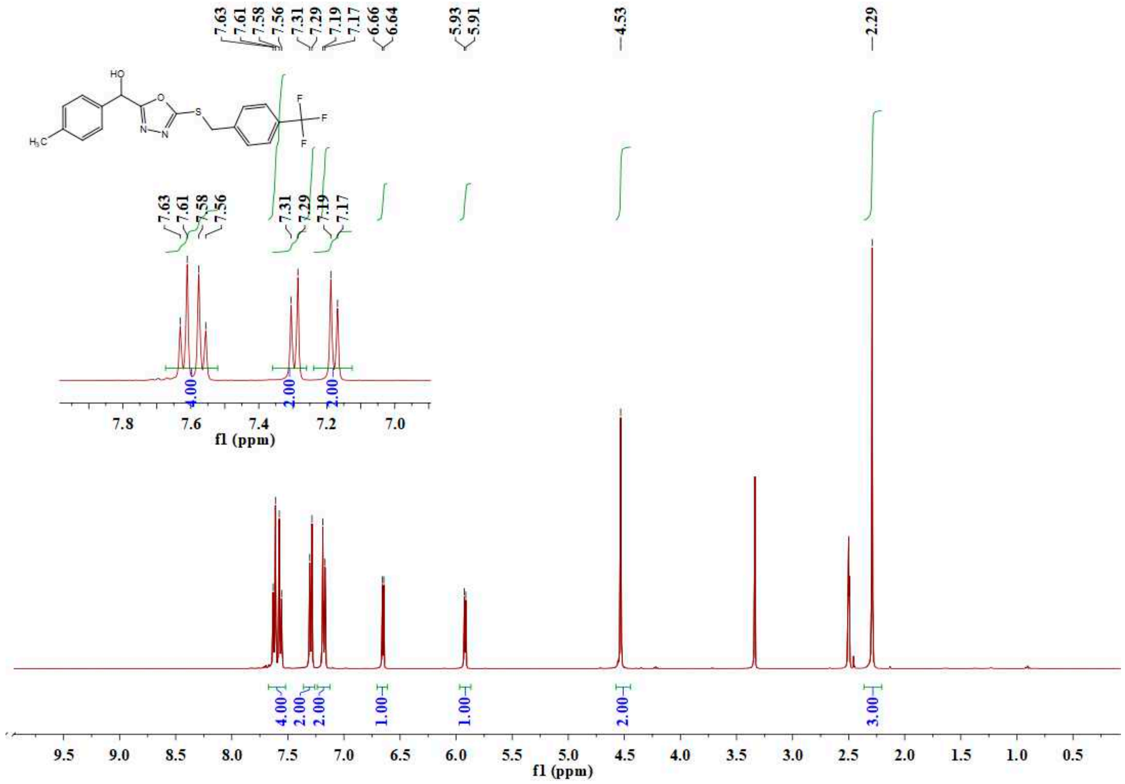
7.63
7.61
7.58
7.56
7.31
7.29
7.19
7.17
6.66
6.64
5.93
5.91
4.53
2.29
HO
O
S
F
F
F
N
N
H3C
4.00
2.00
2.00
1.00
1.00
2.00
3.00
f1 (ppm)

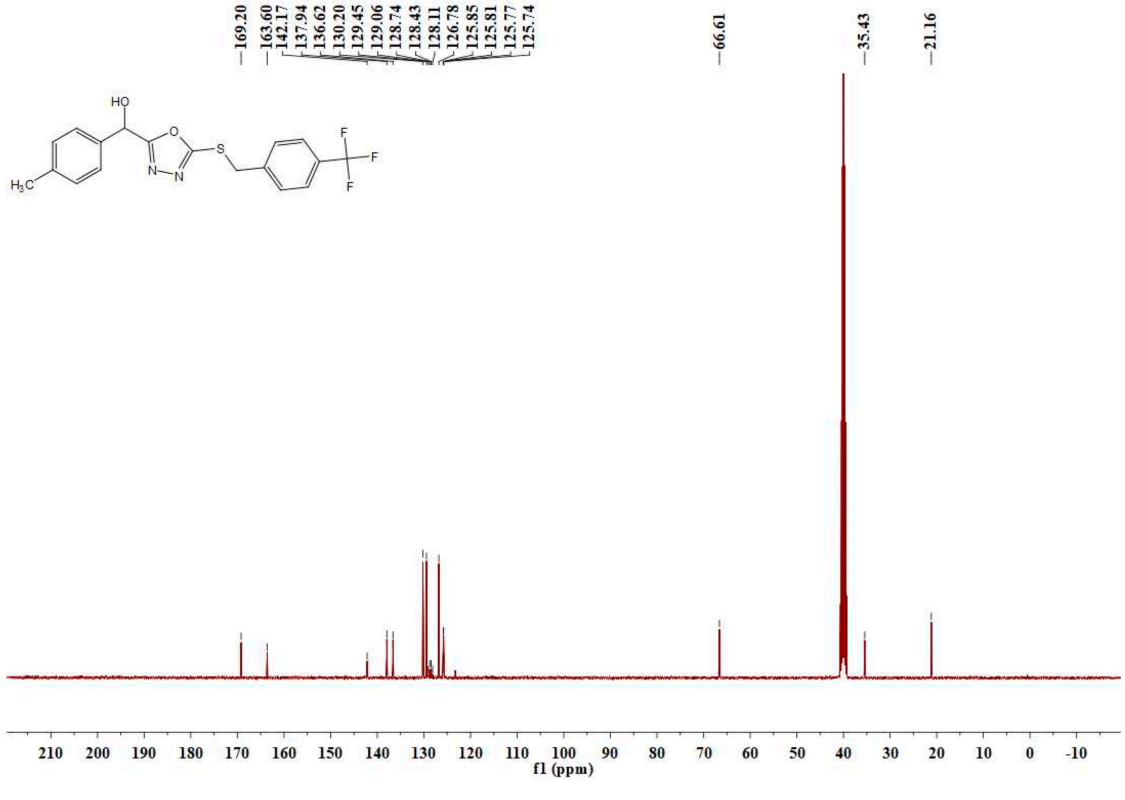
169.20
163.60
142.17
137.94
136.62
130.20
129.45
129.06
128.74
128.43
128.11
126.78
125.85
125.81
125.77
125.74
66.61
35.43
21.16
HO
O
S
F
F
F
N
N
H3C
f1 (ppm)

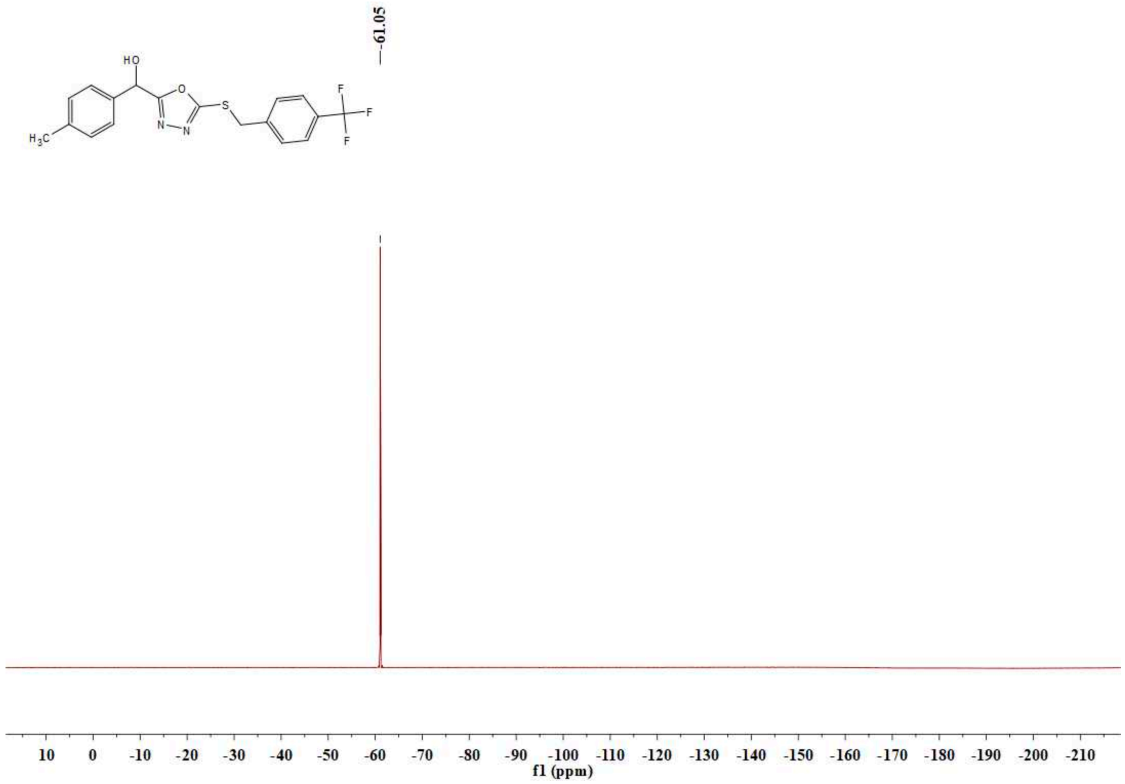

-61.05
HO
S
O
N
N
F
F
F
H3C
10 0 -10 -20 -30 -40 -50 -60 -70 -80 -90 -100 -110 -120 -130 -140 -150 -160 -170 -180 -190 -200 -210
f1 (ppm)

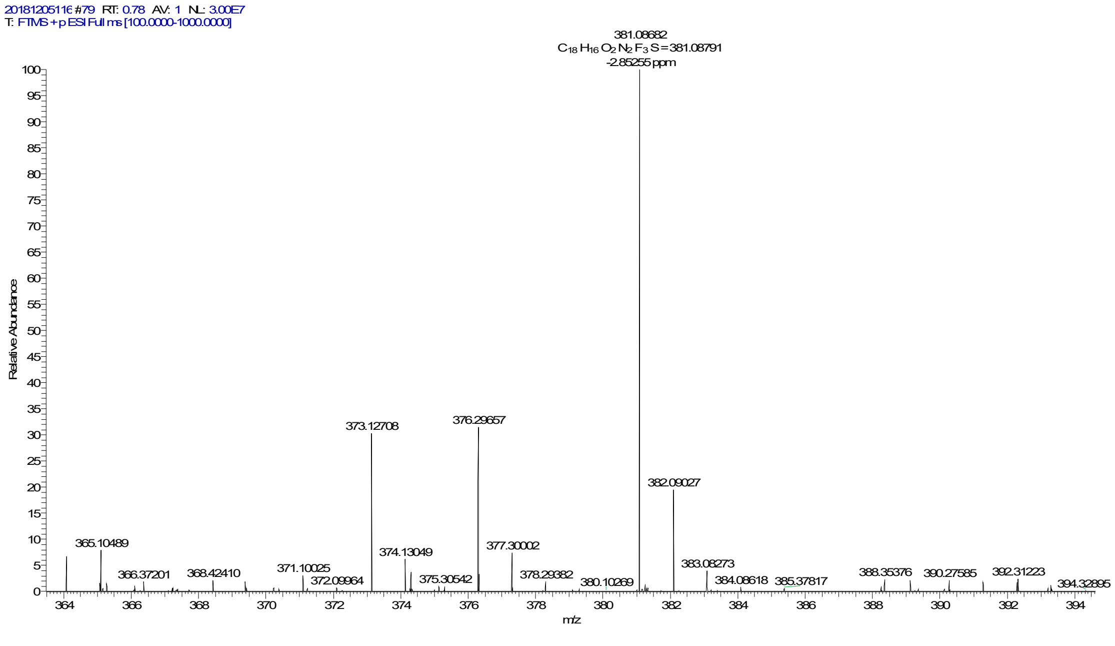

20181205116 #79 RT: 0.78 AV: 1 NL: 3.00E7
T: FTMS + p ESI Full ms [100.0000-1000.0000]
381.08682
C18 H16 O2 N2 F3 S = 381.08791
-2.85255 ppm
Relative Abundance
373.12708
376.29657
382.09027
365.10489
374.13049
377.30002
383.08273
366.37201
368.42410
371.10025
372.09964
375.30542
378.29382
380.10269
384.08618
385.37817
388.35376
390.27585
392.31223
394.32895
m/z

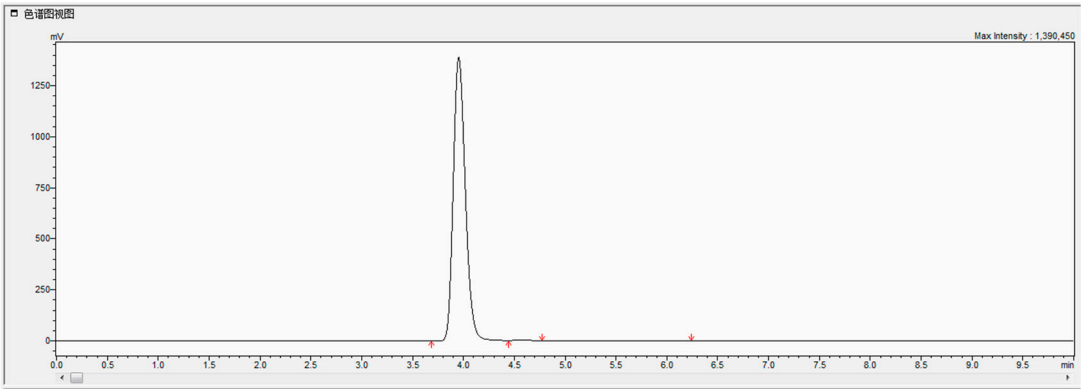

结果视图 - 峰表

峰表 | 化合物 | 组 | 校准曲线

峰号	保留时间	面积	峰宽 (高度50%)	分离度 (USP)	拖尾因	高度	面积%	理论塔板数 (USP)	拖尾因子
1	3.955	11585233	0.128	--	1.184	1390452	99.659	4870	1.225
2	4.573	39697	0.134	2.681	1.174	4787	0.341	6099	1.187
总计		11624930				1395239	100.000		

Figure S3. ^{1}H NMR, ^{13}C NMR, ^{19}F NMR, HRMS and HPLC for **of E$_3$.**

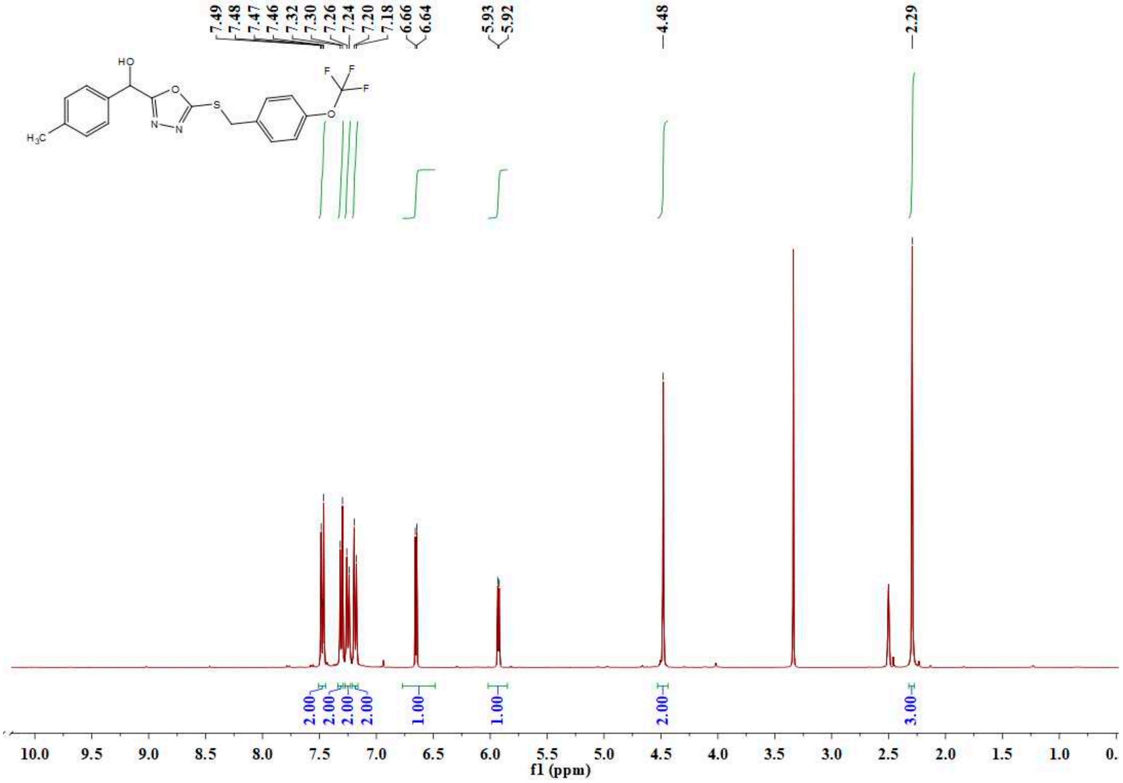

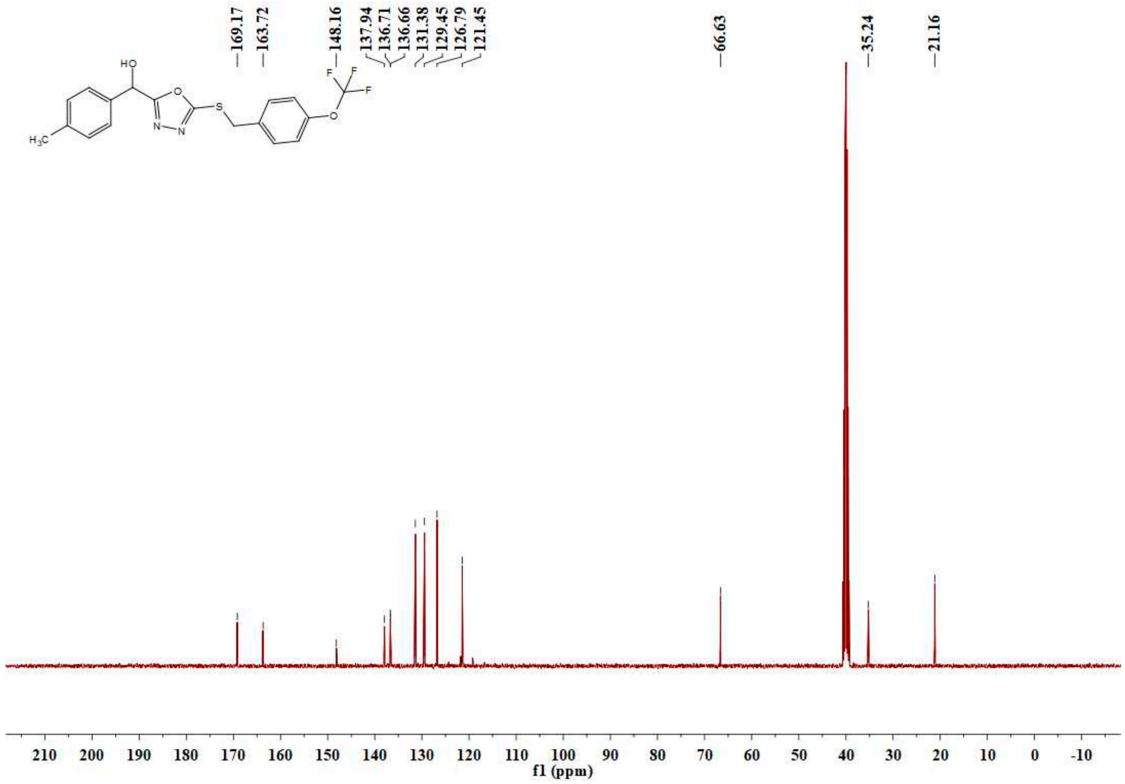
169.17
163.72
148.16
137.94
136.71
136.66
131.38
129.45
126.79
121.45
66.63
35.24
21.16
HO
O
N
N
S
F
F
F
O
H_3C
210 200 190 180 170 160 150 140 130 120 110 100 90 80 70 60 50 40 30 20 10 0 -10
f1 (ppm)

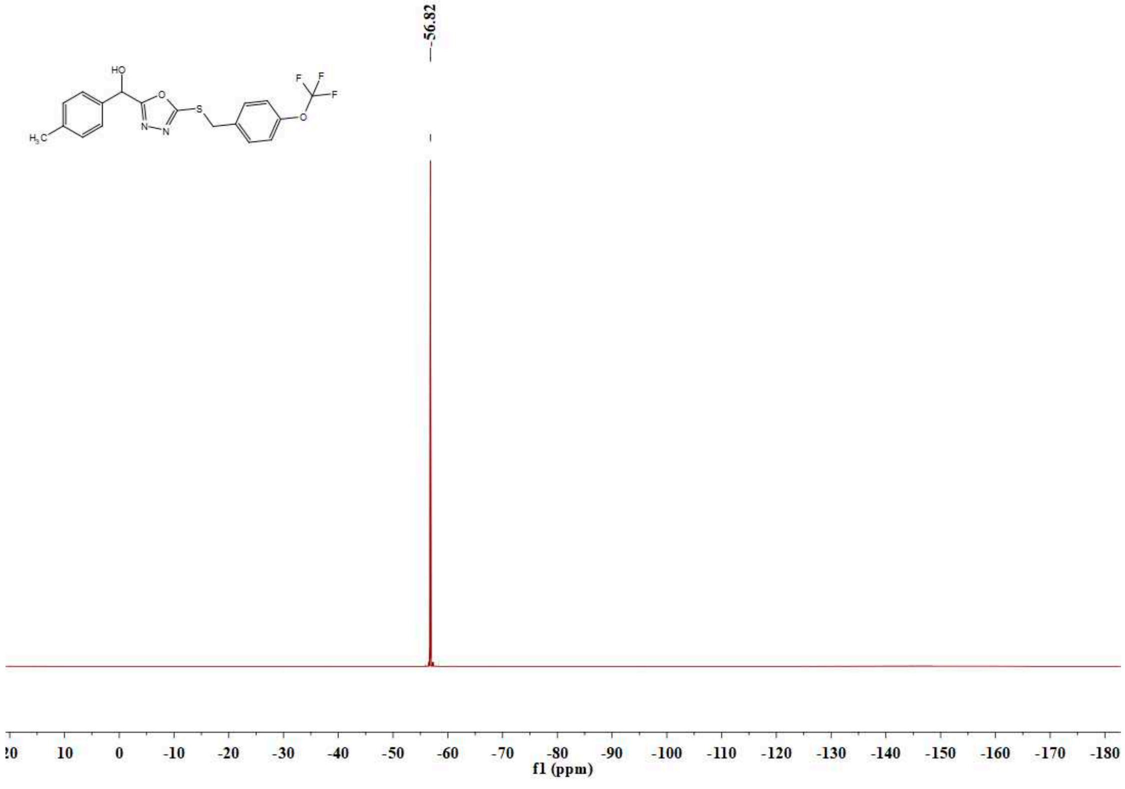
-56.82
HO
O
N
N
S
F
F
F
O
H_3C
10 0 -10 -20 -30 -40 -50 -60 -70 -80 -90 -100 -110 -120 -130 -140 -150 -160 -170 -180
f1 (ppm)

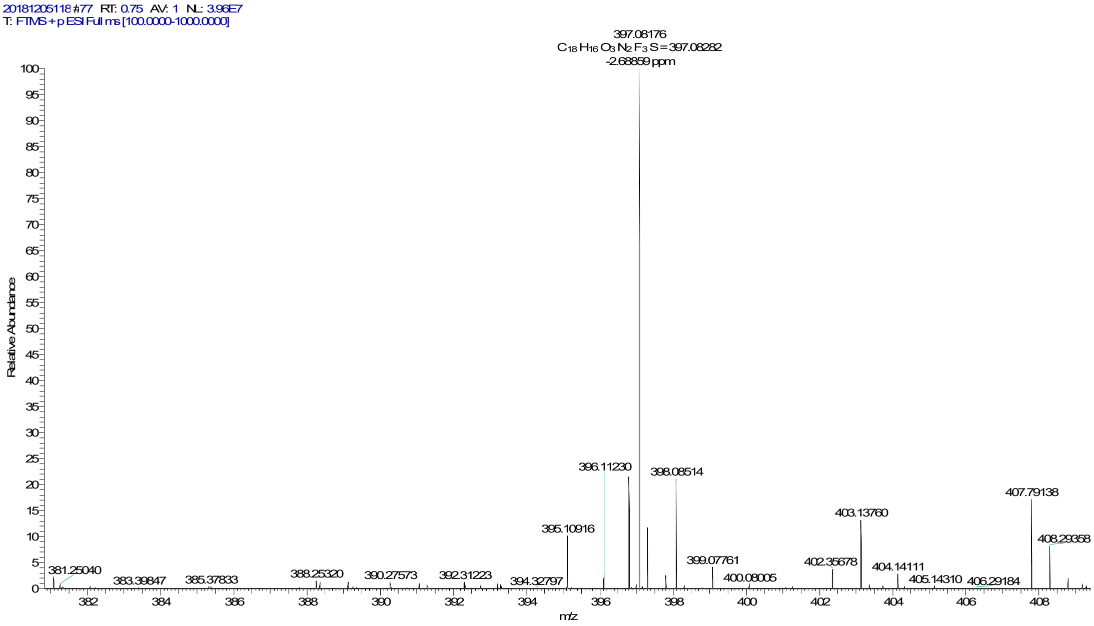

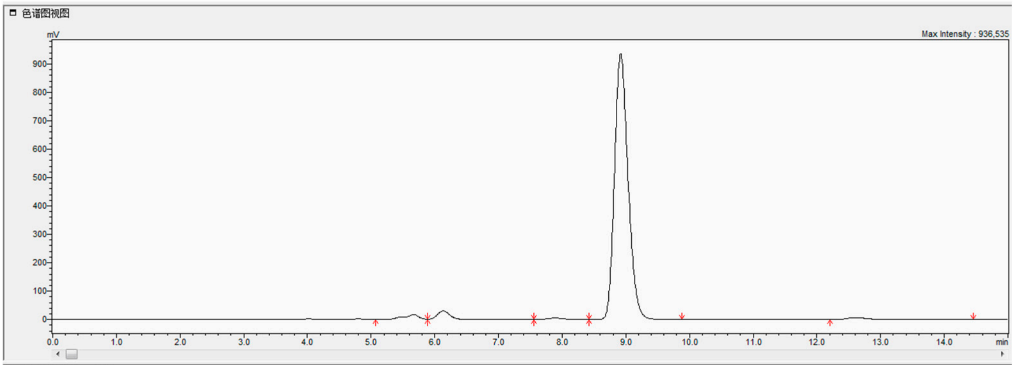

结果视图 - 峰表

峰表 | 化合物 | 组 | 校准曲线

峰号	保留时间	面积	峰宽 (高度50%)	分离度 (USP)	拖尾因	高度	面积%	理论塔板数 (USP)	拖尾因子
1	5.665	301952	0.267	--	0.758	17857	2.035	4252	--
2	6.130	429963	0.213	1.298	1.106	30285	2.897	4423	1.116
3	7.893	86808	0.245	4.432	1.023	5229	0.585	5467	1.030
4	8.919	13878027	0.228	2.498	1.170	936534	93.518	8177	1.201
5	12.612	143266	0.305	8.026	1.174	7025	0.965	9205	1.200
总计		14840016				996929	100.000		

Figure S4. ^{1}H NMR, ^{13}C NMR, ^{19}F NMR, HRMS and HPLC for **of E$_4$.**

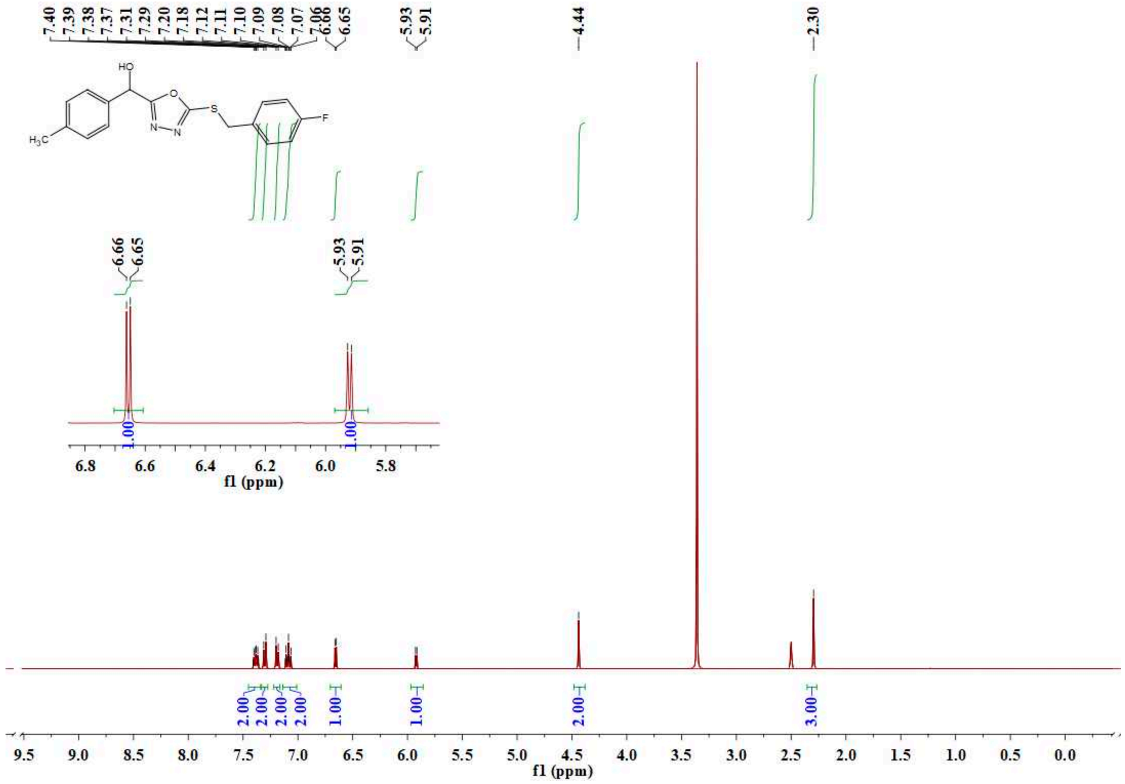
7.40 7.39 7.38 7.37 7.31 7.29 7.20 7.18 7.12 7.11 7.10 7.09 7.08 7.07 7.06 6.66 6.65
5.93 5.91
4.44
2.30
HO
O
S
F
N
N
H_3C
6.66 6.65
5.93 5.91
1.00
1.00
6.8 6.6 6.4 6.2 6.0 5.8
f1 (ppm)
2.00 2.00 2.00 2.00 1.00 1.00 2.00 3.00
9.5 9.0 8.5 8.0 7.5 7.0 6.5 6.0 5.5 5.0 4.5 4.0 3.5 3.0 2.5 2.0 1.5 1.0 0.5 0.0
f1 (ppm)

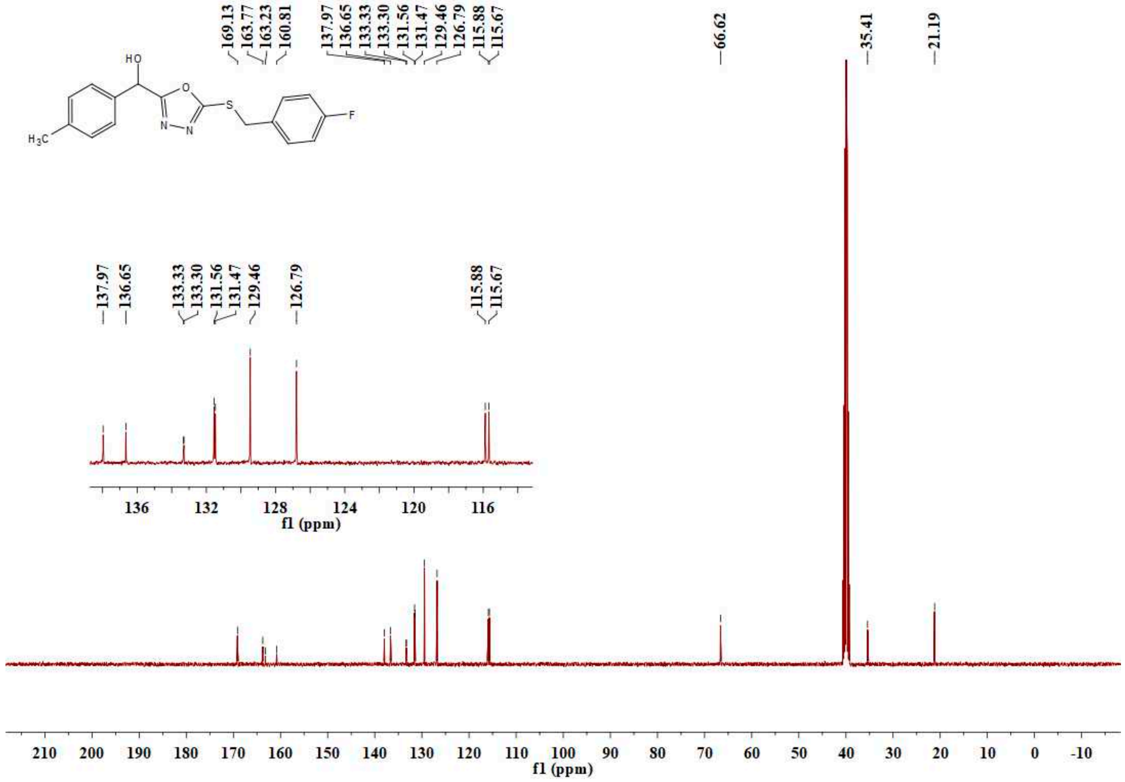
169.13 163.77 163.23 160.81
137.97 136.65 133.33 133.30 131.56 131.47 129.46 126.79 115.88 115.67
66.62
35.41
21.19
HO
O
S
F
N
N
H_3C
137.97 136.65 133.33 133.30 131.56 131.47 129.46 126.79 115.88 115.67
136 132 128 124 120 116
f1 (ppm)
210 200 190 180 170 160 150 140 130 120 110 100 90 80 70 60 50 40 30 20 10 0 -10
f1 (ppm)

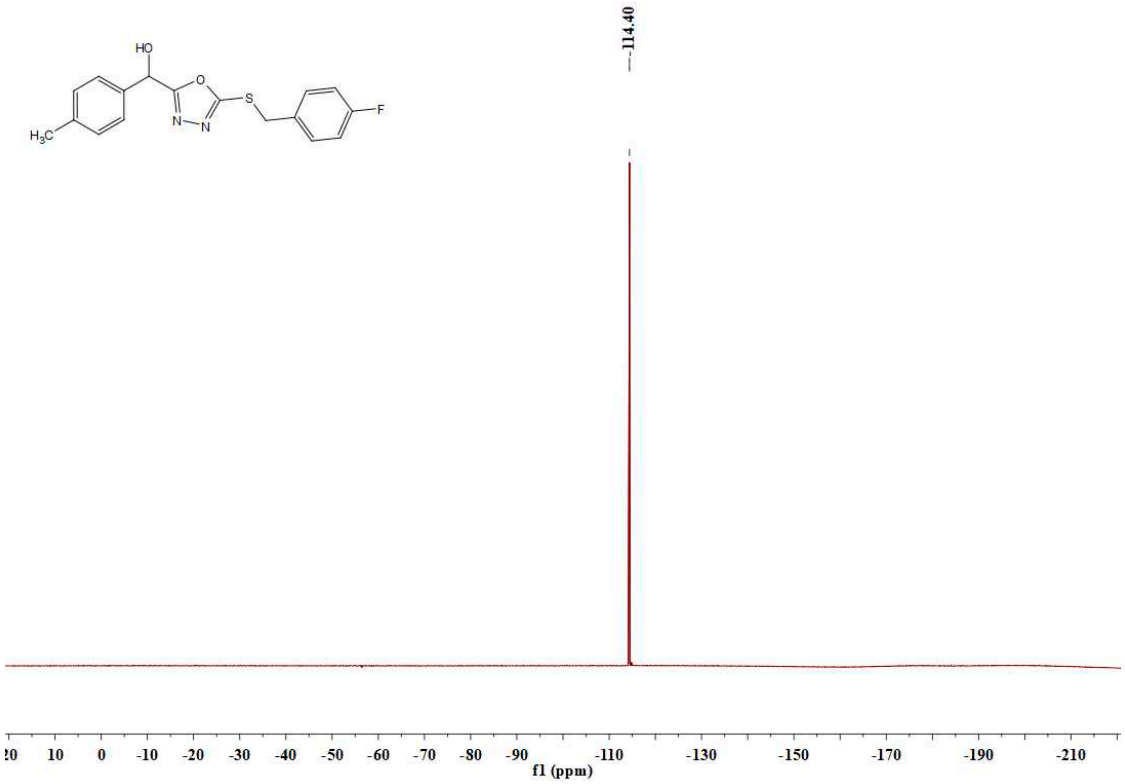

HO
O
S
F
N
N
H3C
-114.40
f1 (ppm)

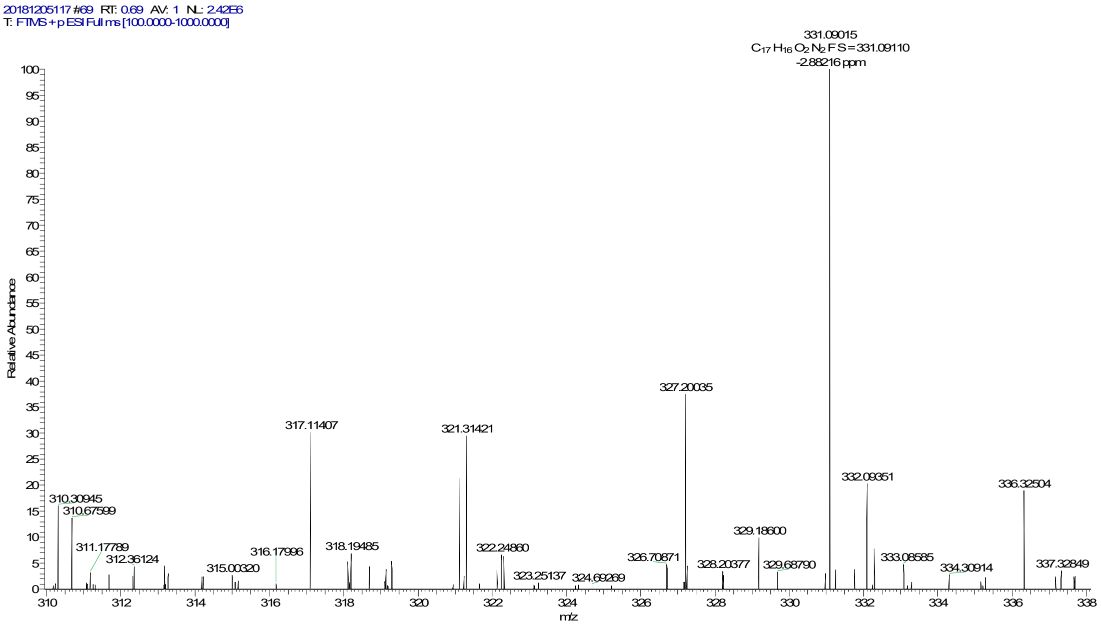

20181205117 #69 RT: 0.69 AV: 1 NL: 2.42E6
T: FTMS + p ESI Full ms [100.0000-1000.0000]
331.09015
C17 H16 O2 N2 F S = 331.09110
-2.88216 ppm
Relative Abundance
m/z
327.20035
317.11407
321.31421
332.09351
336.32504
310.30945
310.67599
329.18600
311.17789
312.36124
316.17996
318.19485
322.24860
326.70871
315.00320
328.20377
329.68790
333.08585
334.30914
337.32849
323.25137
324.69269

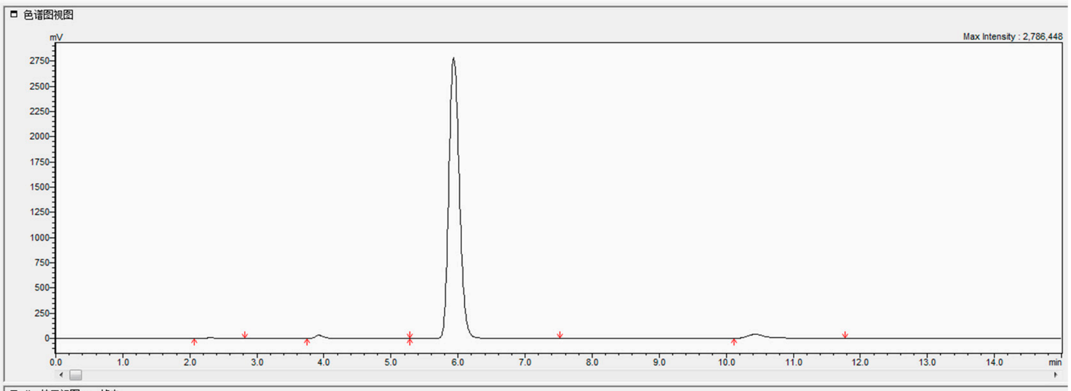

结果视图 - 峰表

峰表 | 化合物 | 组 | 校准曲线

峰号	保留时间	面积	峰宽 (高度50%)	分离度 (USP)	拖尾因	高度	面积%	理论塔板数 (USP)	拖尾因子
1	2.302	89542	0.080	--	2.317	10761	0.287	3420	2.665
2	3.928	312351	0.134	8.227	1.194	31302	1.003	4369	1.182
3	5.936	29919381	0.167	7.649	1.139	2771234	96.054	6830	1.172
4	10.429	827187	0.272	11.863	1.591	40703	2.656	7873	1.622
总计		31148460				2853999	100.000		

Figure S5. ^{1}H NMR, ^{13}C NMR, ^{19}F NMR, HRMS and HPLC for **E_5**.

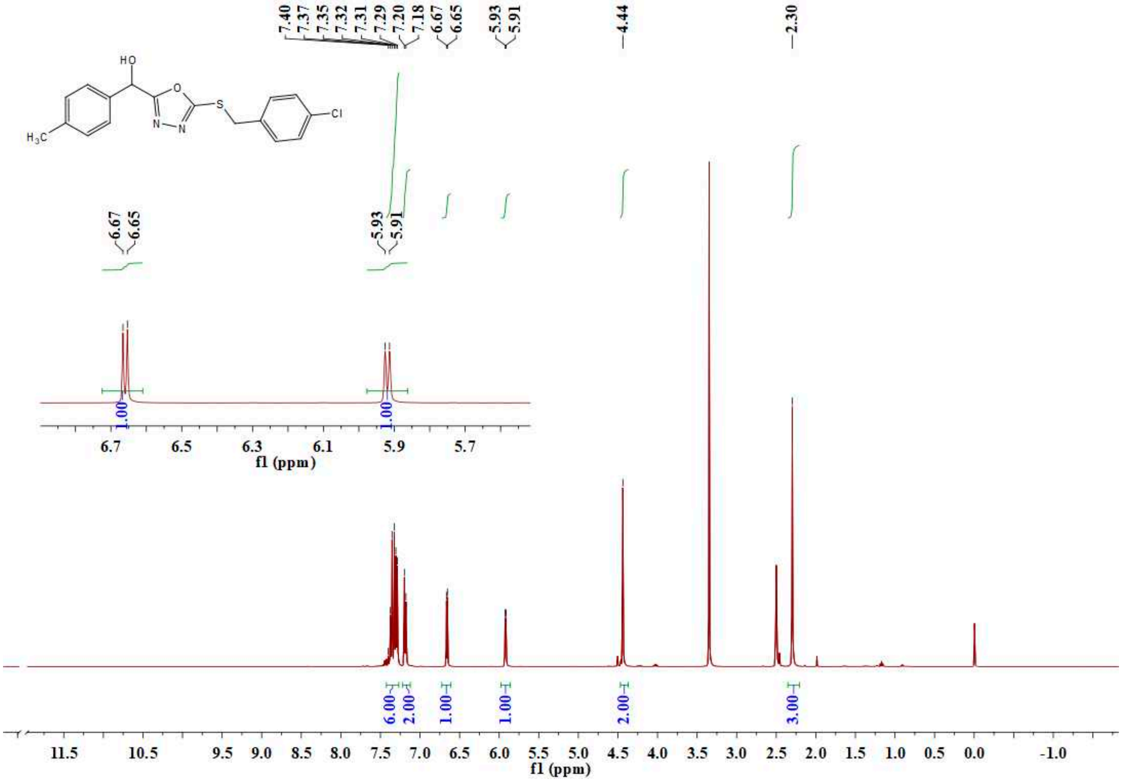

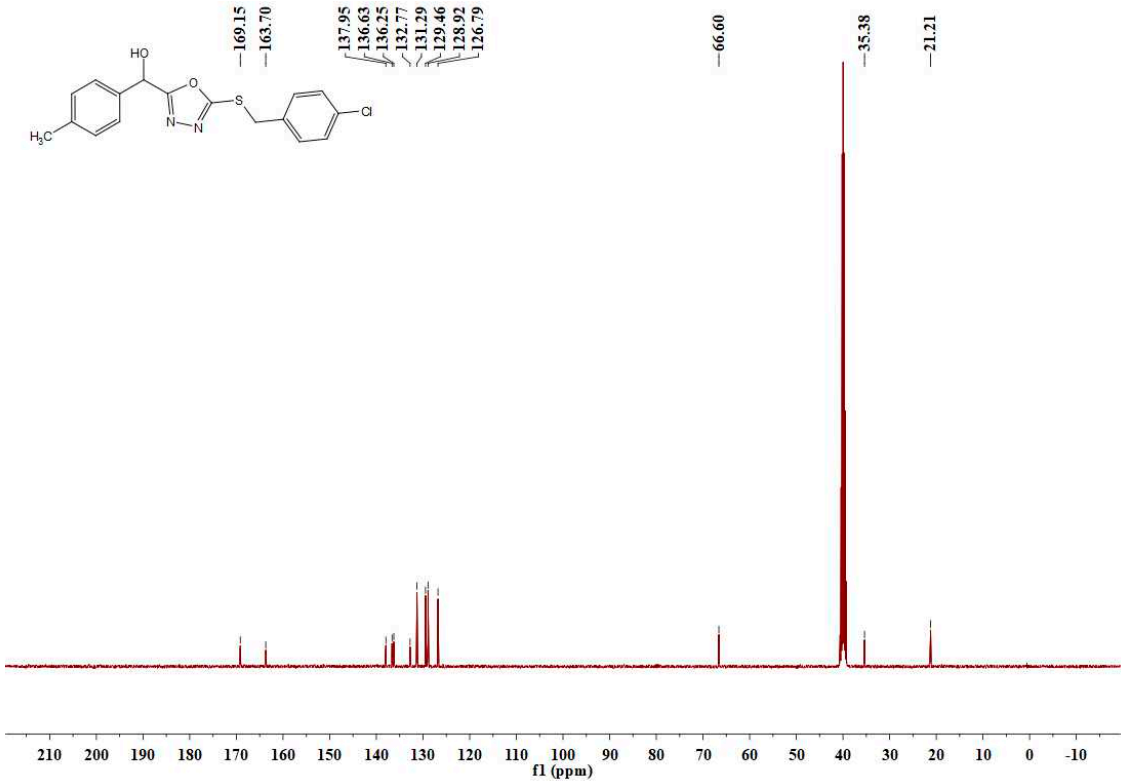
169.15
163.70
137.95
136.63
136.25
132.77
131.29
129.46
128.92
126.79
66.60
35.38
21.21
HO
O
S
Cl
N
N
H3C
210 200 190 180 170 160 150 140 130 120 110 100 90 80 70 60 50 40 30 20 10 0 -10
f1 (ppm)

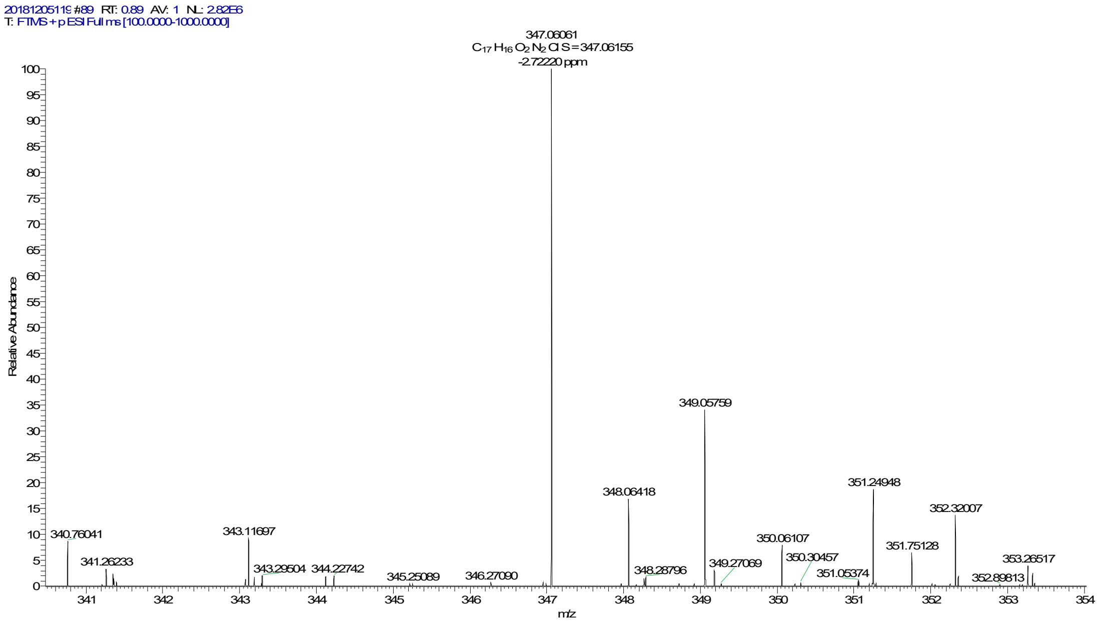
20181205119 #89 RT: 0.89 AV: 1 NL: 2.82E6
T: FTMS + p ESI Full ms [100.0000-1000.0000]
347.06061
C17 H16 O2 N2 Cl S = 347.06155
-2.72220 ppm
349.05759
348.06418
351.24948
352.32007
340.76041
343.11697
350.06107
351.75128
341.26233
350.30457
353.26517
349.27069
343.29504
344.22742
348.28796
351.05374
345.25089
346.27090
352.89813
Relative Abundance
m/z

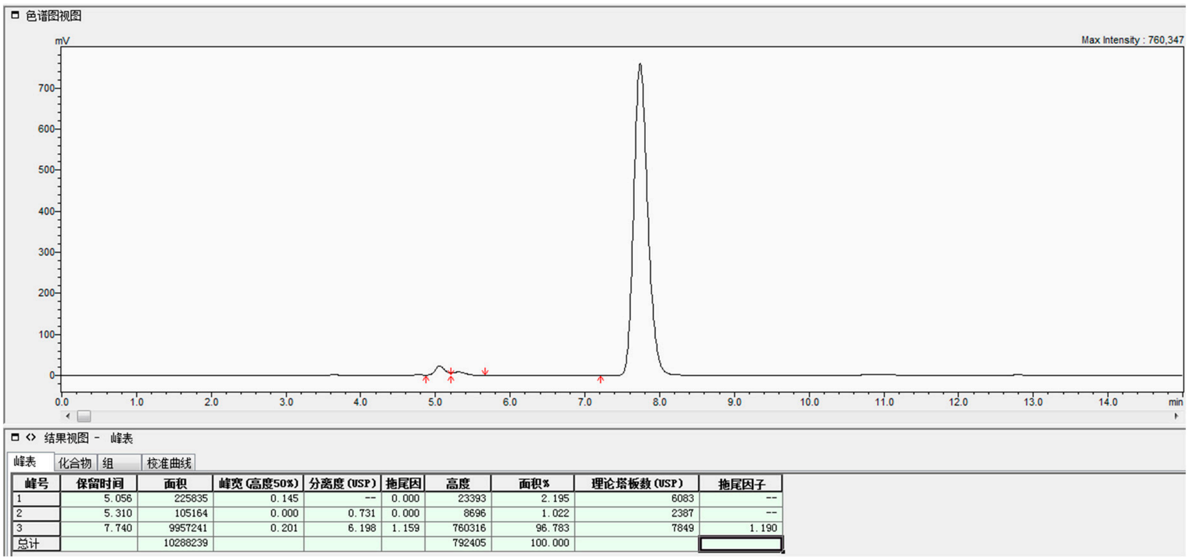

峰号	保留时间	面积	峰宽 (高度50%)	分离度 (USP)	拖尾因	高度	面积%	理论塔板数 (USP)	拖尾因子
1	5.056	225835	0.145	--	0.000	23393	2.195	6083	--
2	5.310	105164	0.000	0.731	0.000	8696	1.022	2387	--
3	7.740	9957241	0.201	6.198	1.159	760316	96.783	7849	1.190
总计		10288239				792405	100.000		

Figure S6. ^{1}H NMR, ^{13}C NMR, HRMS, and HPLC for **E$_6$.**

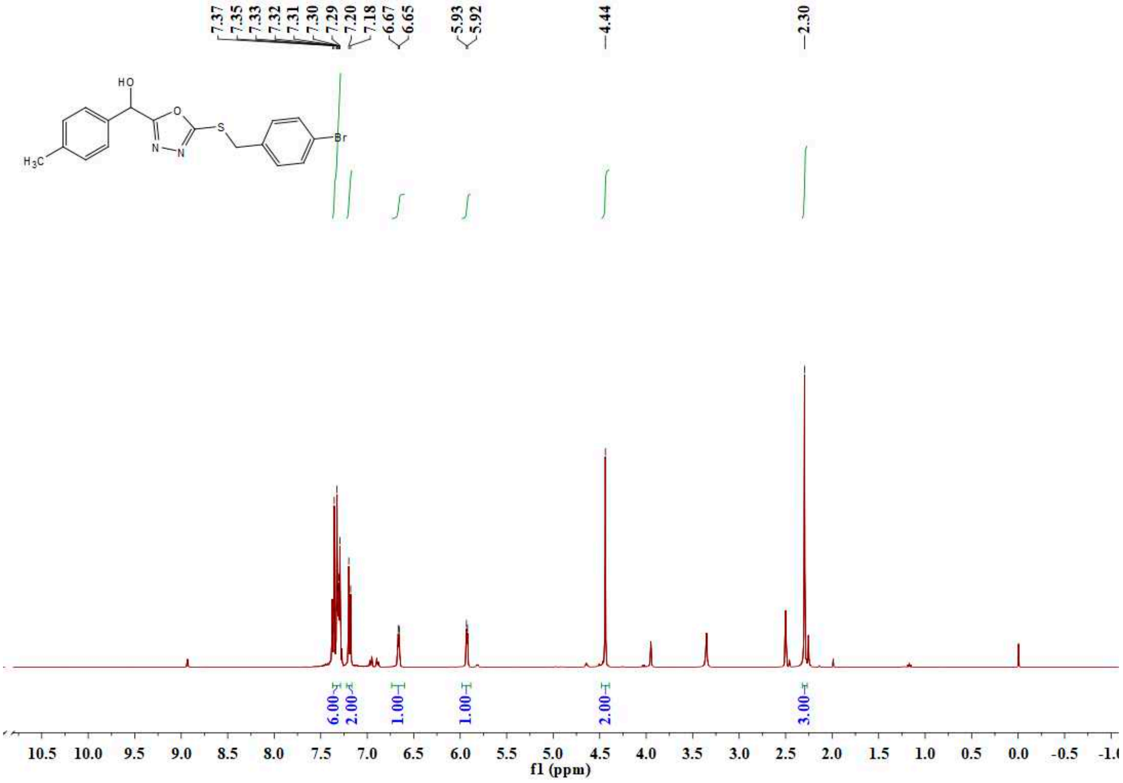

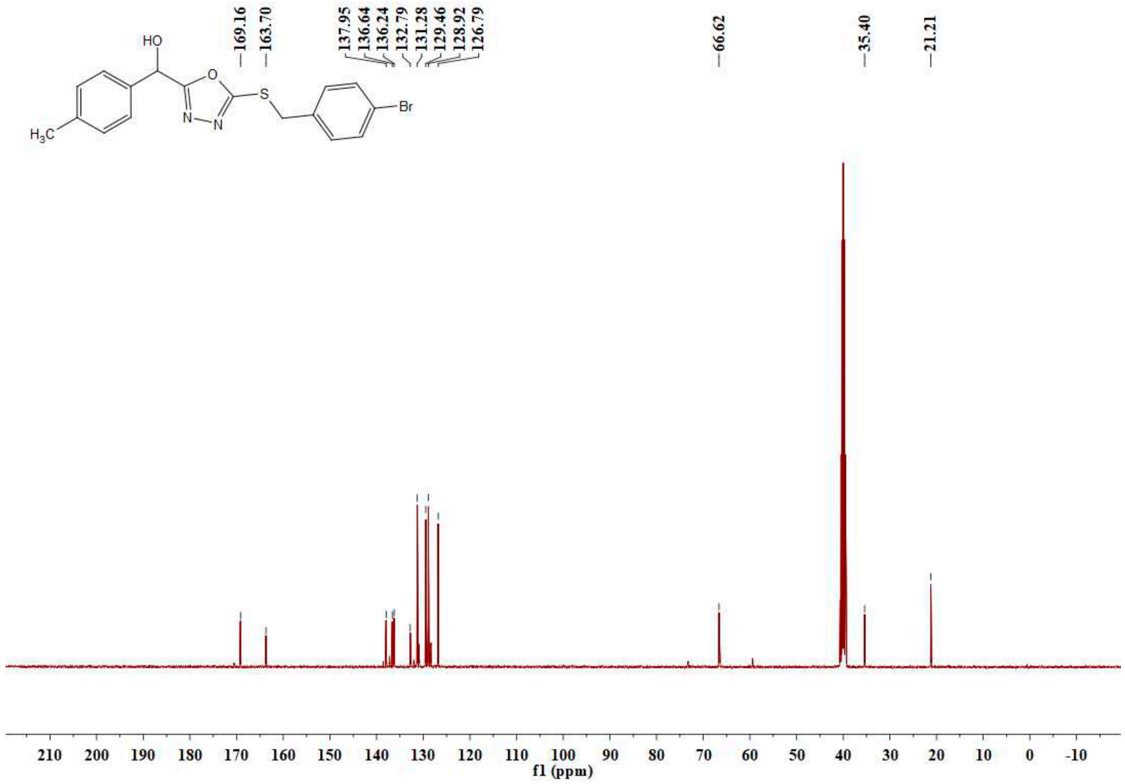

HO
O
N
N
S
Br
H_3C
169.16
163.70
137.95
136.64
136.24
132.79
131.28
129.46
128.92
126.79
66.62
35.40
21.21
210 200 190 180 170 160 150 140 130 120 110 100 90 80 70 60 50 40 30 20 10 0 -10
f1 (ppm)

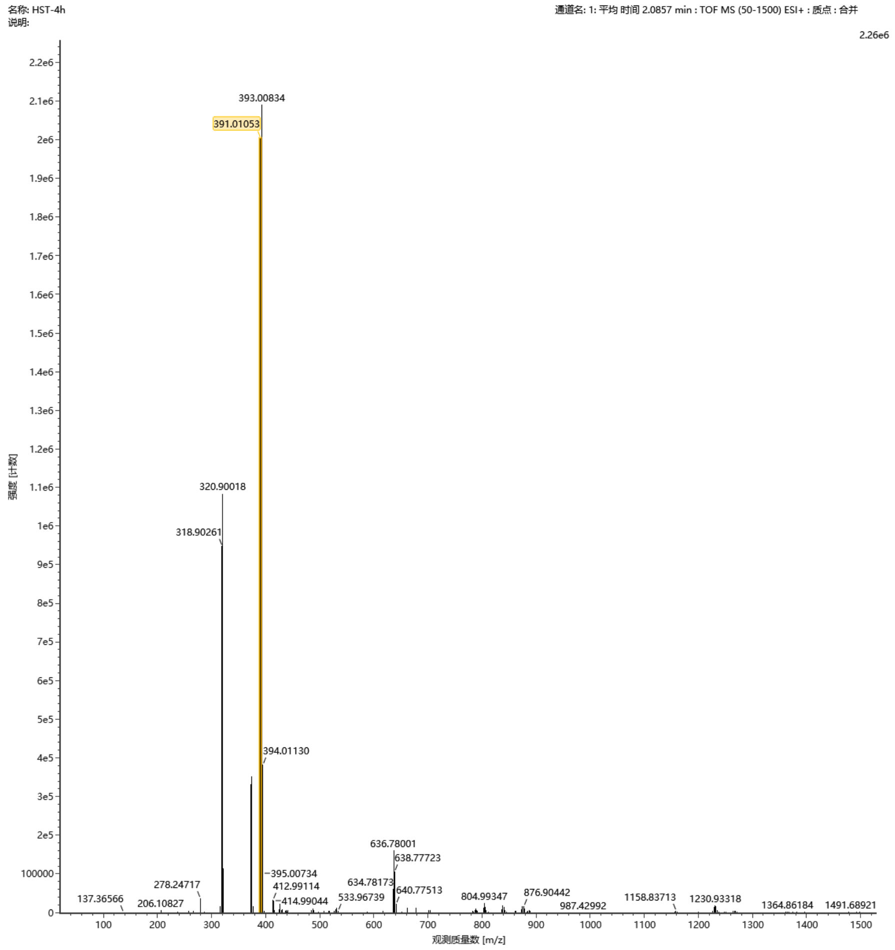

名称: HST-4h
说明:
通道名: 1: 平均 时间 2.0857 min : TOF MS (50-1500) ESI+ : 质点 : 合并
2.26e6
393.00834
391.01053
320.90018
318.90261
394.01130
395.00734
412.99114
414.99044
636.78001
638.77723
634.78173
640.77513
533.96739
137.36566
206.10827
278.24717
804.99347
876.90442
987.42992
1158.83713
1230.93318
1364.86184
1491.68921
强度 [计数]
观测质量数 [m/z]

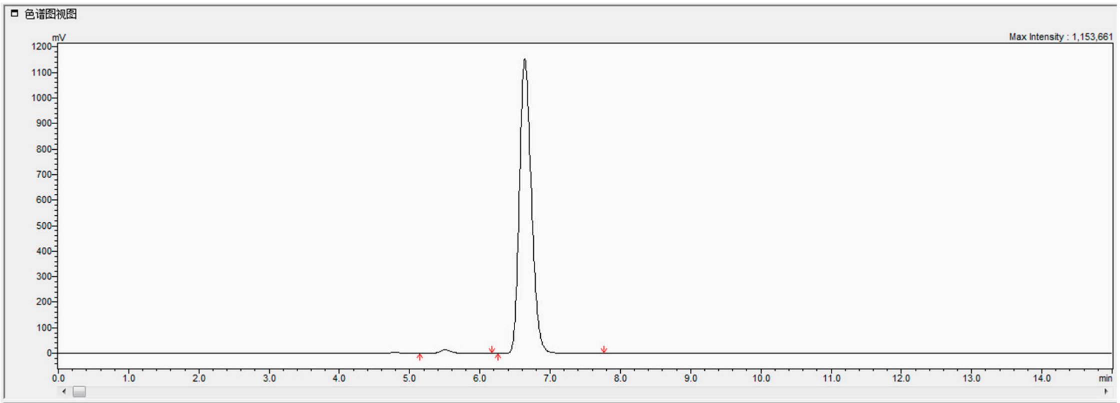

结果视图 - 峰表

峰表 | 化合物 | 组 | 校准曲线

峰号	保留时间	面积	峰宽 (高度50%)	分离度 (USP)	拖尾因	高度	面积%	理论塔板数 (USP)	拖尾因子
1	5.503	155179	0.162	--	1.191	13815	1.128	6061	1.766
2	6.641	13601353	0.182	3.795	1.164	1153416	98.872	7023	1.197
总计		13756532				1167231	100.000		

Figure S7. ^{1}H NMR, ^{13}C NMR, HRMS, and HPLC for **E**$_7$**.**

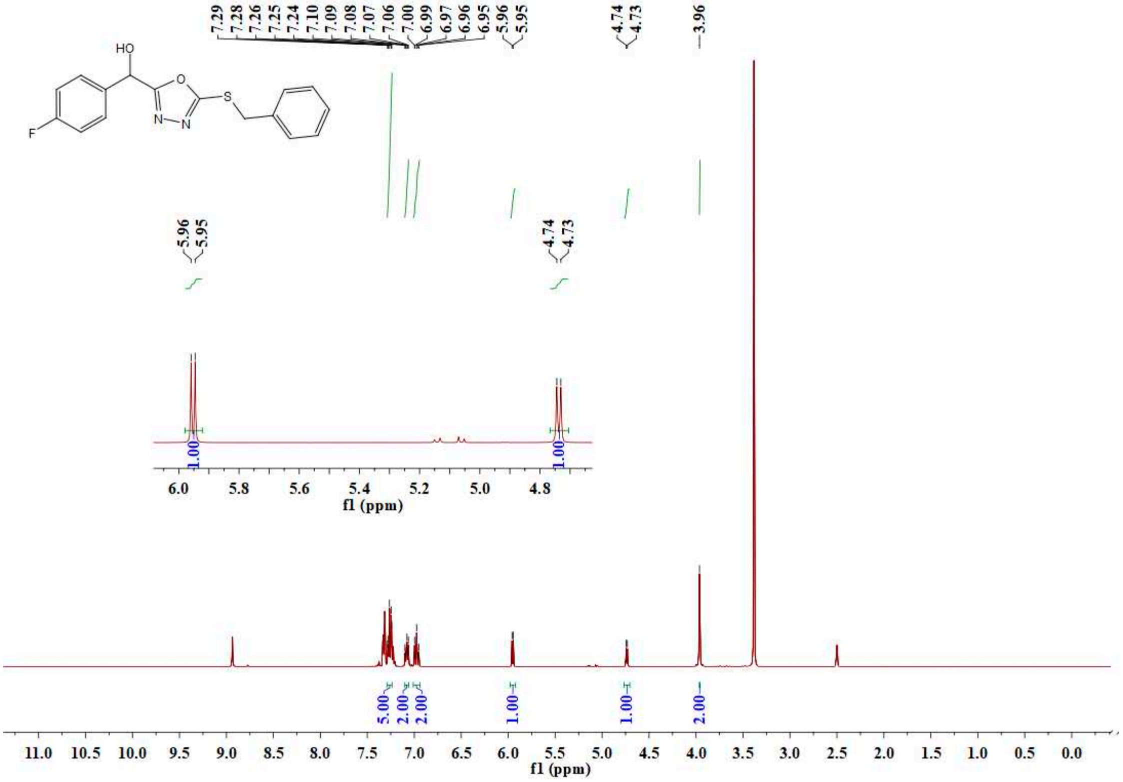

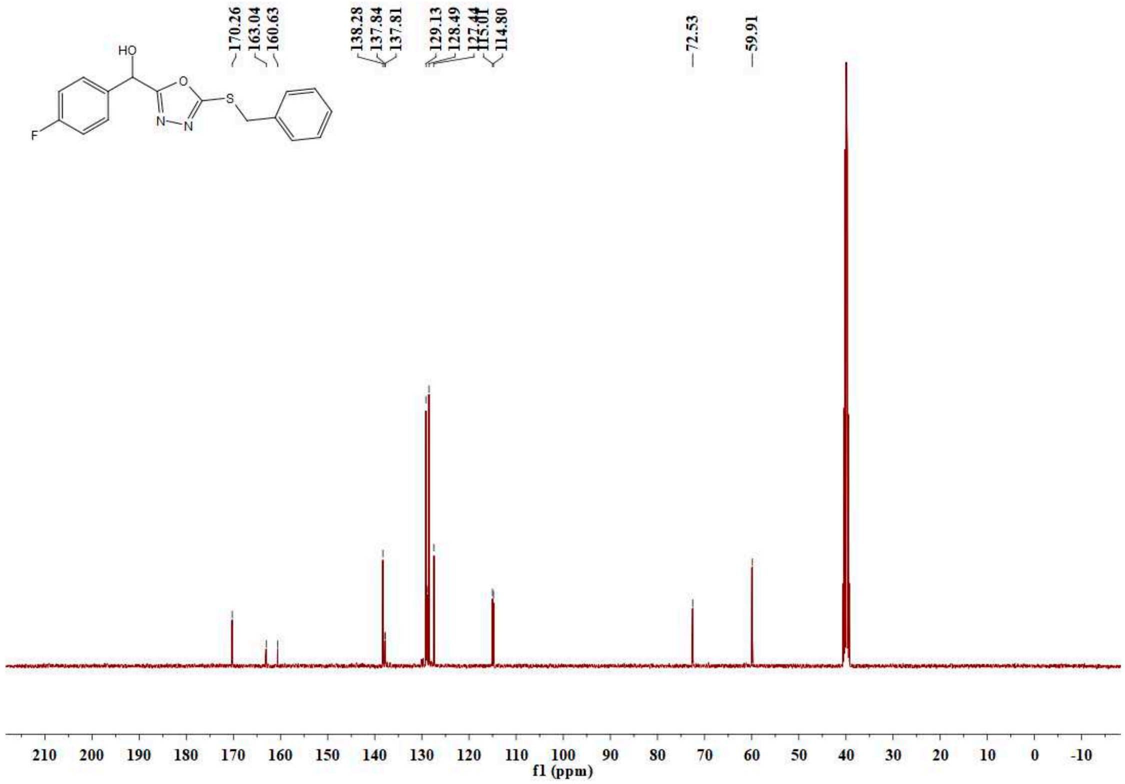
170.26
163.04
160.63
138.28
137.84
137.81
129.13
128.49
127.44
115.01
114.80
72.53
59.91
HO
O
N
N
S
F
210 200 190 180 170 160 150 140 130 120 110 100 90 80 70 60 50 40 30 20 10 0 -10
f1 (ppm)

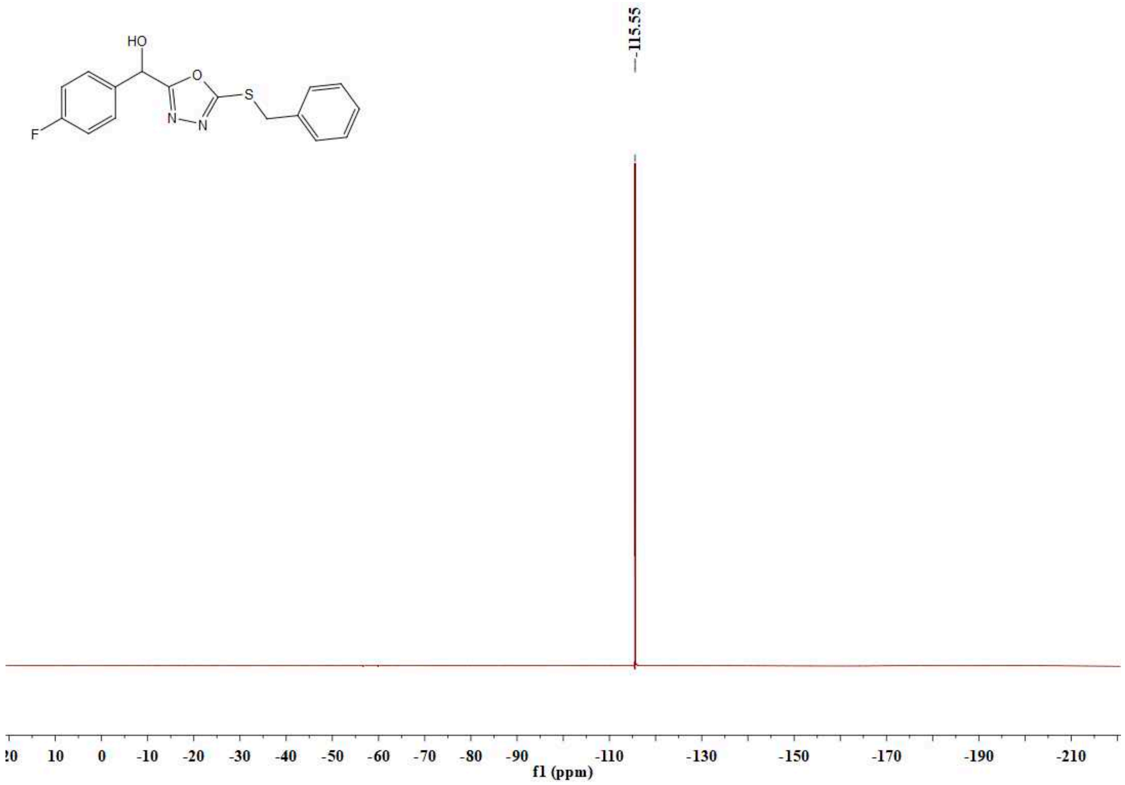
-115.55
HO
O
N
N
S
F
10 0 -10 -20 -30 -40 -50 -60 -70 -80 -90 -110 -130 -150 -170 -190 -210
f1 (ppm)

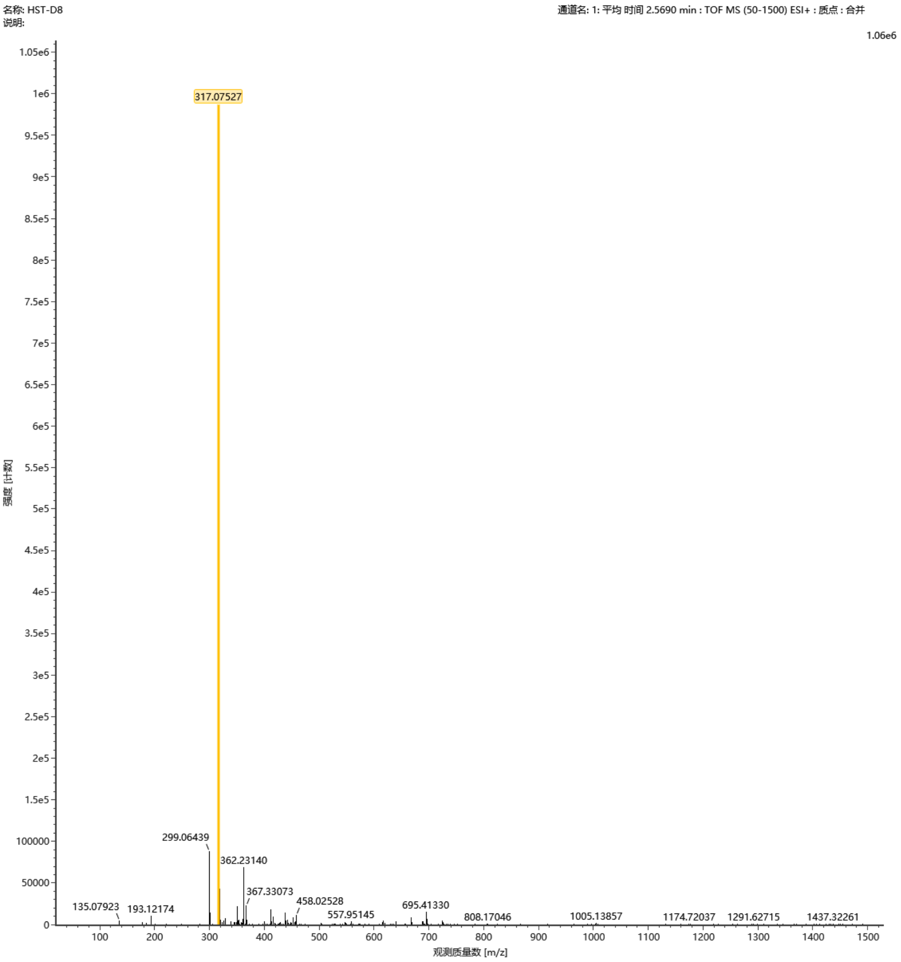

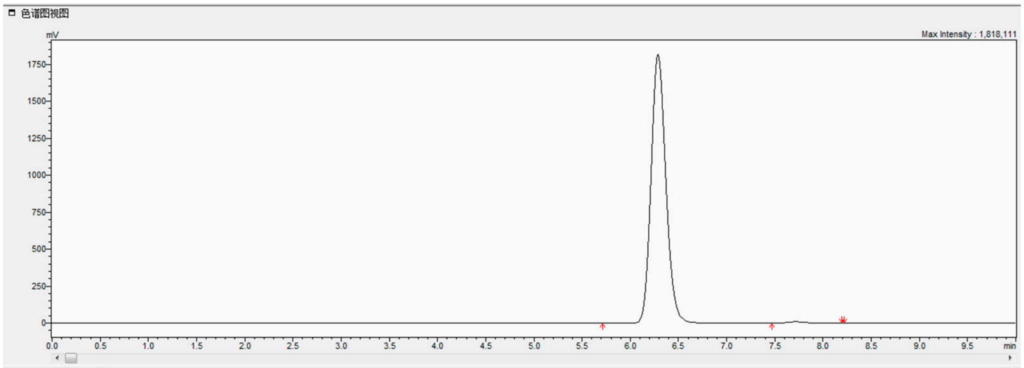

结果视图 - 峰表

峰表 | 化合物 | 组 | 校准曲线

峰号	保留时间	面积	峰宽 (高度50%)	分离度 (USP)	拖尾因	高度	面积%	理论塔板数 (USP)	拖尾因子
1	6.290	19560934	0.165	--	1.122	1817982	99.423	7530	1.152
2	7.715	113490	0.195	4.534	1.141	8992	0.577	8305	1.172
总计		19674424				1826974	100.000		

Figure S8. ^{1}H NMR, ^{13}C NMR, ^{19}F NMR, HRMS and HPLC for **E$_8$.**

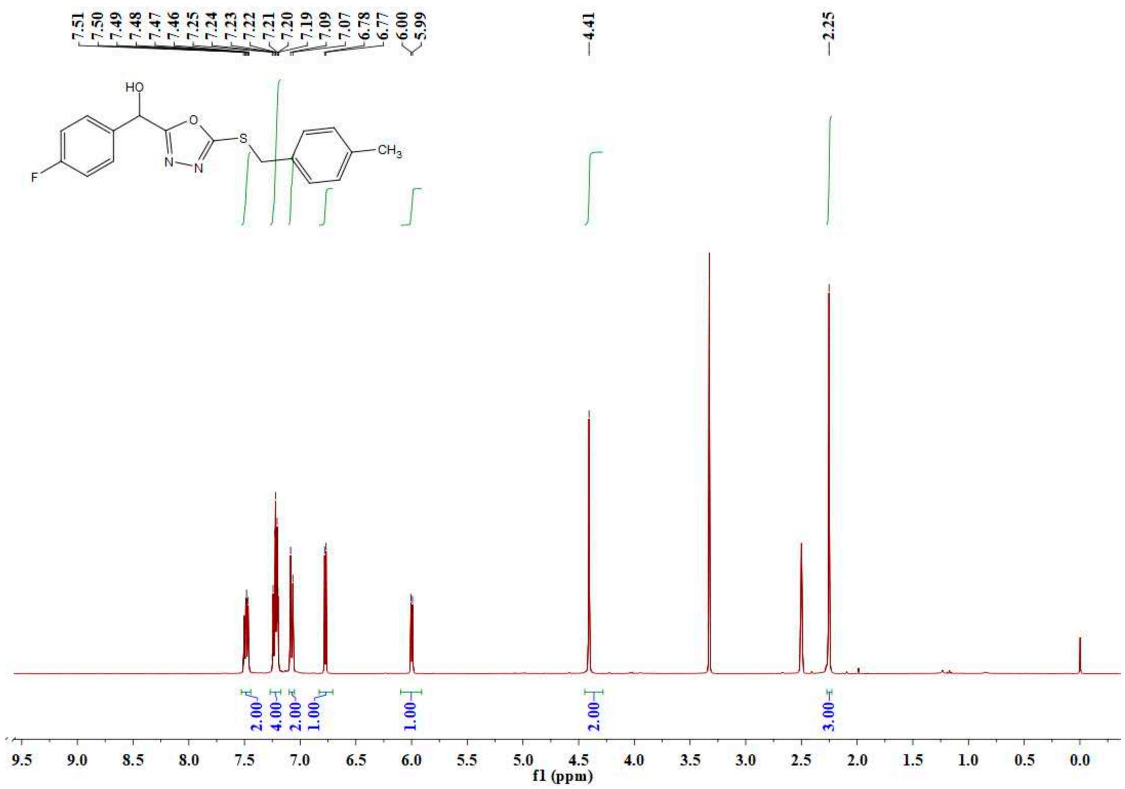

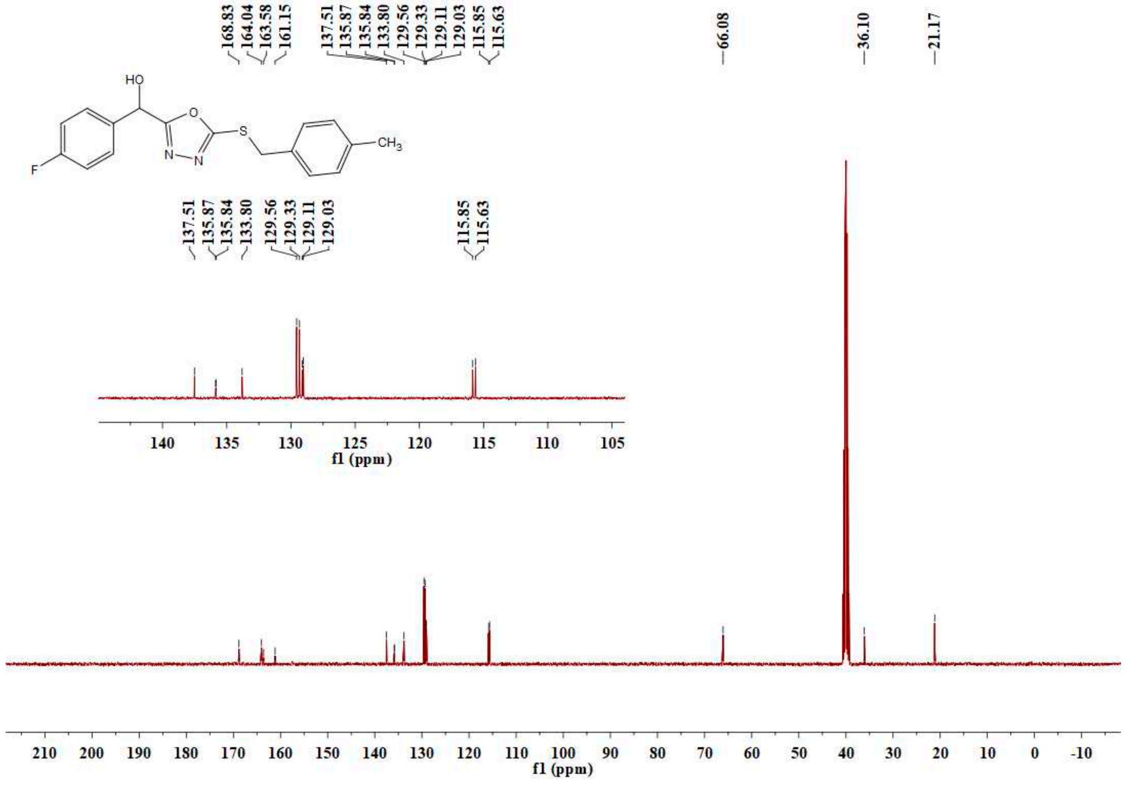

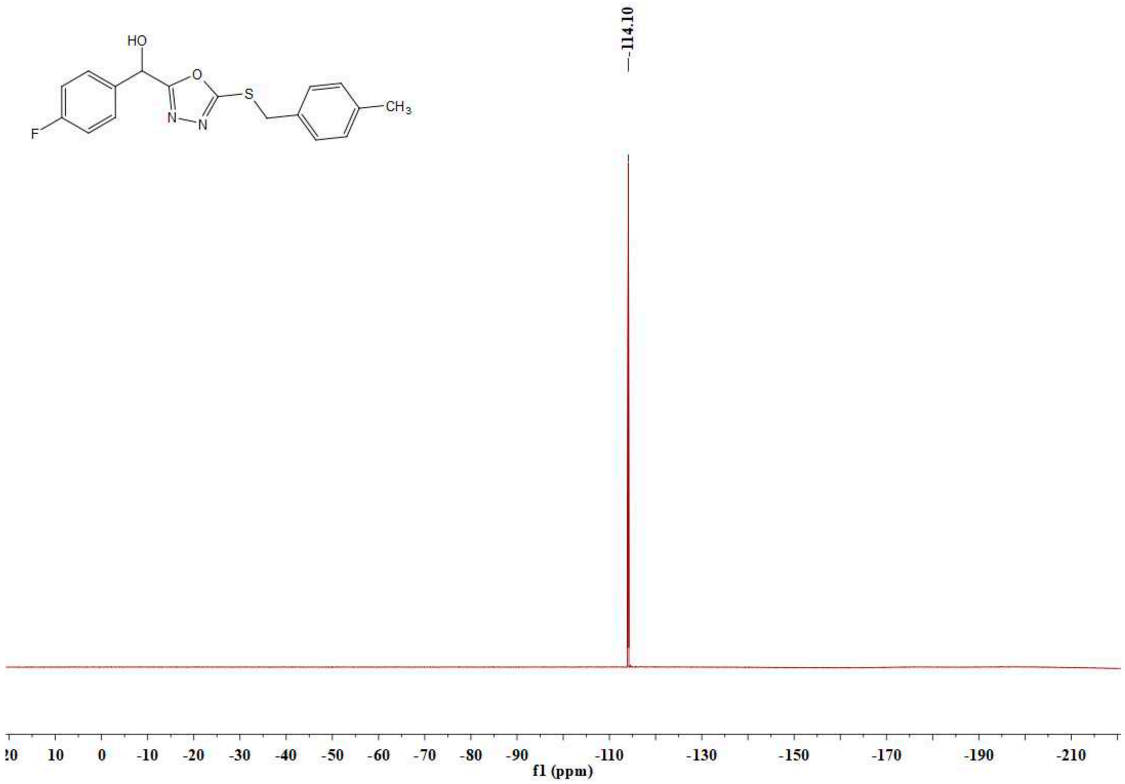
HO
O
S
CH3
F
N
N
-114.10
20
10
0
-10
-20
-30
-40
-50
-60
-70
-80
-90
-110
-130
-150
-170
-190
-210
f1 (ppm)

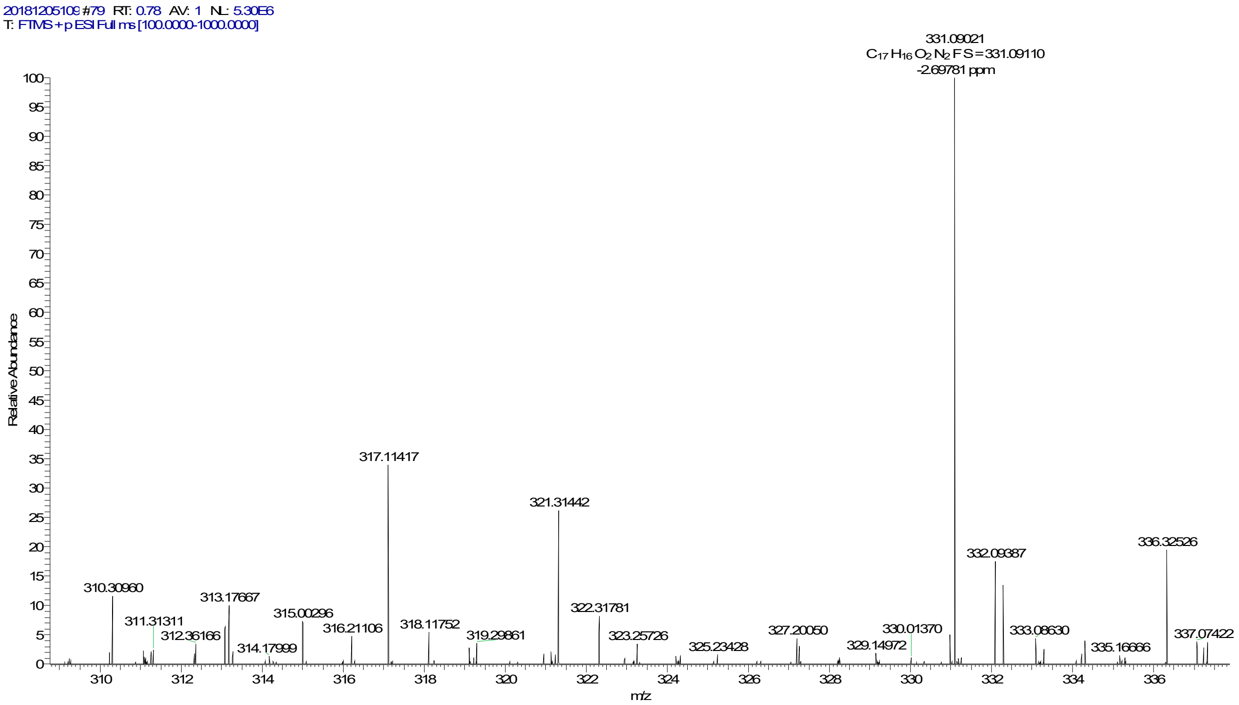
20181205109 #79 RT: 0.78 AV: 1 NL: 5.30E6
T: FTMS + p ESI Full ms [100.0000-1000.0000]
331.09021
C17 H16 O2 N2 F S = 331.09110
-2.69781 ppm
317.11417
321.31442
336.32526
332.09387
310.30960
313.17667
322.31781
315.00296
311.31311
312.36166
316.21106
318.11752
319.29861
323.25726
327.20050
330.01370
333.08630
337.07422
314.17999
325.23428
329.14972
335.16666
Relative Abundance
m/z

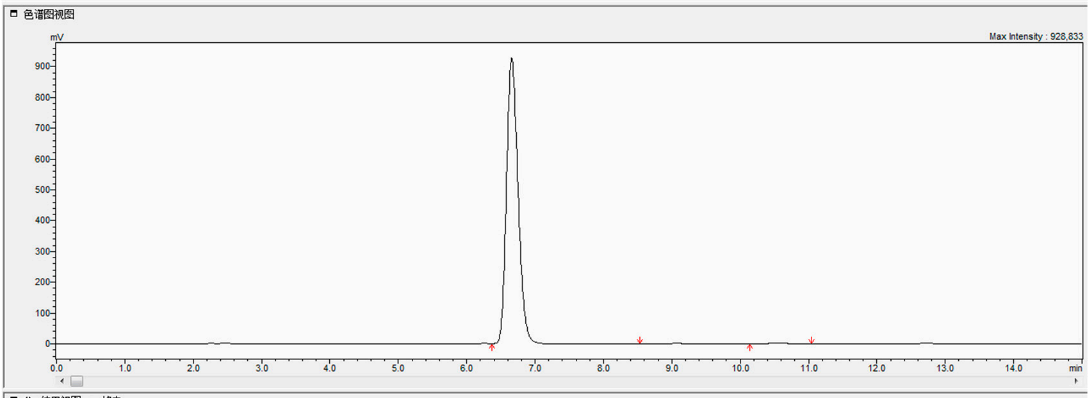

峰号	保留时间	面积	峰宽 (高度50%)	分离度 (USP)	拖尾因	高度	面积%	理论塔板数 (USP)	拖尾因子
1	6.659	10864875	0.180	--	1.172	928978	99.464	7228	1.206
2	10.543	58598	0.268	10.034	1.124	3335	0.536	8378	1.127
总计		10923473				932313	100.000		

Figure S9. ^{1}H NMR, ^{13}C NMR, ^{19}F NMR, HRMS and HPLC for **E9.**

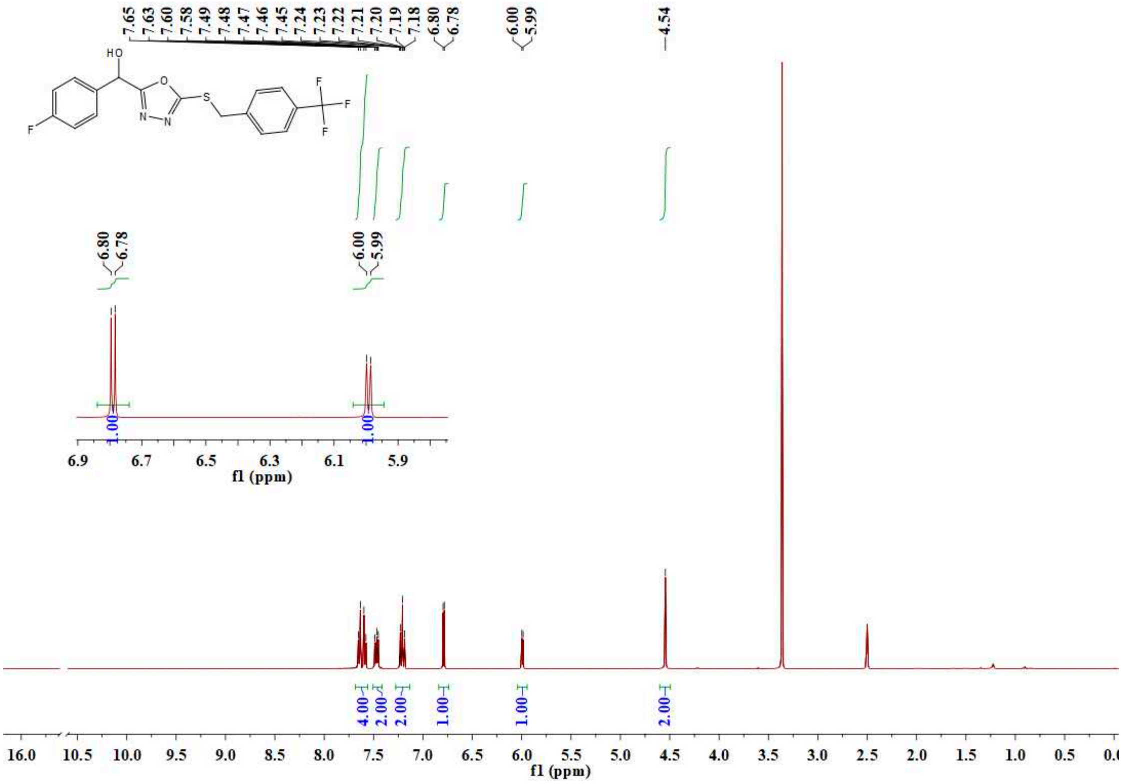

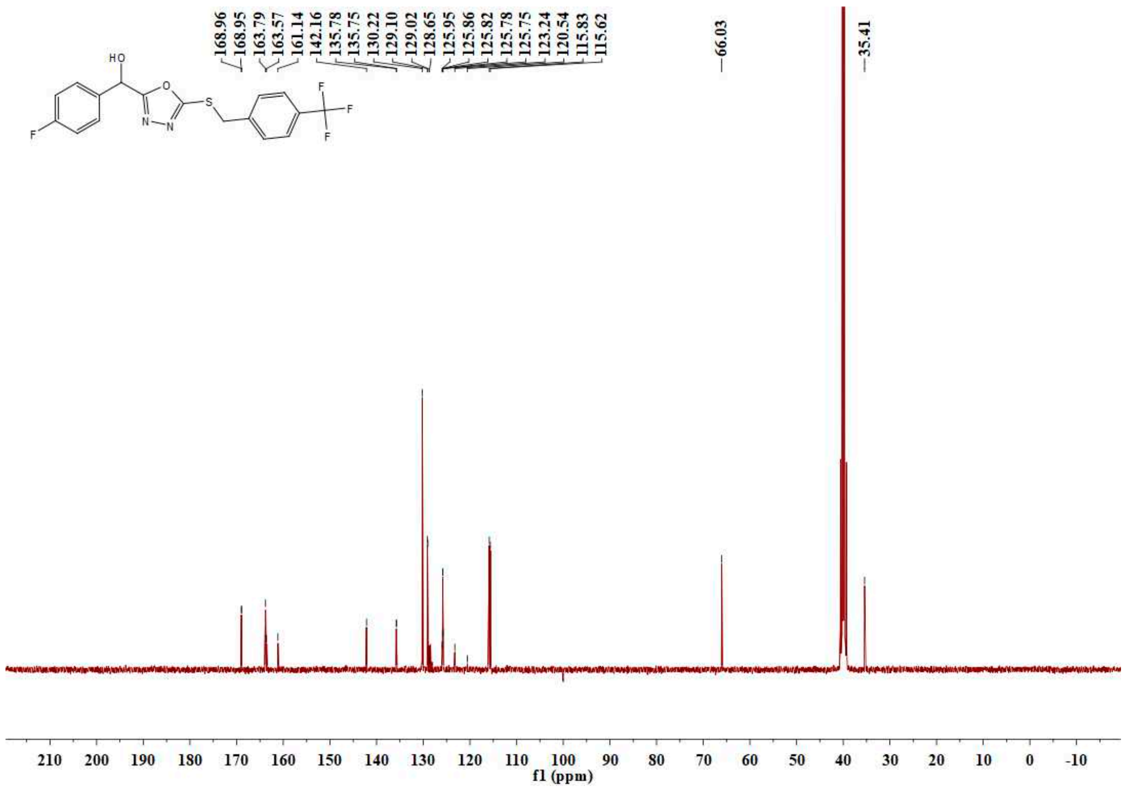
168.96
168.95
163.79
163.57
161.14
142.16
135.78
135.75
130.22
129.10
129.02
128.65
125.95
125.86
125.82
125.78
125.75
123.24
120.54
115.83
115.62
66.03
35.41
HO
O
S
N
F
210 200 190 180 170 160 150 140 130 120 110 100 90 80 70 60 50 40 30 20 10 0 -10
f1 (ppm)

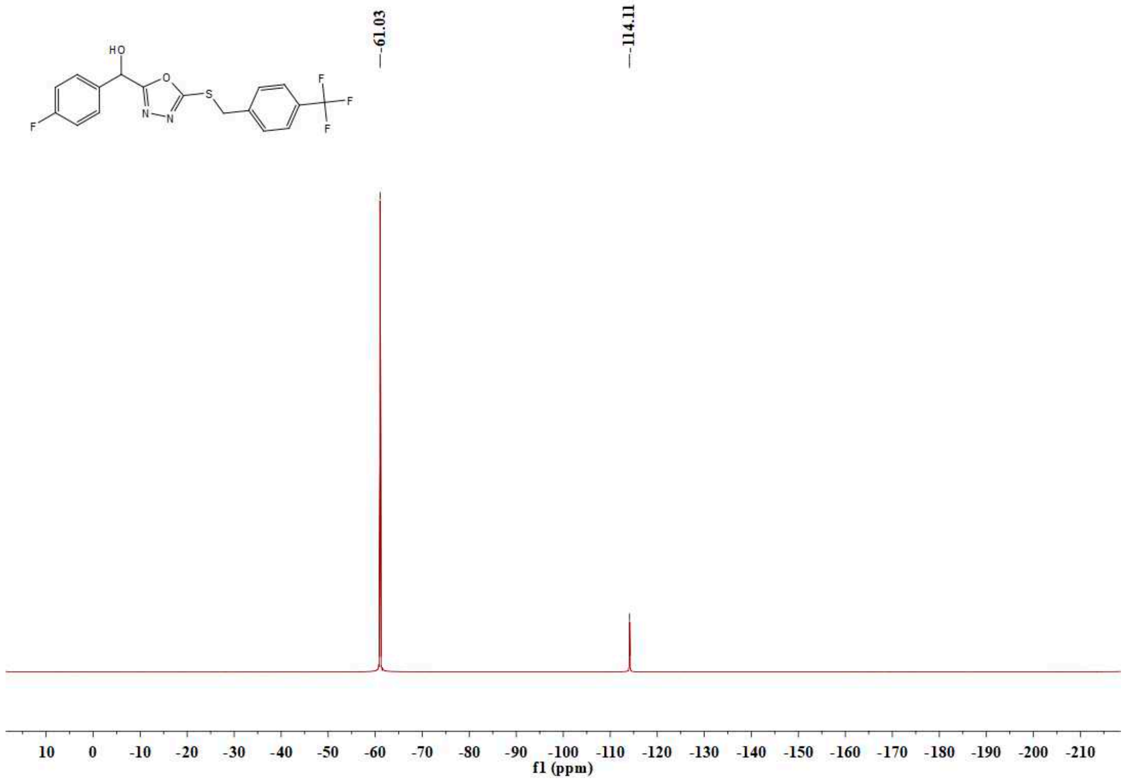
-61.03
-114.11
HO
O
S
N
F
10 0 -10 -20 -30 -40 -50 -60 -70 -80 -90 -100 -110 -120 -130 -140 -150 -160 -170 -180 -190 -200 -210
f1 (ppm)

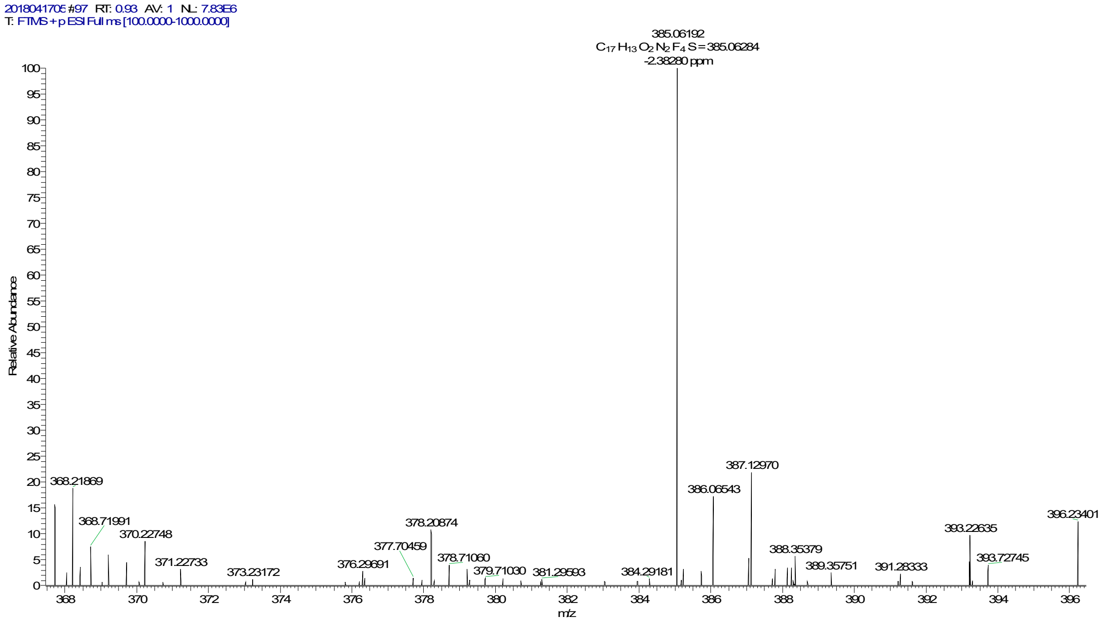

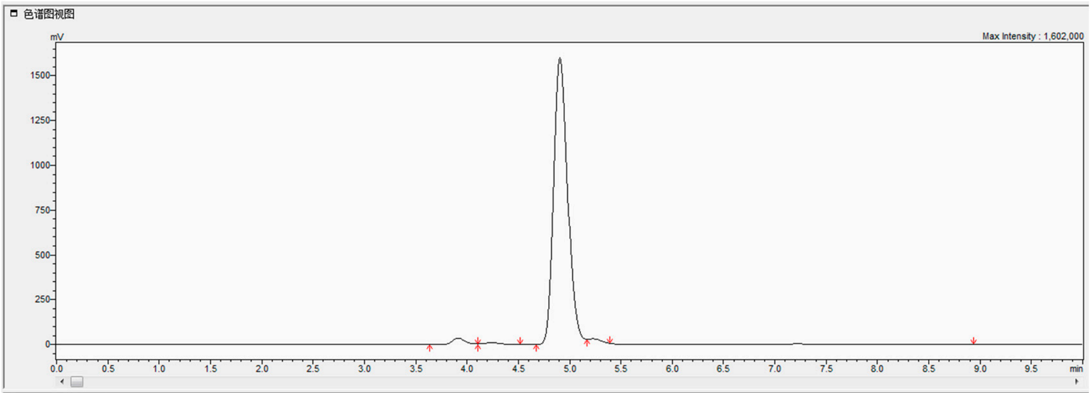

结果视图 - 峰表

峰表 | 化合物 | 组 | 校准曲线

峰号	保留时间	面积	峰宽 (高度50%)	分离度 (USP)	拖尾因	高度	面积%	理论塔板数 (USP)	拖尾因子
1	3.913	318922	0.132	--	1.270	35630	2.011	4422	--
2	4.239	103579	0.148	1.294	0.000	10087	0.653	3974	--
3	4.909	15371315	0.145	2.550	1.162	1601968	96.910	5861	1.195
4	5.229	67639	0.117	1.441	1.690	9994	0.426	12415	1.692
总计		15861454				1657679	100.000		

Figure S10. ^{1}H NMR, ^{13}C NMR, ^{19}F NMR, HRMS and HPLC for **E10.**

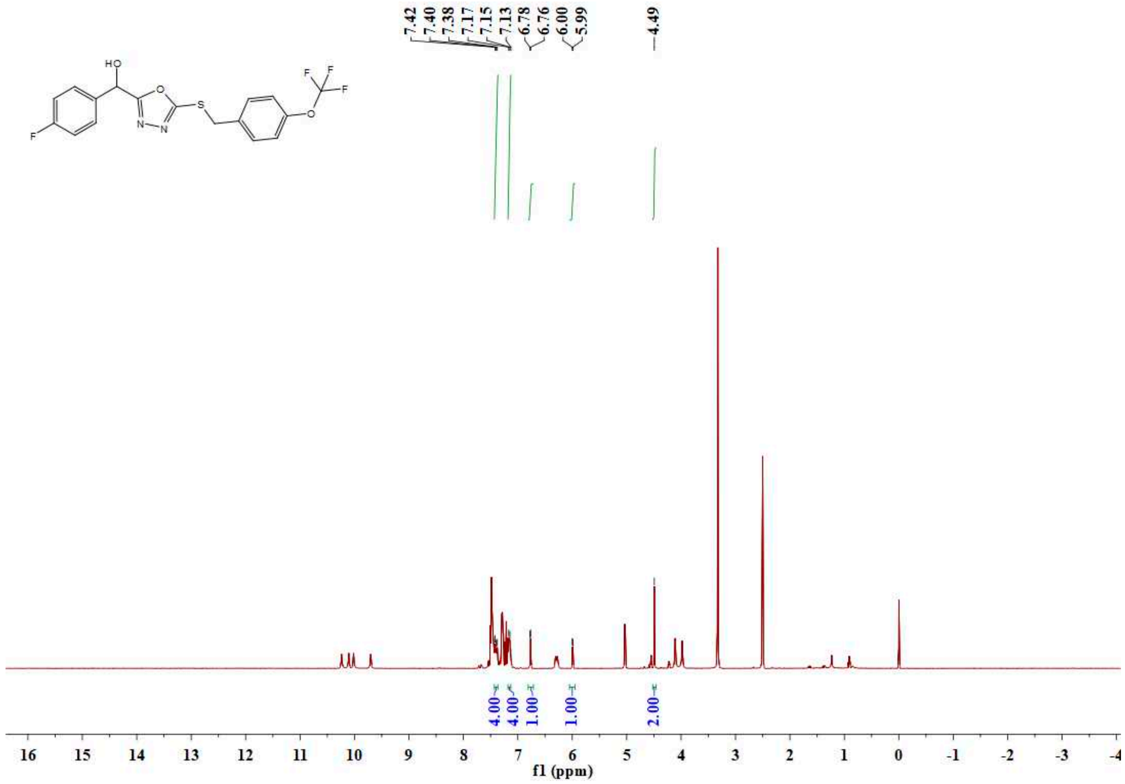
7.42
7.40
7.38
7.17
7.15
7.13
6.78
6.76
6.00
5.99
4.49
4.00
4.00
1.00
1.00
2.00
16 15 14 13 12 11 10 9 8 7 6 5 4 3 2 1 0 -1 -2 -3 -4
f1 (ppm)

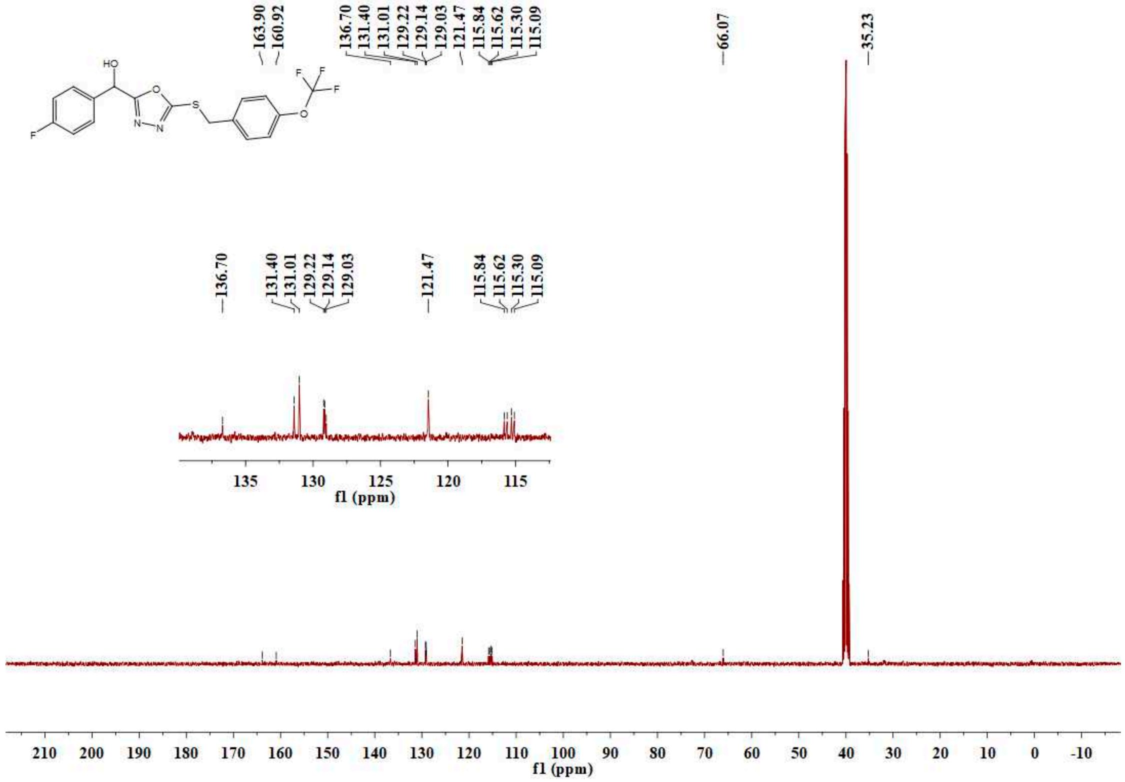
163.90
160.92
136.70
131.40
131.01
129.22
129.14
129.03
121.47
115.84
115.62
115.30
115.09
66.07
35.23
135 130 125 120 115
f1 (ppm)
210 200 190 180 170 160 150 140 130 120 110 100 90 80 70 60 50 40 30 20 10 0 -10
f1 (ppm)

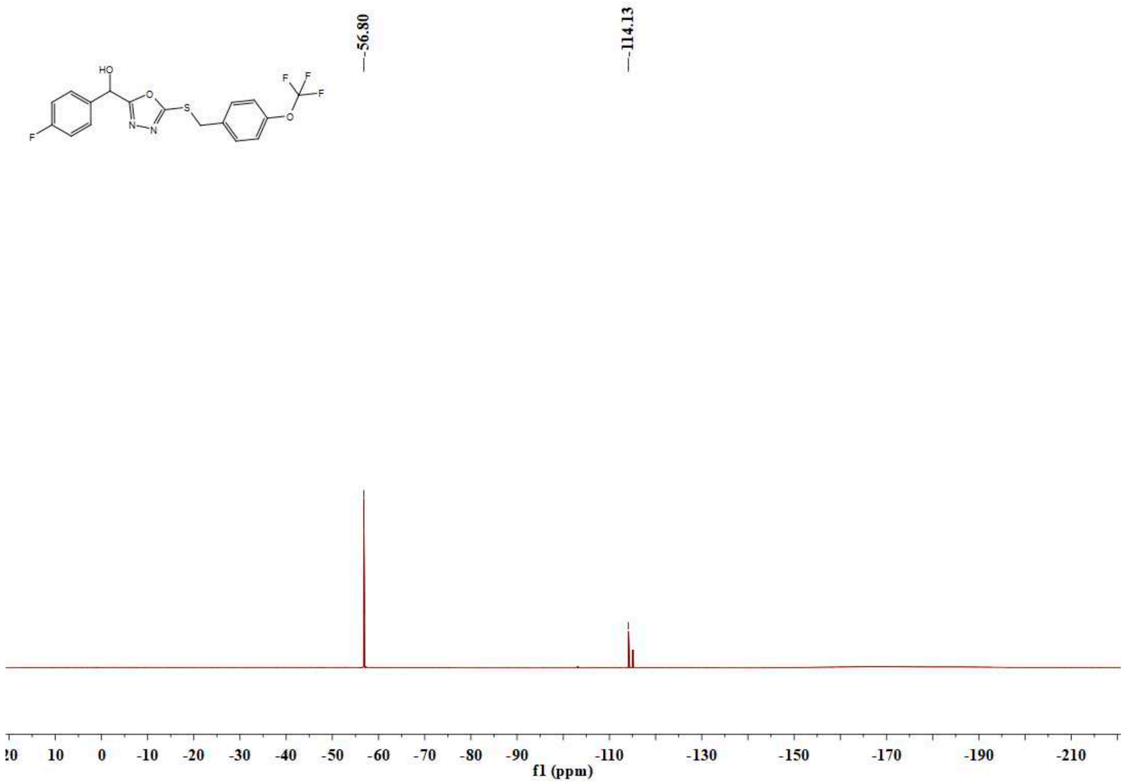

—56.80
—114.13
HO
F
O
N
N
S
O
F
F
F
20 10 0 -10 -20 -30 -40 -50 -60 -70 -80 -90 -110 -130 -150 -170 -190 -210
f1 (ppm)

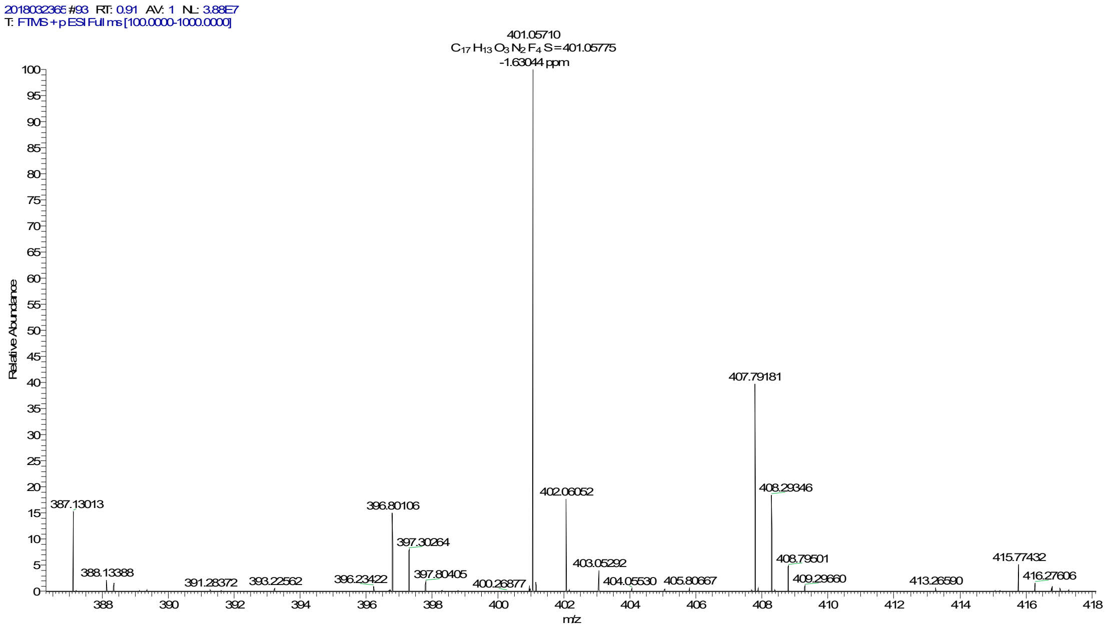

2018032365 #93 RT: 0.91 AV: 1 NL: 3.88E7
T: FTMS + p ESI Full ms [100.0000-1000.0000]
401.05710
C17 H13 O3 N2 F4 S = 401.05775
-1.63044 ppm
Relative Abundance
407.79181
402.06052
408.29346
387.13013
396.80106
397.30264
403.05292
408.79501
415.77432
388.13388
391.28372
393.22562
396.23422
397.80405
400.26877
404.05530
405.80667
409.29660
413.26590
416.27606
m/z

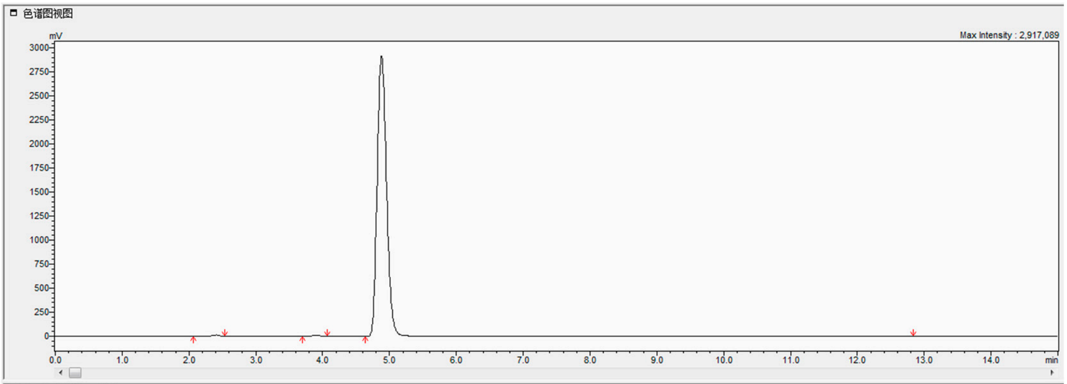

峰号	保留时间	面积	峰宽 (高度50%)	分离度 (USP)	拖尾因	高度	面积%	理论塔板数 (USP)	拖尾因子
1	2.412	120102	0.109	--	0.000	13887	0.418	2176	--
2	3.900	85396	0.151	6.230	0.000	8245	0.297	3325	--
3	4.879	28499674	0.151	3.692	1.173	2917930	99.284	5619	1.212
总计		28705172				2940062	100.000		

Figure S11. ^{1}H NMR, ^{13}C NMR, ^{19}F NMR, HRMS and HPLC for **E$_{11}$.**

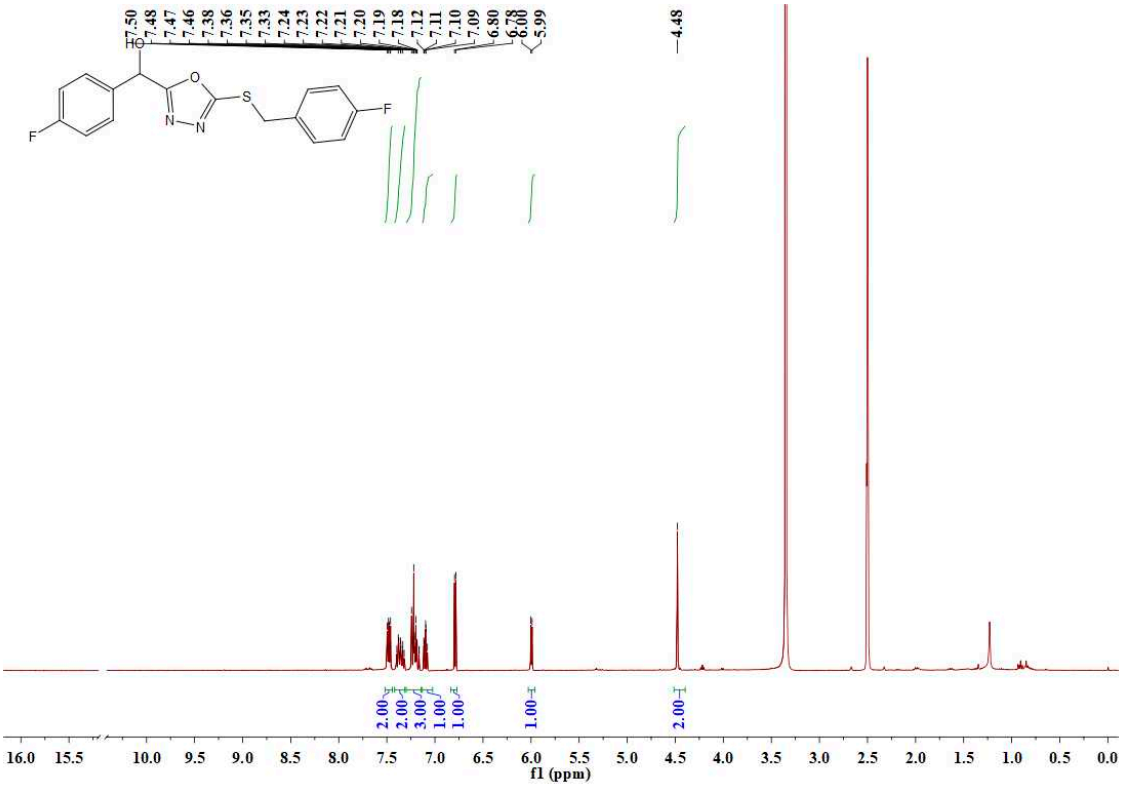

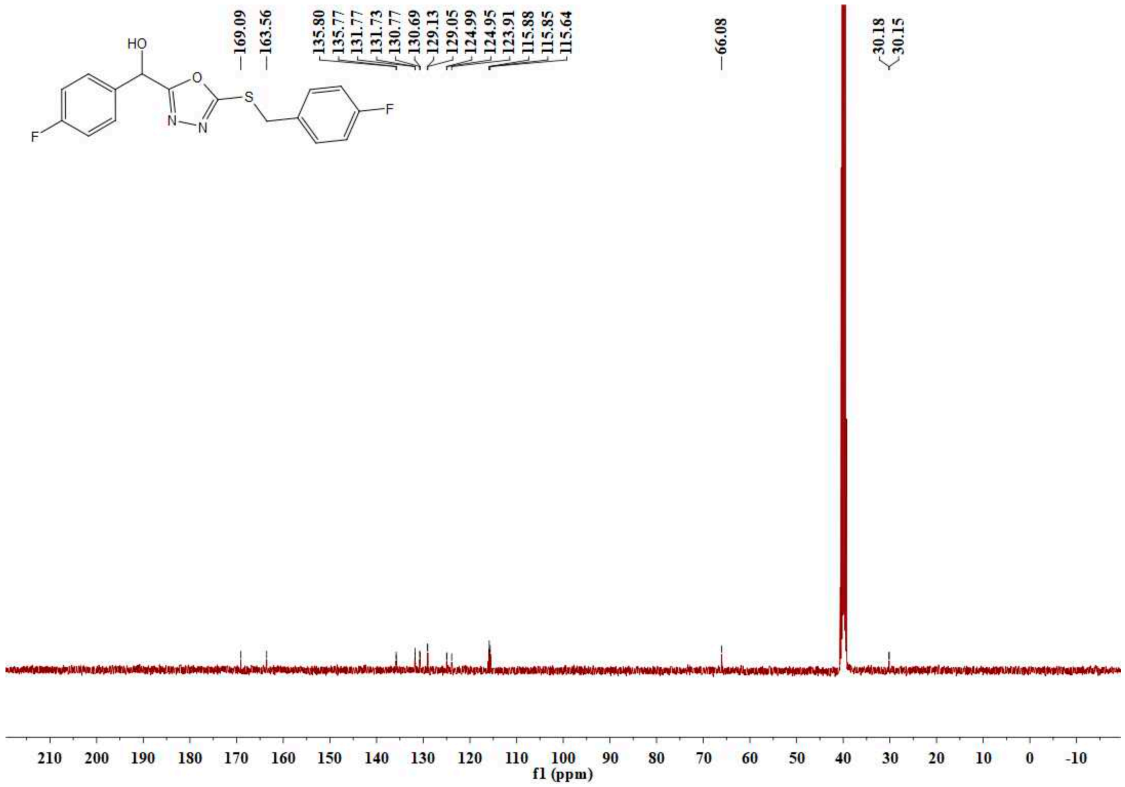
HO
O
N
N
S
F
F
169.09
163.56
135.80
135.77
131.77
131.73
130.77
130.69
129.13
129.05
124.99
124.95
123.91
115.88
115.85
115.64
66.08
30.18
30.15
210 200 190 180 170 160 150 140 130 120 110 100 90 80 70 60 50 40 30 20 10 0 -10
f1 (ppm)

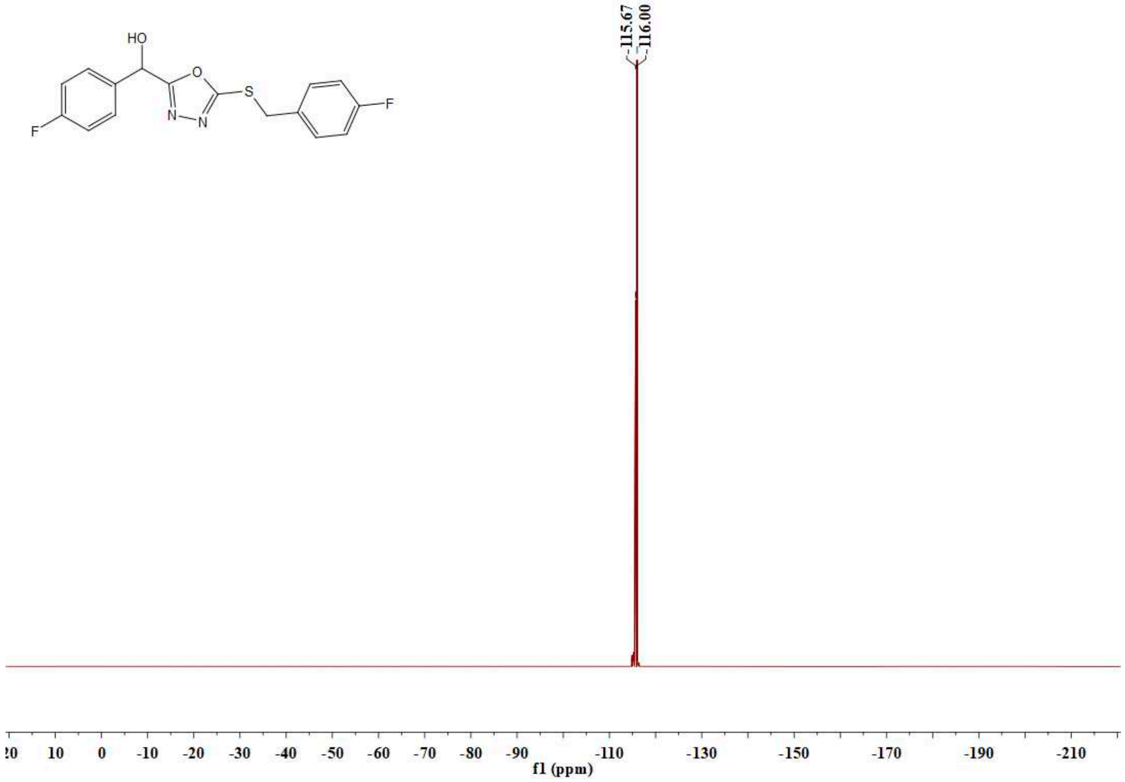
HO
O
N
N
S
F
F
-115.67
-116.00
10 0 -10 -20 -30 -40 -50 -60 -70 -80 -90 -110 -130 -150 -170 -190 -210
f1 (ppm)

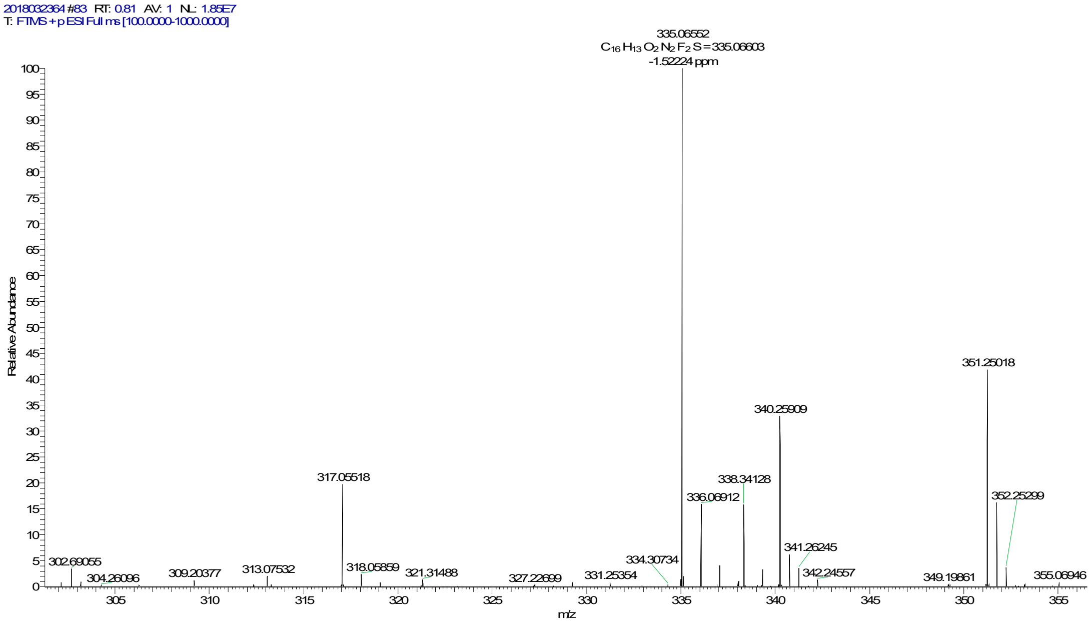

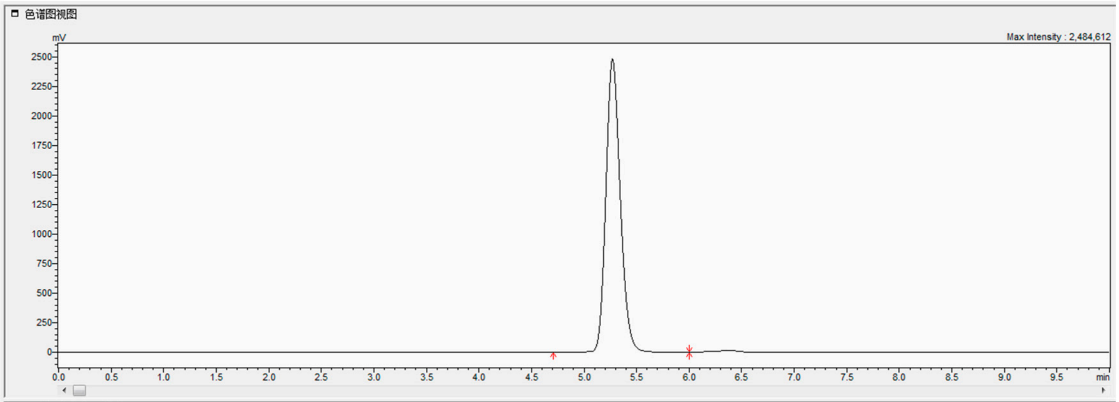

结果视图 - 峰表

峰表 | 化合物 | 组 | 校准曲线

峰号	保留时间	面积	峰宽 (高度50%)	分离度 (USP)	拖尾因	高度	面积%	理论塔板数 (USP)	拖尾因子
1	5.270	23868276	0.147	--	1.152	2484594	98.827	6529	1.184
2	6.377	283366	0.273	3.392	0.862	16765	1.173	4238	0.901
总计		24151641				2501358	100.000		

Figure S12. ^{1}H NMR, ^{13}C NMR, ^{19}F NMR, HRMS and HPLC for **E12.**

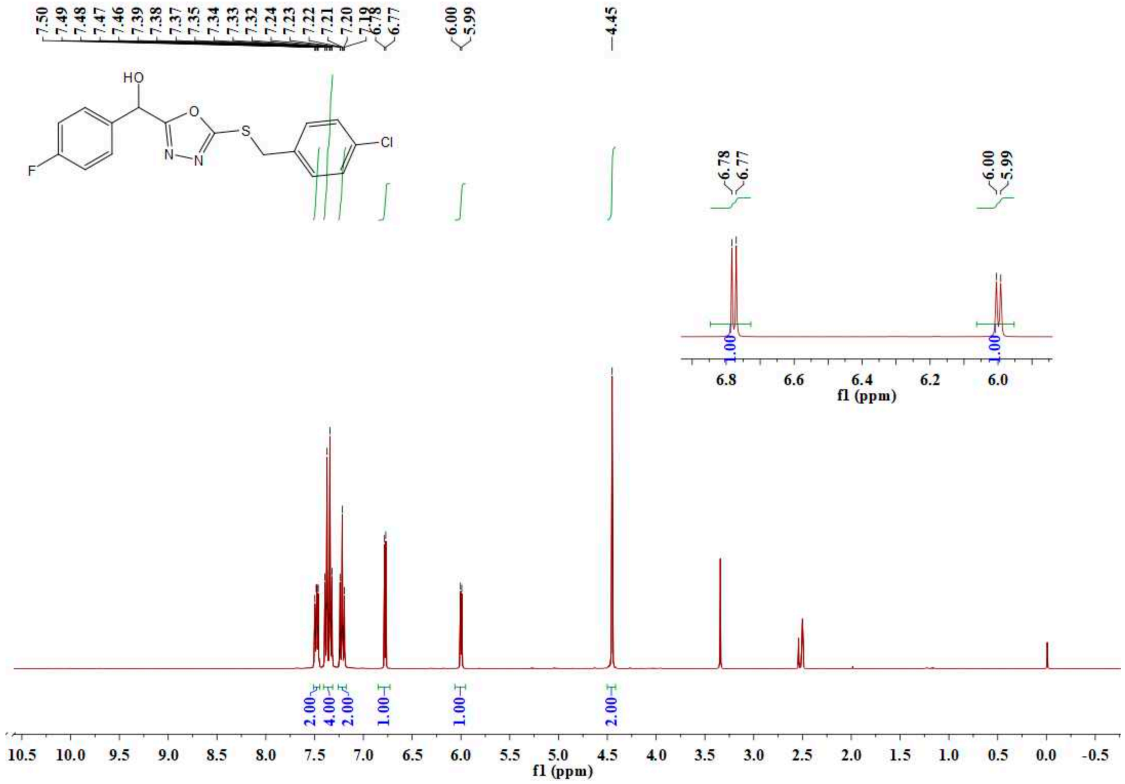
7.50
7.49
7.48
7.47
7.46
7.39
7.38
7.37
7.35
7.34
7.33
7.32
7.24
7.23
7.22
7.21
7.20
7.19
6.78
6.77
6.00
5.99
4.45
HO
O
S
Cl
F
N
N
2.00
4.00
2.00
1.00
1.00
2.00
f1 (ppm)

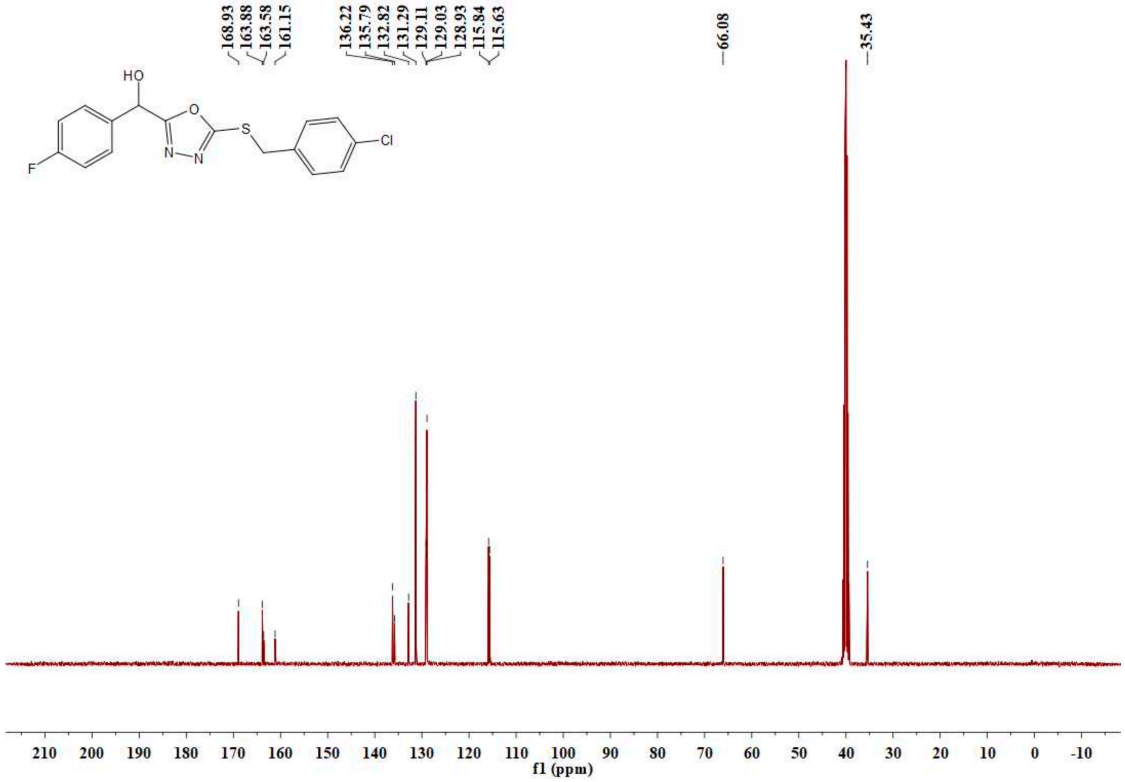
168.93
163.88
163.58
161.15
136.22
135.79
132.82
131.29
129.11
129.03
128.93
115.84
115.63
66.08
35.43
HO
O
S
Cl
F
N
N
f1 (ppm)

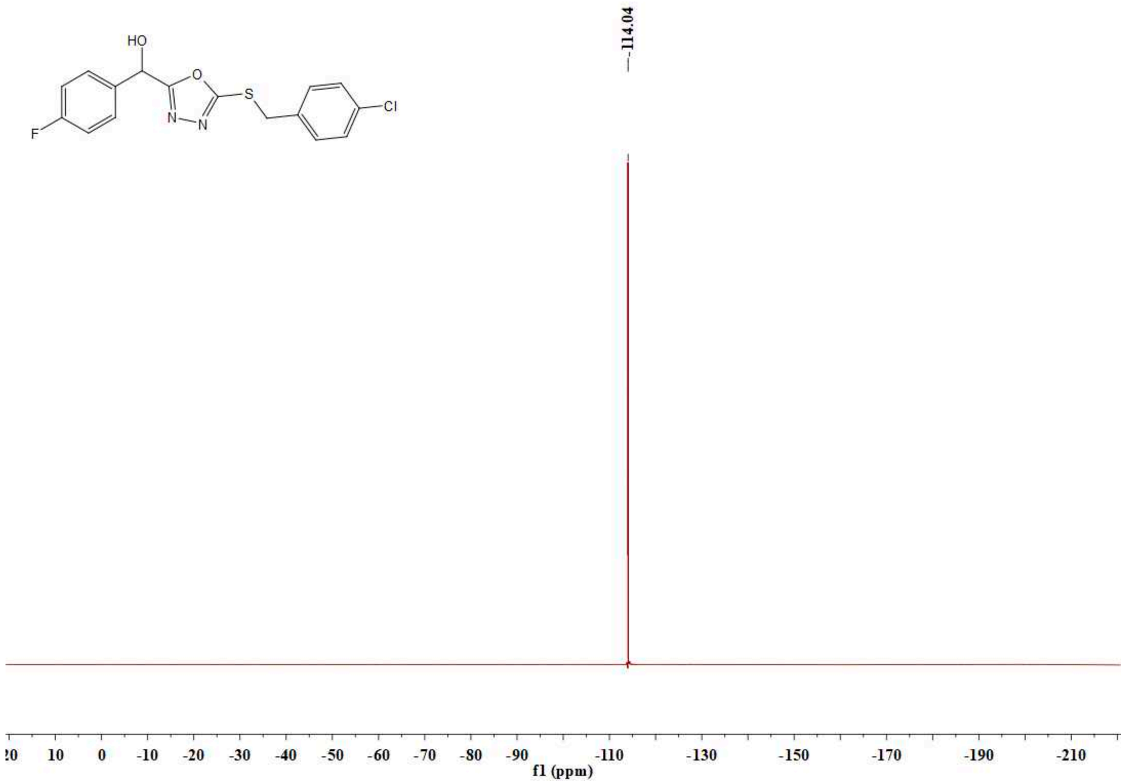
HO
O
S
Cl
F
N
N
-114.04
20 10 0 -10 -20 -30 -40 -50 -60 -70 -80 -90 -110 -130 -150 -170 -190 -210
f1 (ppm)

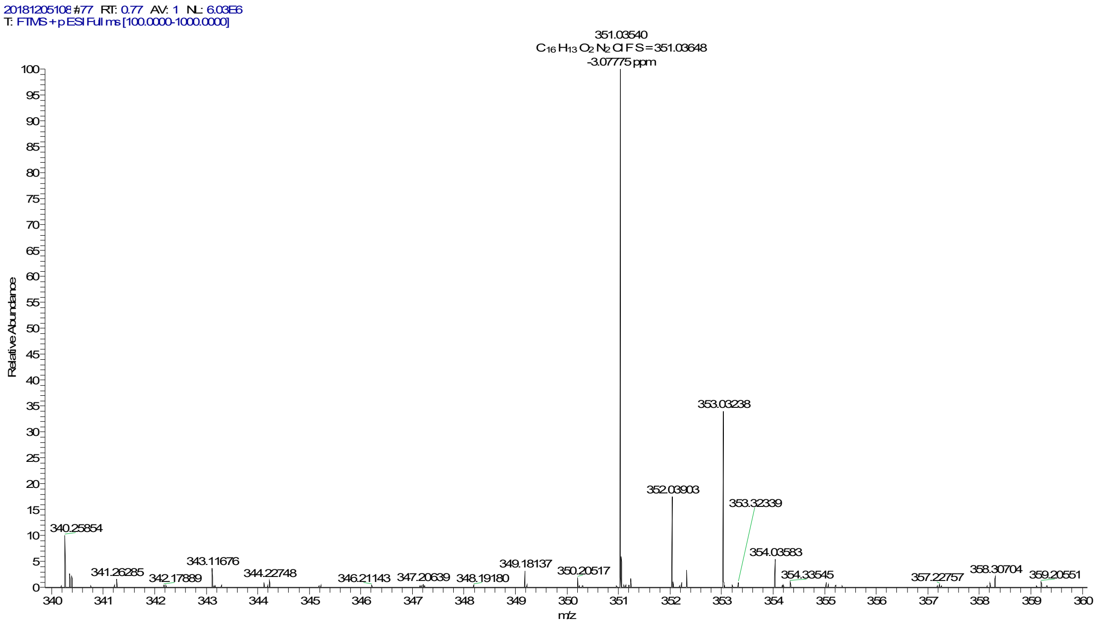
20181205108 #77 RT: 0.77 AV: 1 NL: 6.03E6
T: FTMS + p ESI Full ms [100.0000-1000.0000]
351.03540
C16 H13 O2 N2 Cl F S = 351.03648
-3.07775 ppm
353.03238
352.03903
353.32339
340.25854
354.03583
343.11676
349.18137
341.26285
342.17889
344.22748
346.21143
347.20639
348.19180
350.20517
354.33545
357.22757
358.30704
359.20551
Relative Abundance
m/z

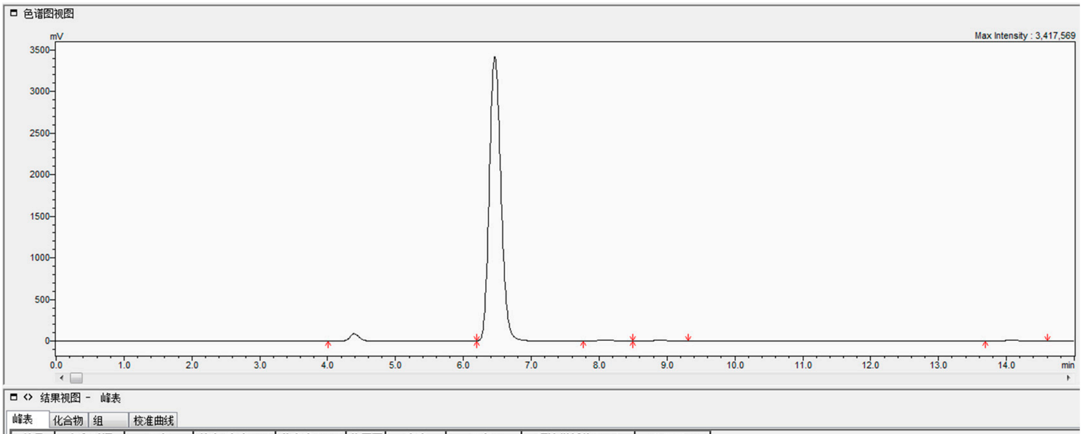

峰号	保留时间	面积	峰宽 (高度50%)	分离度 (USP)	拖尾因	高度	面积%	理论塔板数 (USP)	拖尾因子
1	4.391	952417	0.144	--	1.145	87532	2.245	4763	1.137
2	6.462	40957814	0.186	7.251	1.135	3417628	96.553	6667	1.167
3	8.082	207216	0.212	4.730	1.232	14869	0.488	7694	1.280
4	8.898	145340	0.248	2.040	1.036	8717	0.343	6798	1.028
5	14.078	157372	0.313	10.672	1.169	7642	0.371	10915	1.200
总计		42420158				3536389	100.000		

Figure S13. ^{1}H NMR, ^{13}C NMR, ^{19}F NMR, HRMS and HPLC for **E13**.

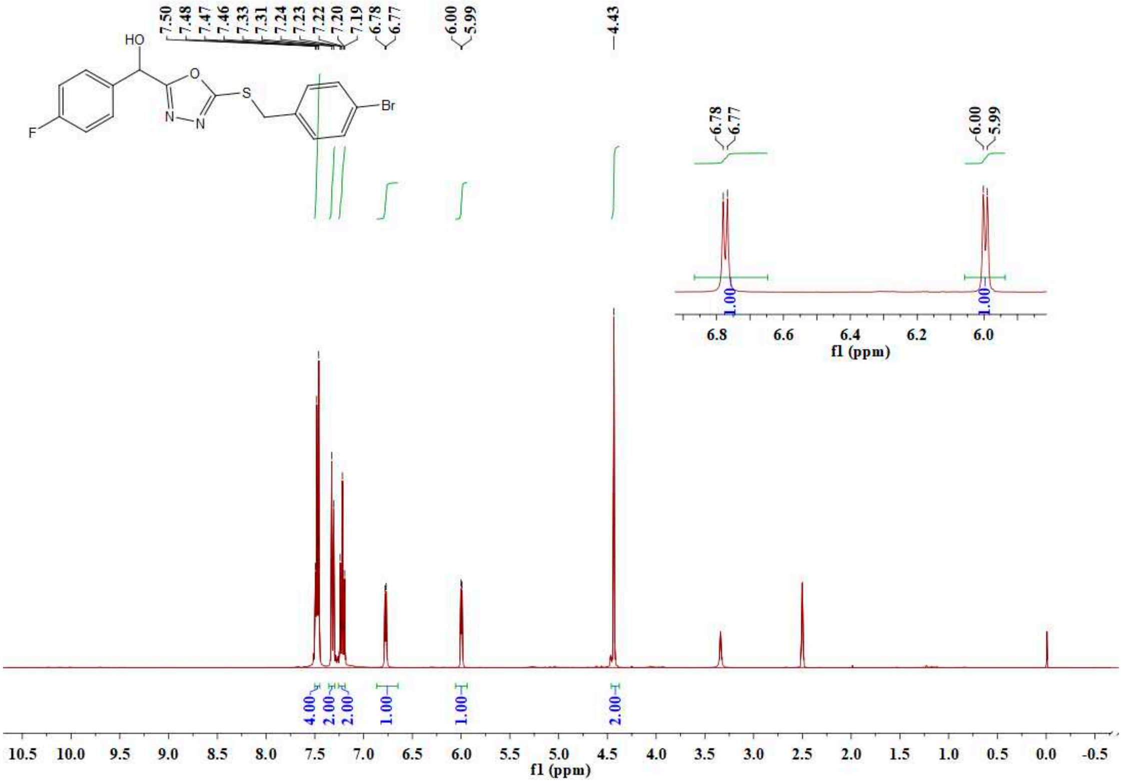

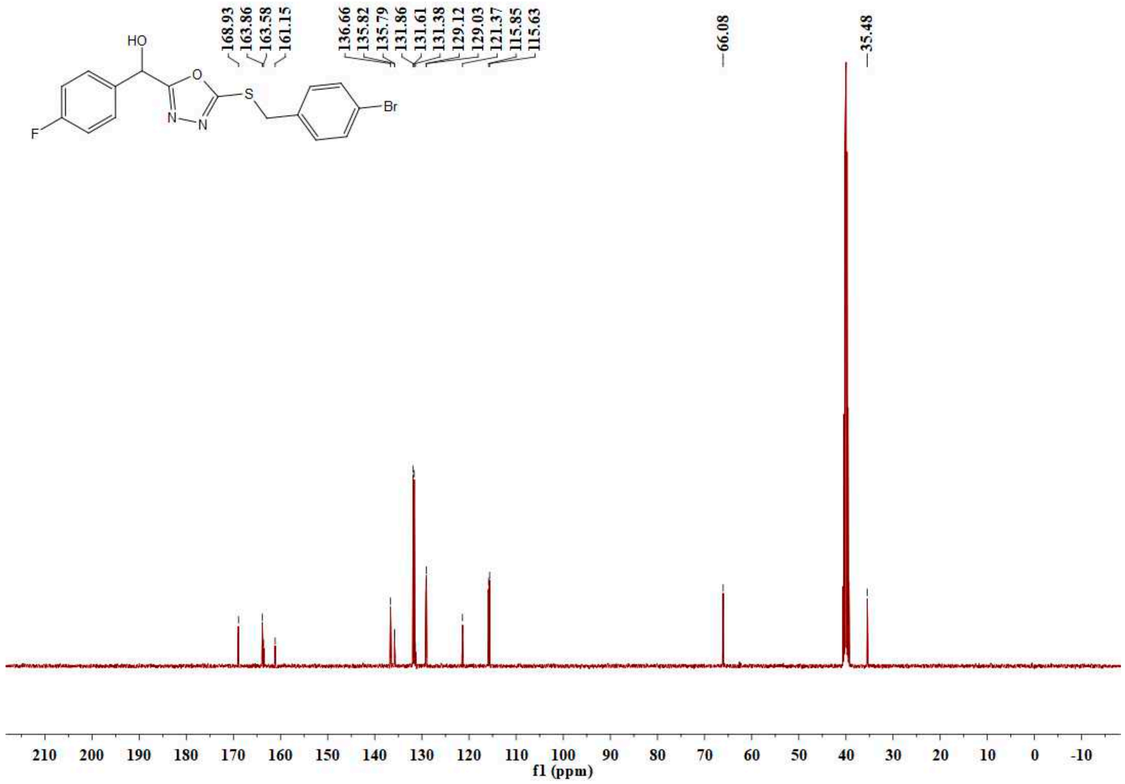
HO
O
S
Br
F
N
N
168.93
163.86
163.58
161.15
136.66
135.82
135.79
131.86
131.61
131.38
129.12
129.03
121.37
115.85
115.63
66.08
35.48
210 200 190 180 170 160 150 140 130 120 110 100 90 80 70 60 50 40 30 20 10 0 -10
f1 (ppm)

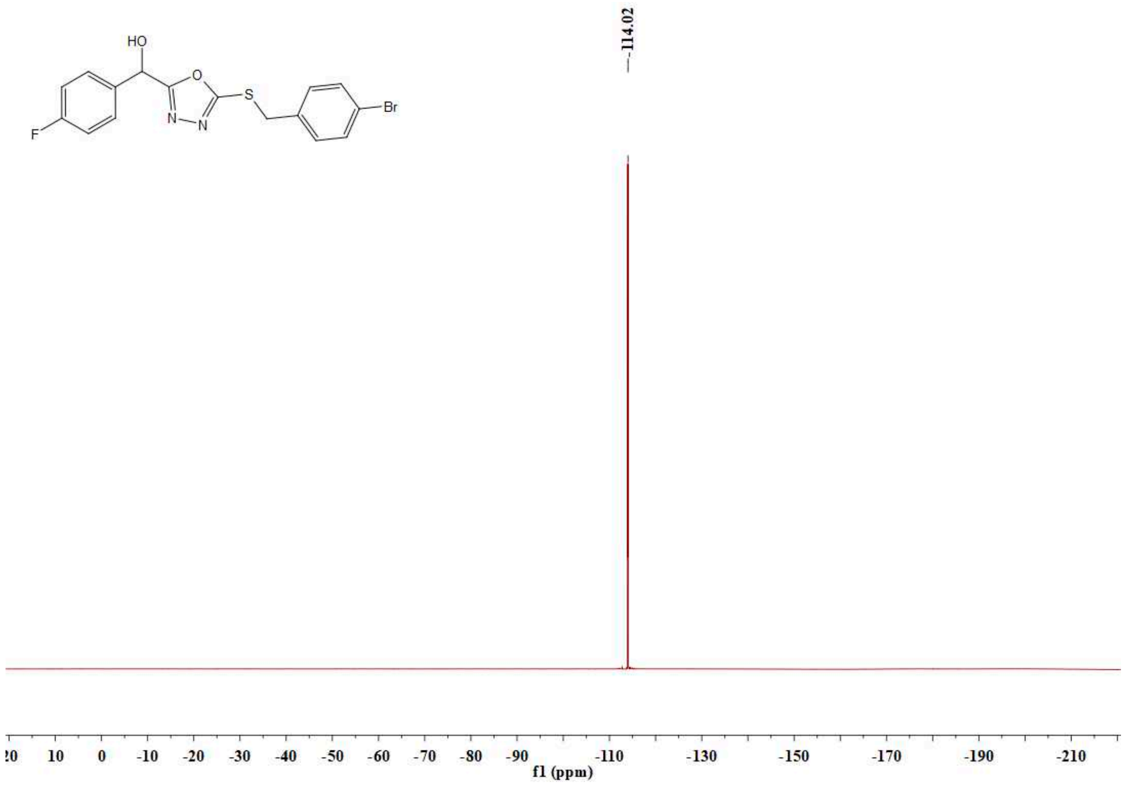
HO
O
S
Br
F
N
N
-114.02
20 10 0 -10 -20 -30 -40 -50 -60 -70 -80 -90 -110 -130 -150 -170 -190 -210
f1 (ppm)

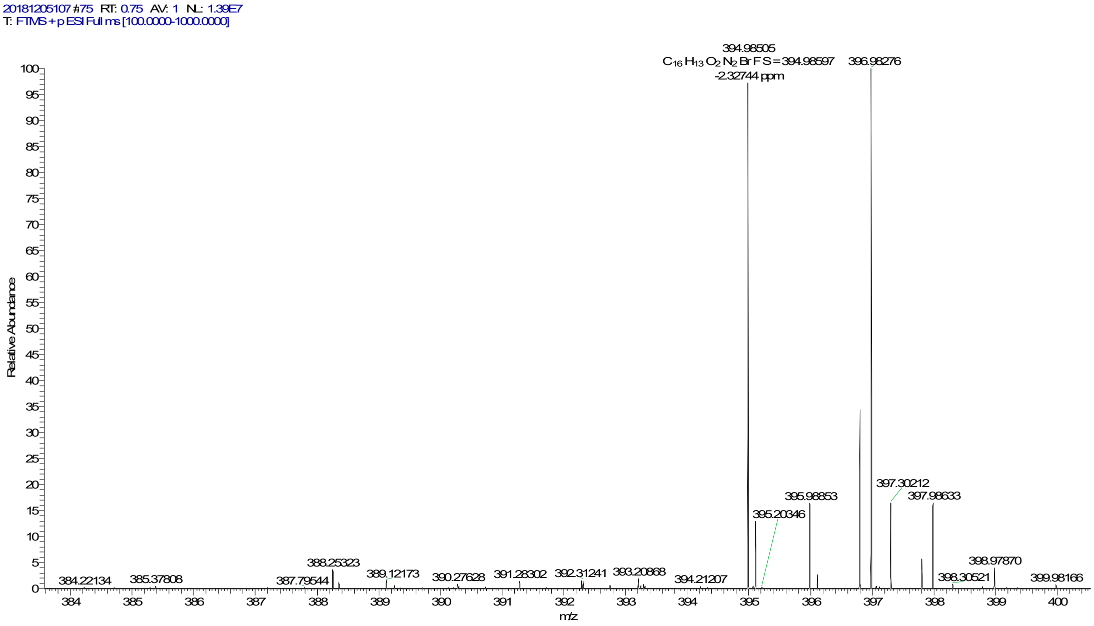

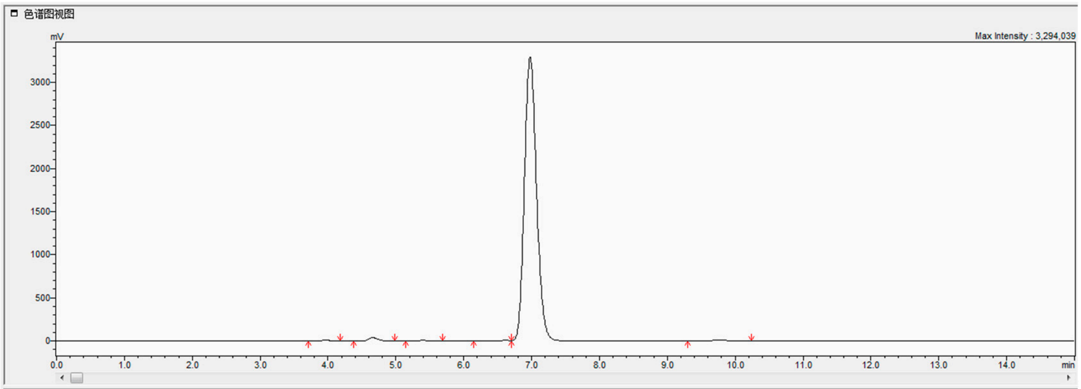

结果视图 - 峰表

峰表 | 化合物 | 组 | 校准曲线

峰号	保留时间	面积	峰宽 (高度50%)	分离度 (USP)	拖尾因	高度	面积%	理论塔板数 (USP)	拖尾因子
1	3.960	83661	0.124	--	1.105	9690	0.200	5001	0.972
2	4.659	383105	0.148	2.889	1.217	38860	0.916	5134	1.274
3	5.403	101869	0.263	2.184	0.000	6006	0.243	2637	--
4	6.612	87309	0.000	2.955	0.000	6681	0.209	4423	--
5	6.979	41037526	0.193	1.007	1.114	3294106	98.068	7141	1.144
6	9.764	152398	0.278	6.859	1.034	8158	0.364	6567	1.000
总计		41845867				3363502	100.000		

Figure S14. ^{1}H NMR, ^{13}C NMR, ^{19}F NMR, HRMS and HPLC for **E14**.

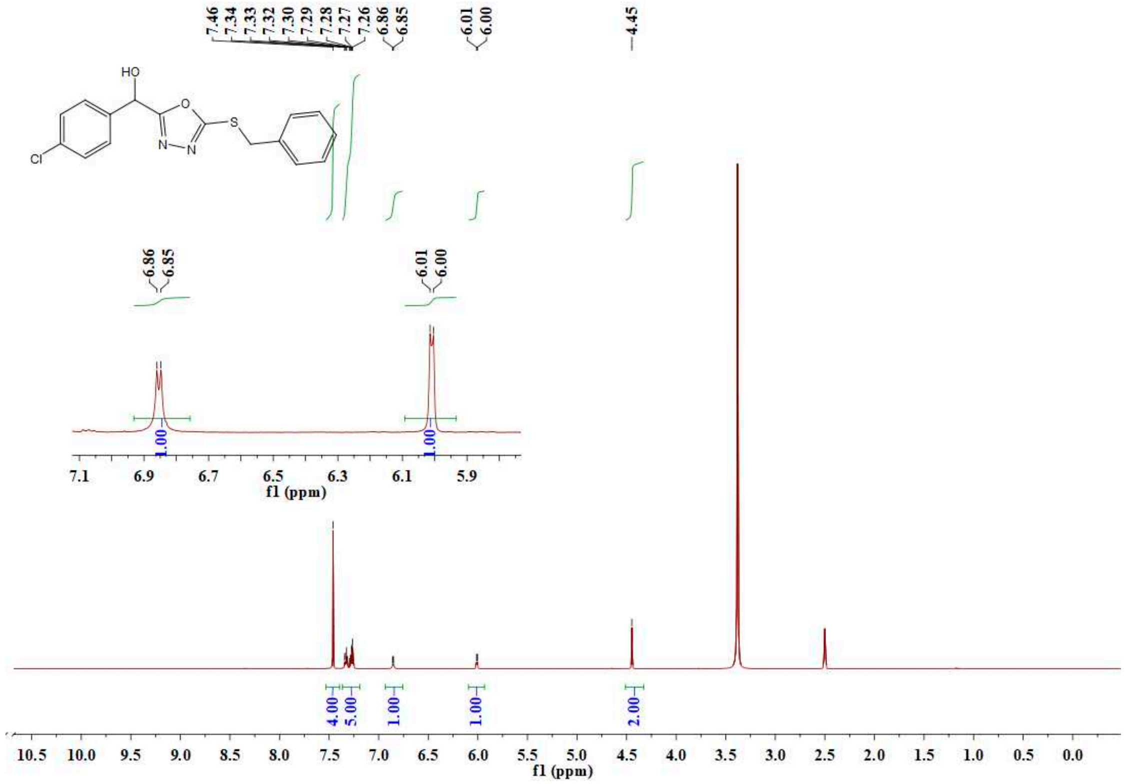
7.46
7.34
7.33
7.32
7.30
7.29
7.28
7.27
7.26
6.86
6.85
6.01
6.00
4.45
HO
O
S
N
N
Cl
4.00
5.00
1.00
1.00
2.00
f1 (ppm)

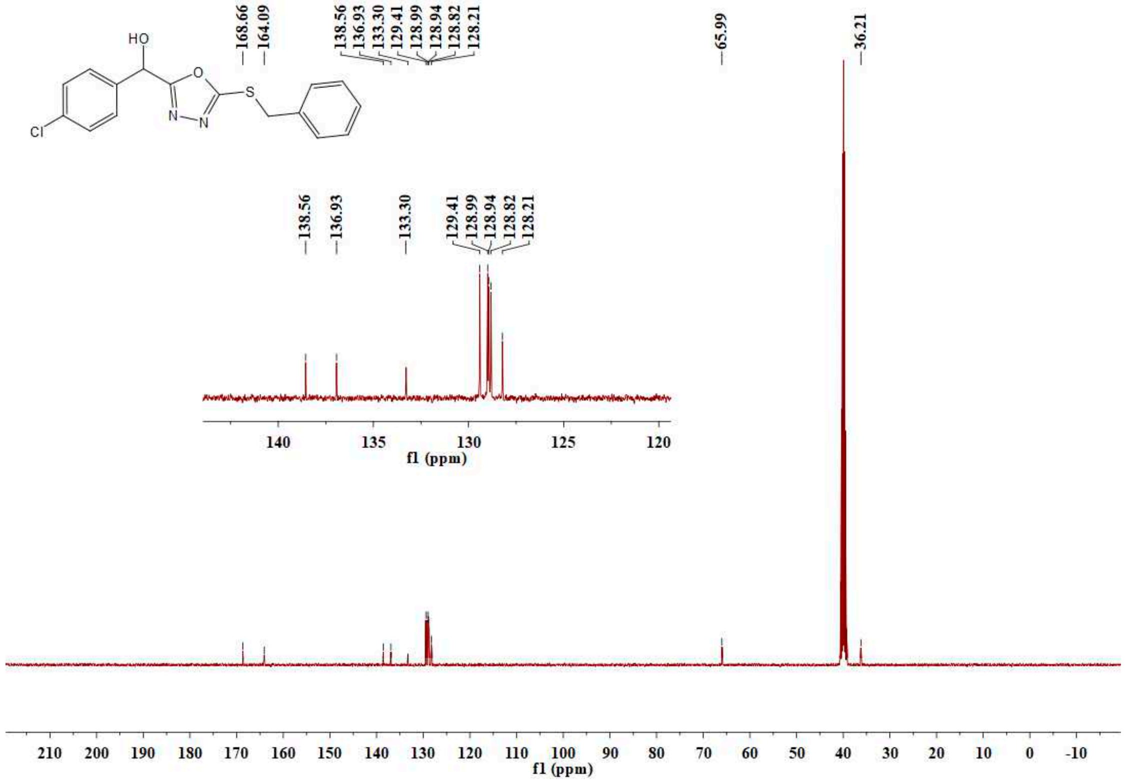
168.66
164.09
138.56
136.93
133.30
129.41
128.99
128.94
128.82
128.21
65.99
36.21
HO
O
S
N
N
Cl
f1 (ppm)

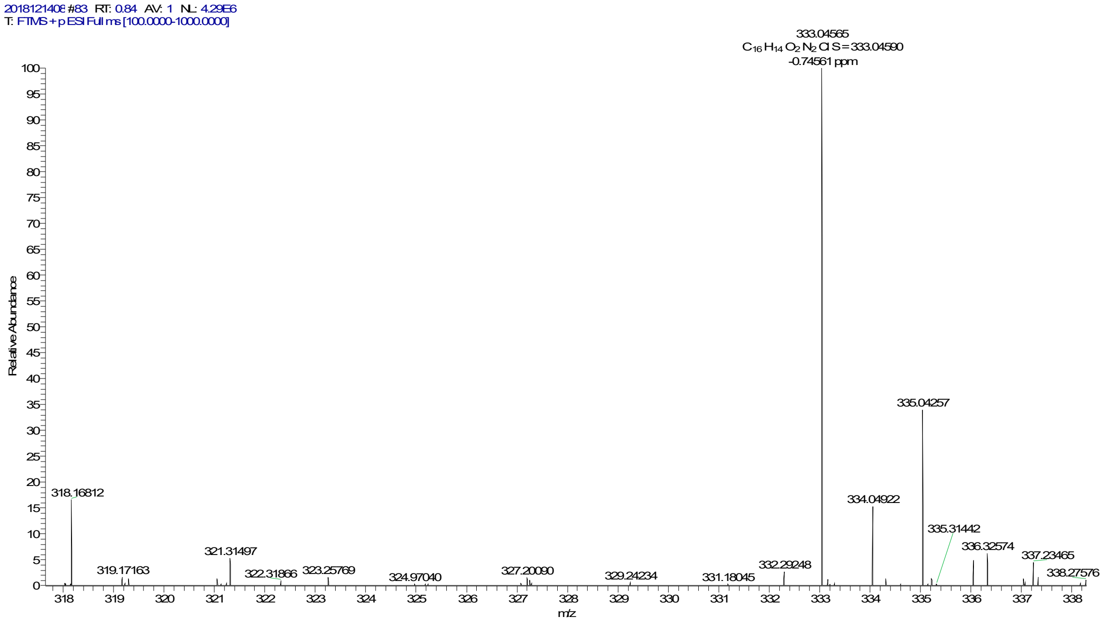

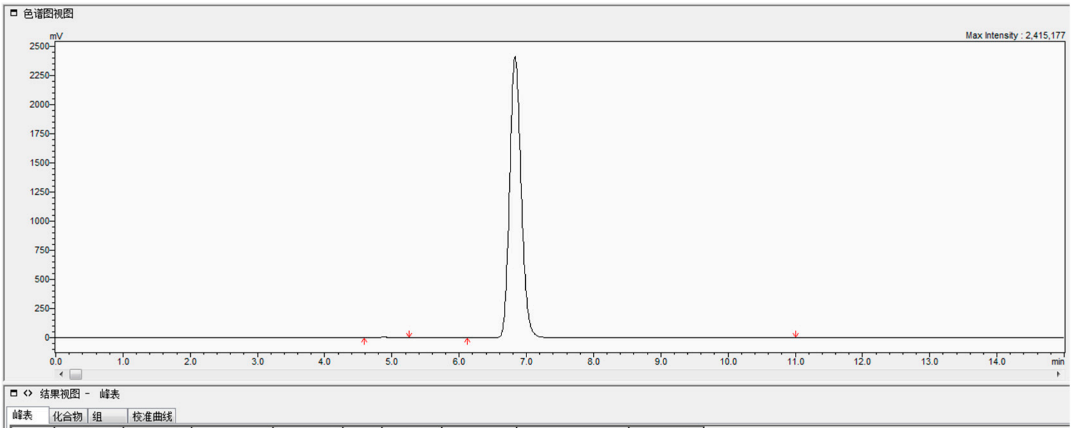

峰号	保留时间	面积	峰宽 (高度50%)	分离度 (USP)	拖尾因	高度	面积%	理论塔板数 (USP)	拖尾因子
1	4.877	57826	0.149	--	1.478	5181	0.200	5411	--
2	6.831	28800247	0.184	6.689	1.134	2415337	99.800	7324	1.164
总计		28858073				2420518	100.000		

Figure S15. ^{1}H NMR, ^{13}C NMR, HRMS, and HPLC for **E_{15}.**

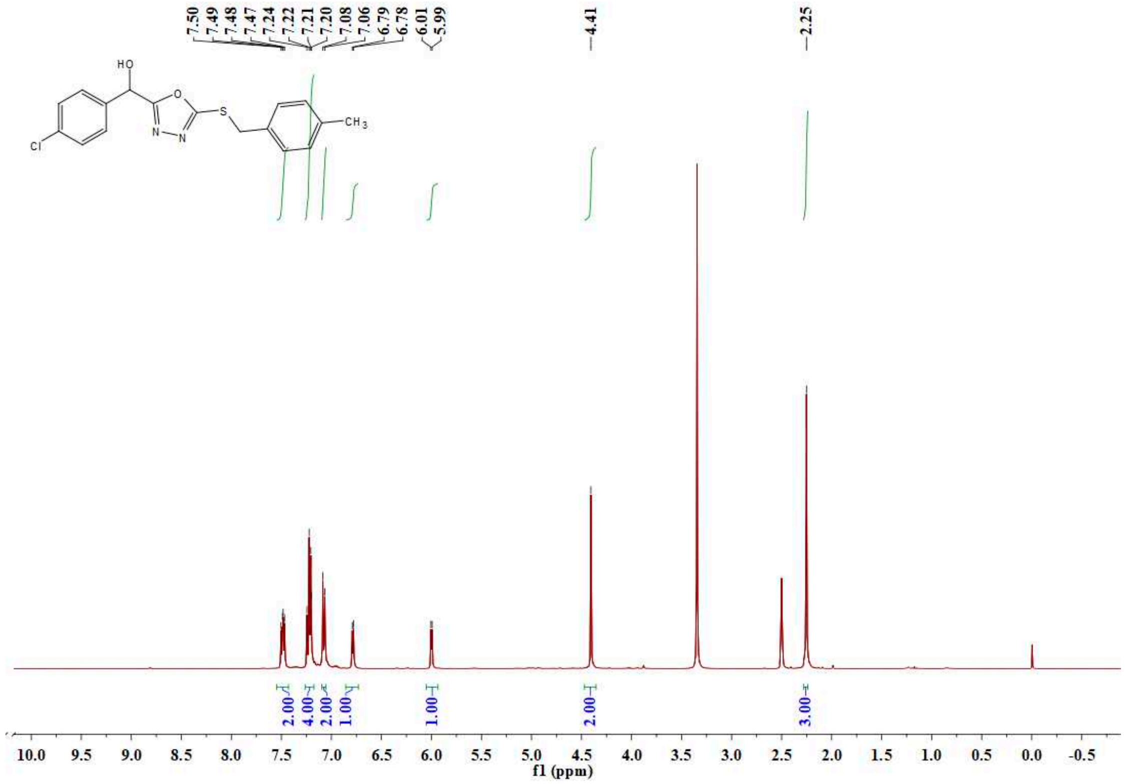
7.50 7.49 7.48 7.47 7.24 7.22 7.21 7.20 7.08 7.06 6.79 6.78 6.01 5.99
4.41
2.25
HO
O
S
CH3
N
N
Cl
2.00 4.00 2.00 1.00 1.00 2.00 3.00
10.0 9.5 9.0 8.5 8.0 7.5 7.0 6.5 6.0 5.5 5.0 4.5 4.0 3.5 3.0 2.5 2.0 1.5 1.0 0.5 0.0 -0.5
f1 (ppm)

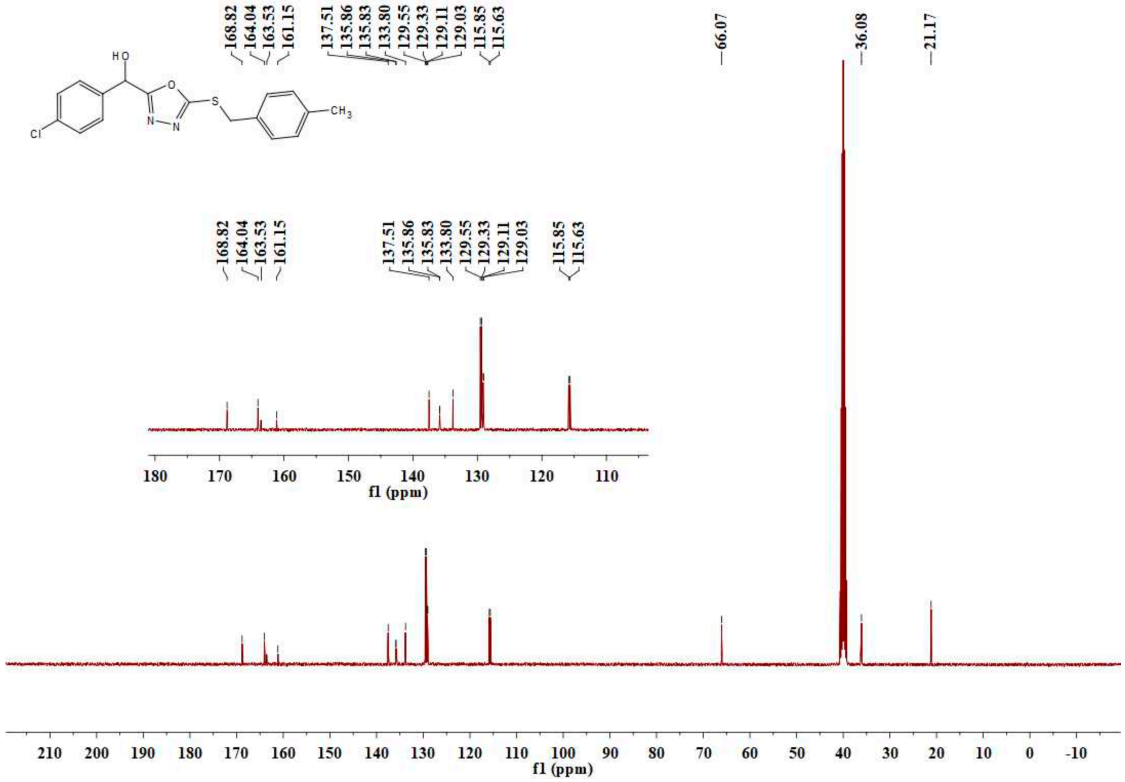
168.82 164.04 163.53 161.15 137.51 135.86 135.83 133.80 129.55 129.33 129.11 129.03 115.85 115.63
66.07
36.08
21.17
HO
O
S
CH3
N
N
Cl
180 170 160 150 140 130 120 110
f1 (ppm)
210 200 190 180 170 160 150 140 130 120 110 100 90 80 70 60 50 40 30 20 10 0 -10
f1 (ppm)

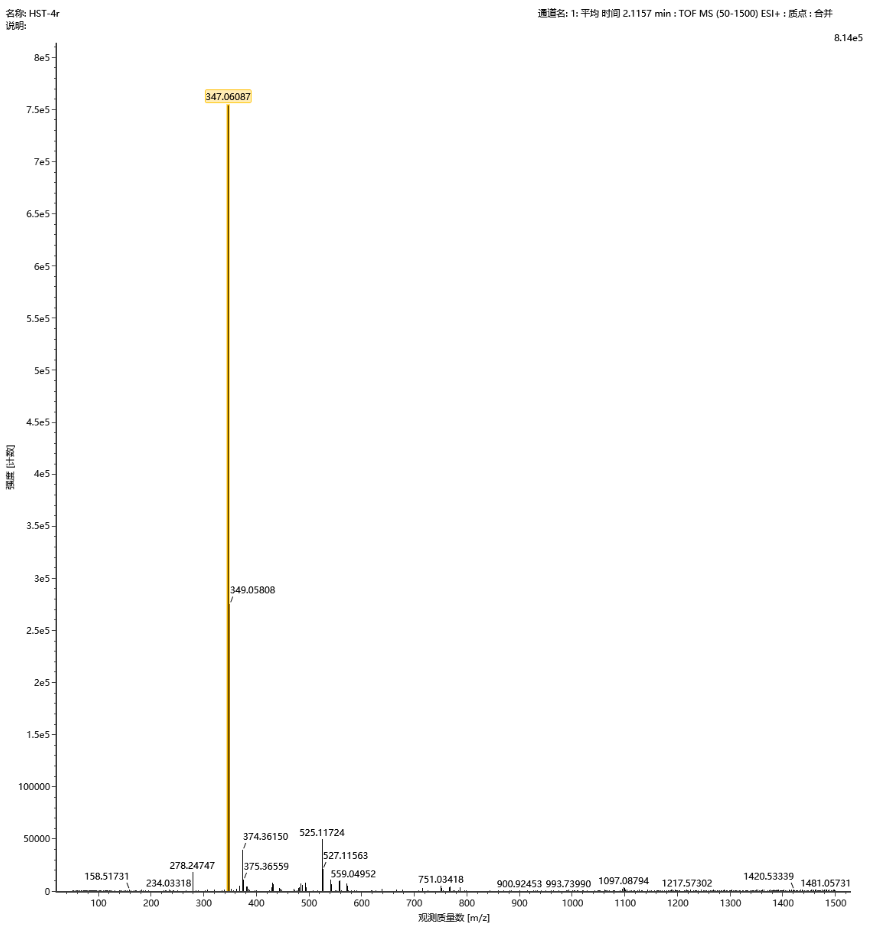

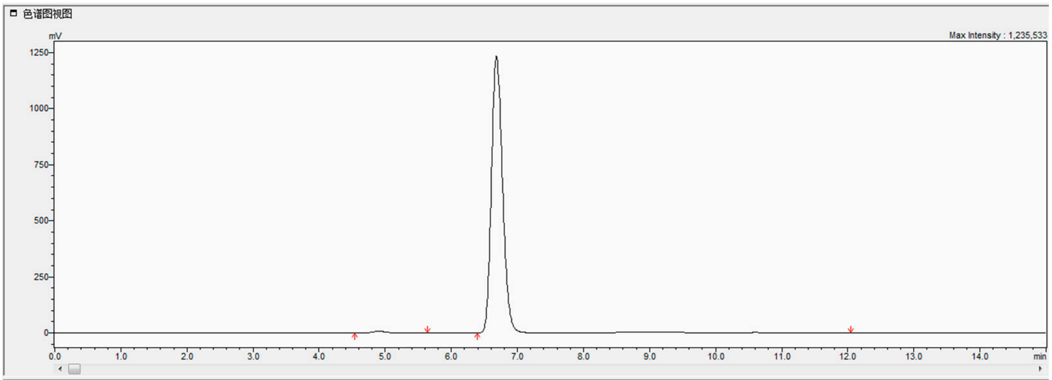

结果视图 - 峰表

峰表 | 化合物 | 组 | 校准曲线

峰号	保留时间	面积	峰宽 (高度50%)	分离度 (USP)	拖尾因	高度	面积%	理论塔板数 (USP)	拖尾因子
1	4.901	125764	0.202	--	1.636	7716	0.870	3114	--
2	6.684	14332728	0.178	5.384	1.159	1235530	99.130	7381	1.190
总计		14458492				1243247	100.000		

Figure S16. ^{1}H NMR, ^{13}C NMR, HRMS, and HPLC for **E_{16}.**

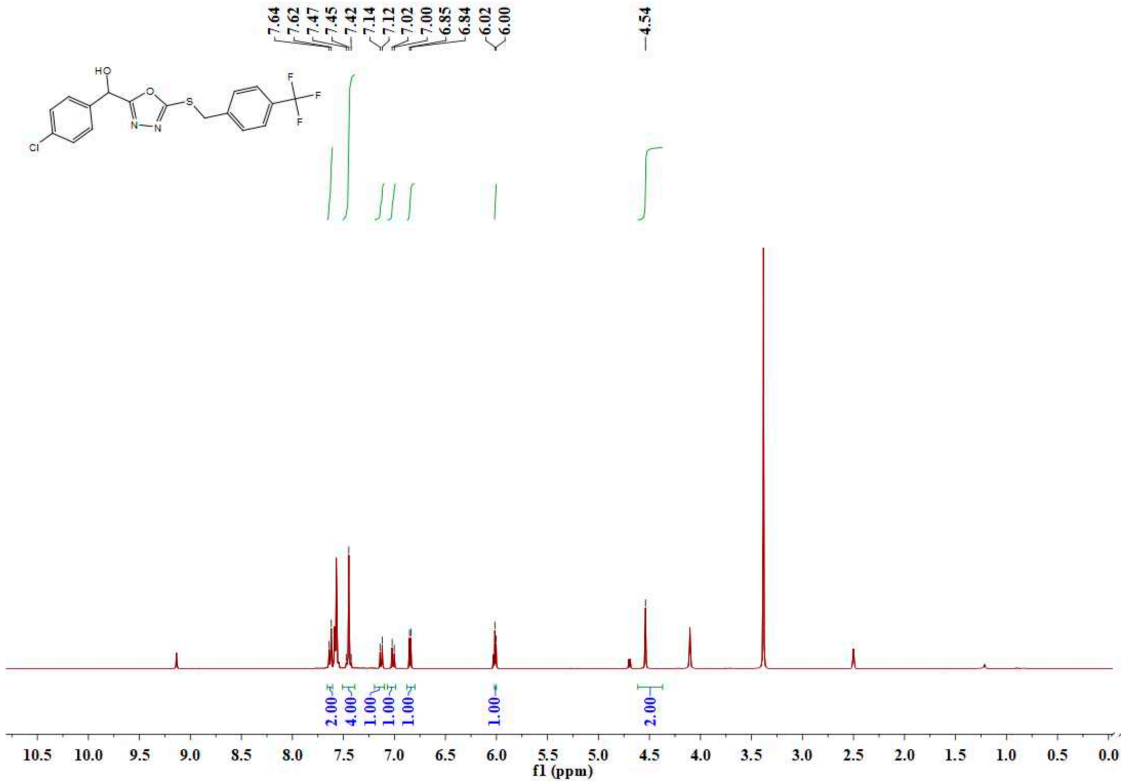
7.64
7.62
7.47
7.45
7.42
7.14
7.12
7.02
7.00
6.85
6.84
6.02
6.00
4.54
2.00
4.00
1.00
1.00
1.00
1.00
2.00
f1 (ppm)

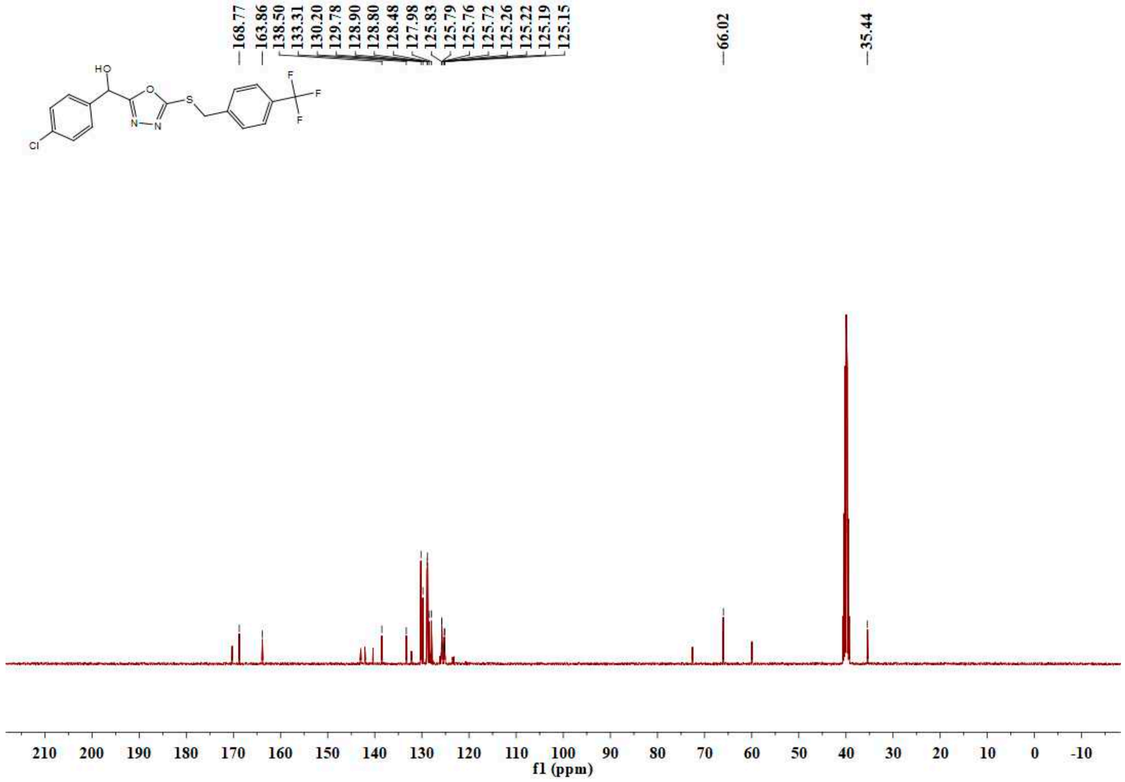
168.77
163.86
138.50
133.31
130.20
129.78
128.90
128.80
128.48
127.98
125.83
125.79
125.76
125.72
125.26
125.22
125.19
125.15
66.02
35.44
f1 (ppm)

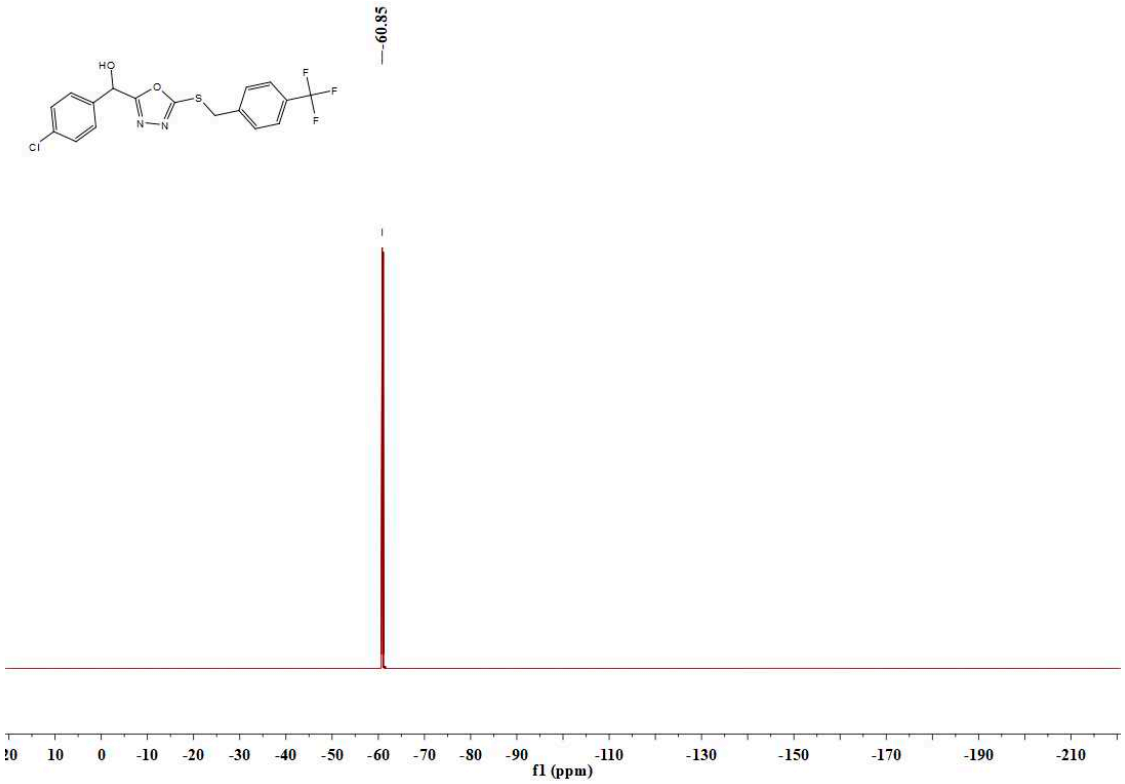
-60.85
HO
O
N–N
S
F
F
F
Cl
20 10 0 -10 -20 -30 -40 -50 -60 -70 -80 -90 -110 -130 -150 -170 -190 -210
f1 (ppm)

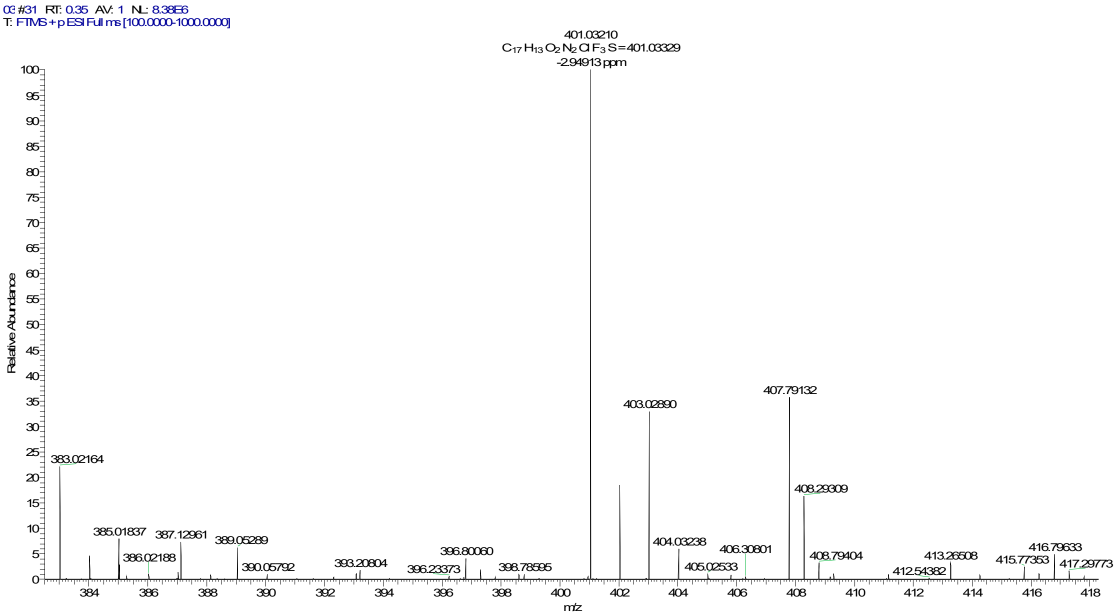
03 #31 RT: 0.35 AV: 1 NL: 8.38E6
T: FTMS + p ESI Full ms [100.0000-1000.0000]
401.03210
$C_{17}H_{13}O_2N_2ClF_3S$ = 401.03329
-2.94913 ppm
Relative Abundance
383.02164
385.01837
386.02188
387.12961
389.05289
390.05792
393.20804
396.23373
396.80060
398.78595
403.02890
404.03238
405.02533
406.30801
407.79132
408.29309
408.79404
412.54382
413.26508
415.77353
416.79633
417.29773
m/z

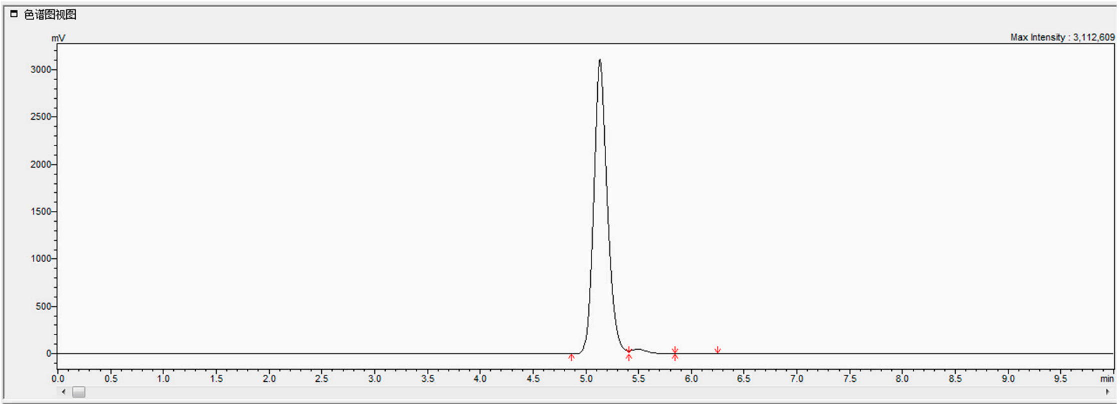

结果视图 - 峰表

峰表 | 化合物 | 组 | 校准曲线

峰号	保留时间	面积	峰宽 (高度50%)	分离度 (USP)	拖尾因	高度	面积%	理论塔板数 (USP)	拖尾因子
1	5.131	27840055	0.135	--	1.168	3112581	98.122	6799	1.199
2	5.496	474265	0.000	1.157	0.000	45796	1.672	3311	--
3	6.018	58720	0.172	1.522	0.000	5145	0.207	6299	--
总计		28373040				3163522	100.000		

Figure S17. ^{1}H NMR, ^{13}C NMR, ^{19}F NMR, HRMS and HPLC for **E$_{17}$**.

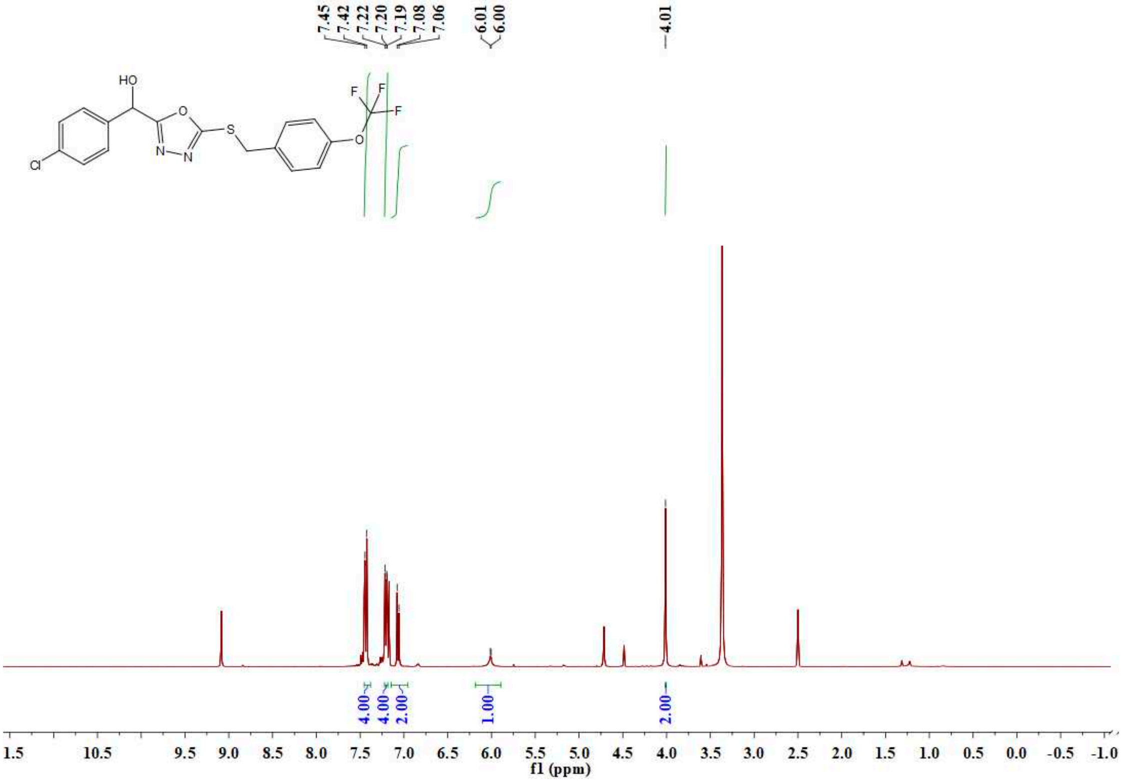

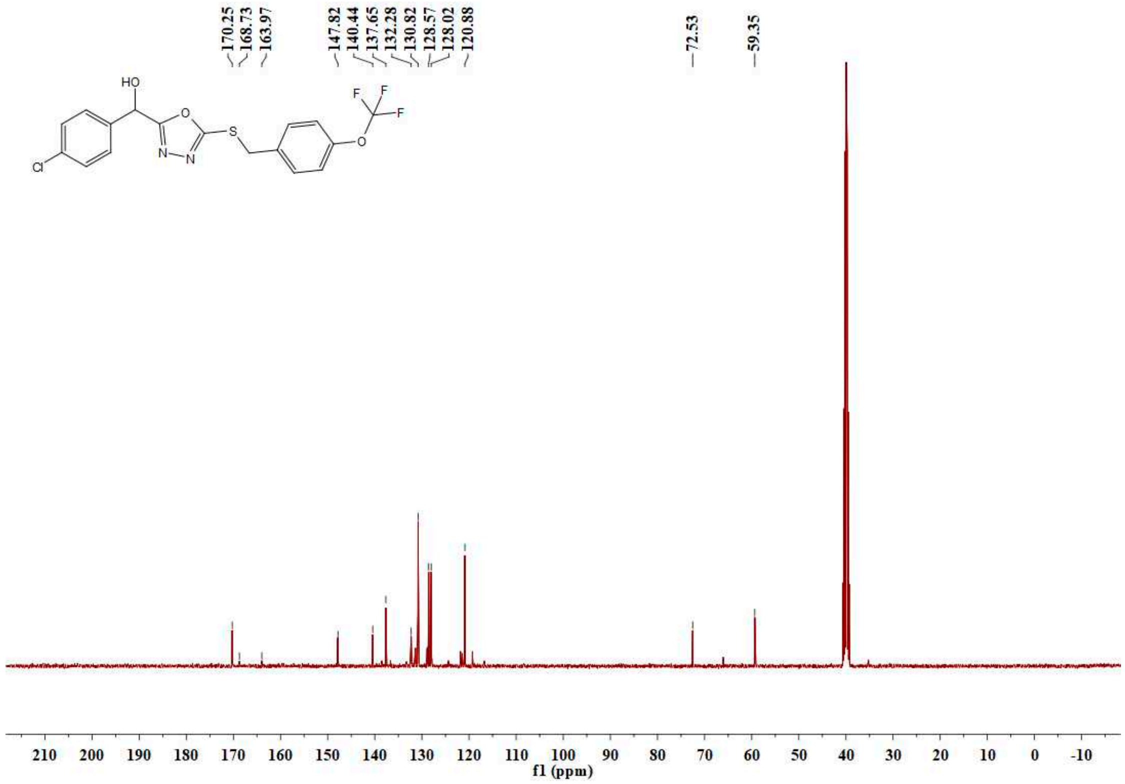
170.25
168.73
163.97
147.82
140.44
137.65
132.28
130.82
128.57
128.02
120.88
72.53
59.35
HO
O
N
N
S
F
F
F
O
Cl
210 200 190 180 170 160 150 140 130 120 110 100 90 80 70 60 50 40 30 20 10 0 -10
f1 (ppm)

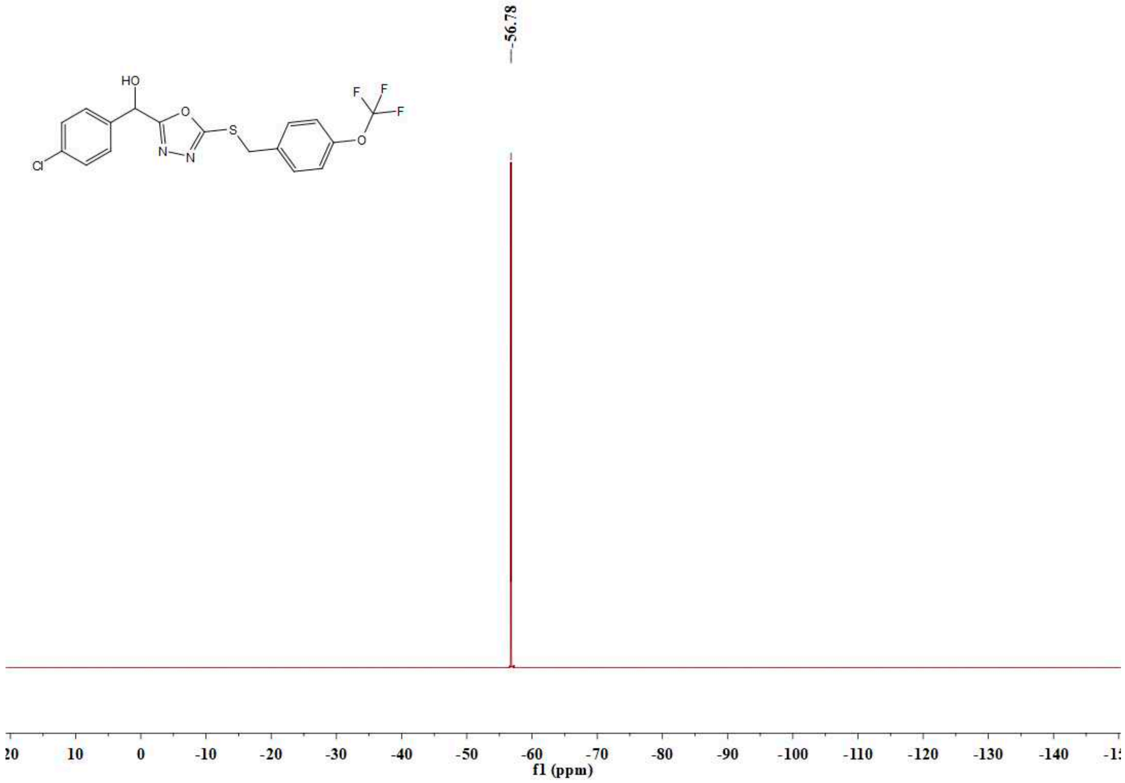
-56.78
HO
O
N
N
S
F
F
F
O
Cl
10 0 -10 -20 -30 -40 -50 -60 -70 -80 -90 -100 -110 -120 -130 -140
f1 (ppm)

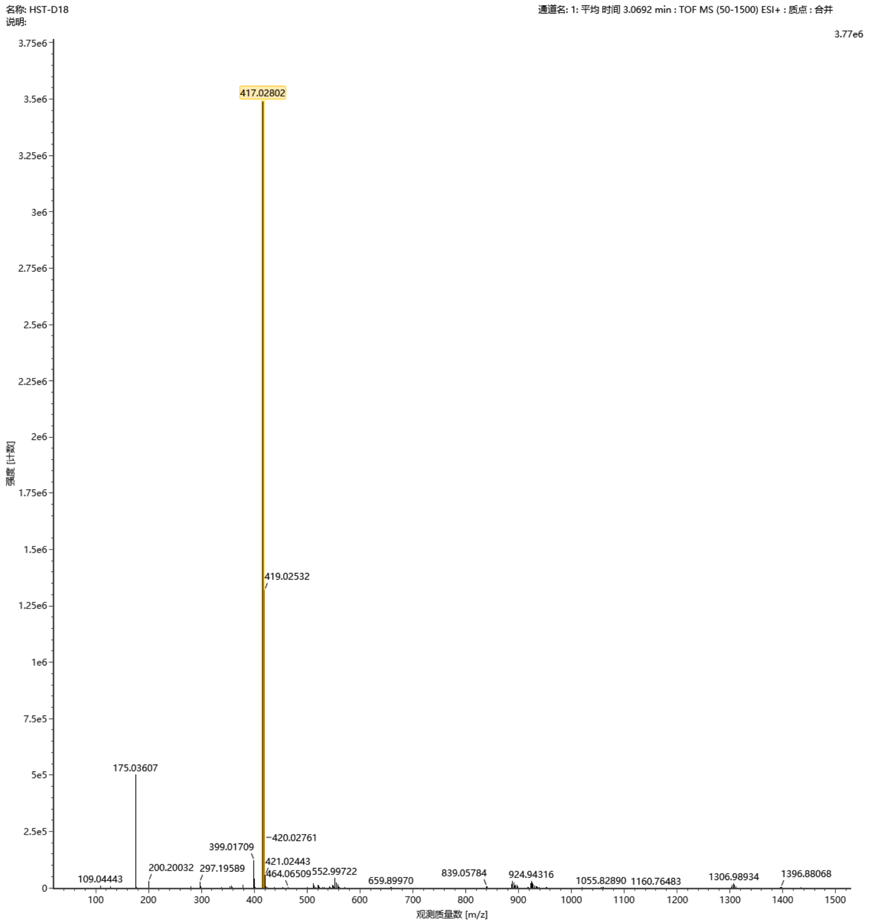

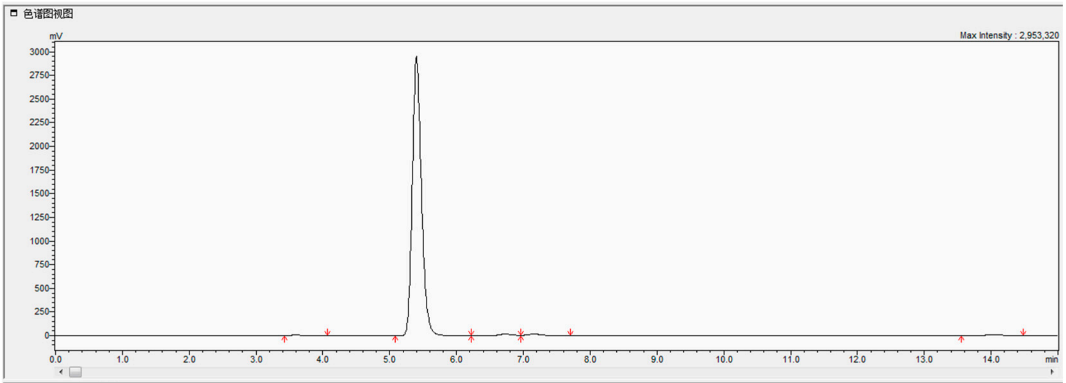

结果视图 - 峰表

峰表 | 化合物 | 组 | 校准曲线

峰号	保留时间	面积	峰宽 (高度50%)	分离度 (USP)	拖尾因	高度	面积%	理论塔板数 (USP)	拖尾因子
1	3.594	83160	0.127	--	1.621	8725	0.288	4012	1.606
2	5.401	28159583	0.145	7.393	1.171	2953057	97.575	6807	1.209
3	6.728	206973	0.195	4.391	0.000	15295	0.717	6159	--
4	7.159	234394	0.209	1.213	0.000	16228	0.812	6086	--
5	14.041	175355	0.340	14.462	1.263	7900	0.608	9229	--
总计		28859465				3001206	100.000		

Figure S18. ^{1}H NMR, ^{13}C NMR, ^{19}F NMR, HRMS and HPLC for **E_{18}.**

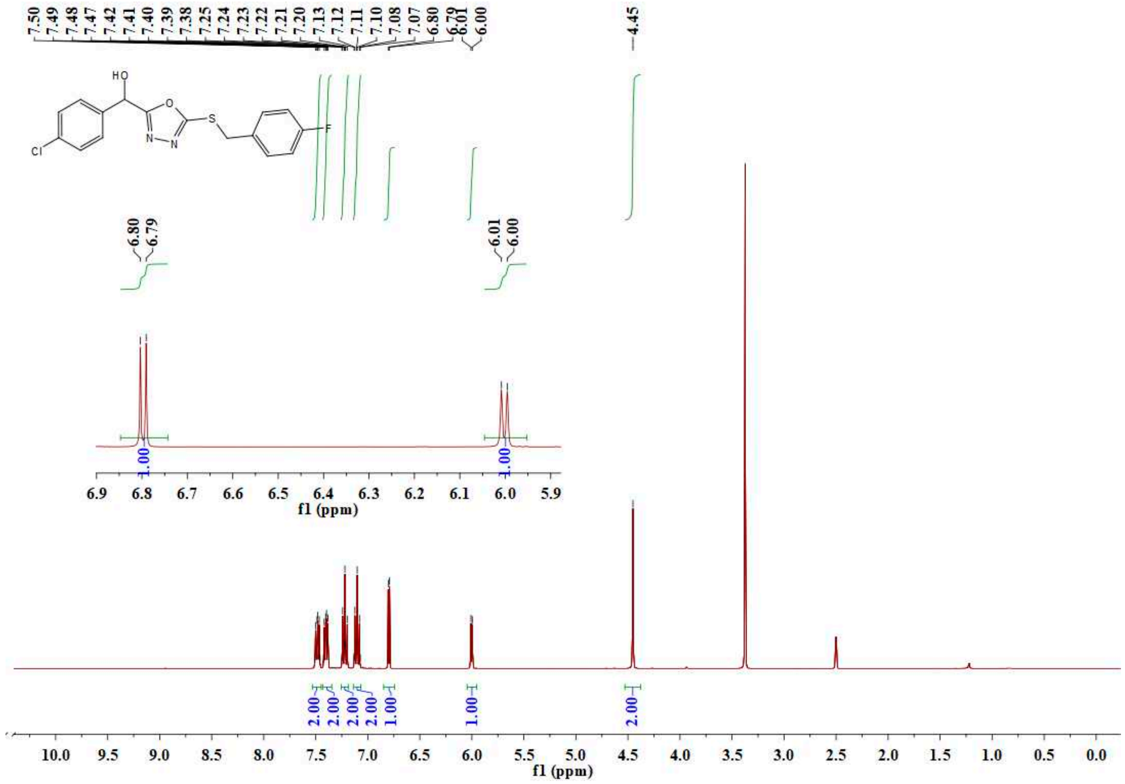
7.50
7.49
7.48
7.47
7.42
7.41
7.40
7.39
7.38
7.25
7.24
7.23
7.22
7.21
7.20
7.13
7.12
7.11
7.10
7.08
7.07
6.80
6.79
6.01
6.00
4.45
HO
O
S
N
N
F
Cl
6.80
6.79
6.01
6.00
1.00
1.00
6.9 6.8 6.7 6.6 6.5 6.4 6.3 6.2 6.1 6.0 5.9
f1 (ppm)
2.00
2.00
2.00
2.00
1.00
1.00
2.00
10.0 9.5 9.0 8.5 8.0 7.5 7.0 6.5 6.0 5.5 5.0 4.5 4.0 3.5 3.0 2.5 2.0 1.5 1.0 0.5 0.0
f1 (ppm)

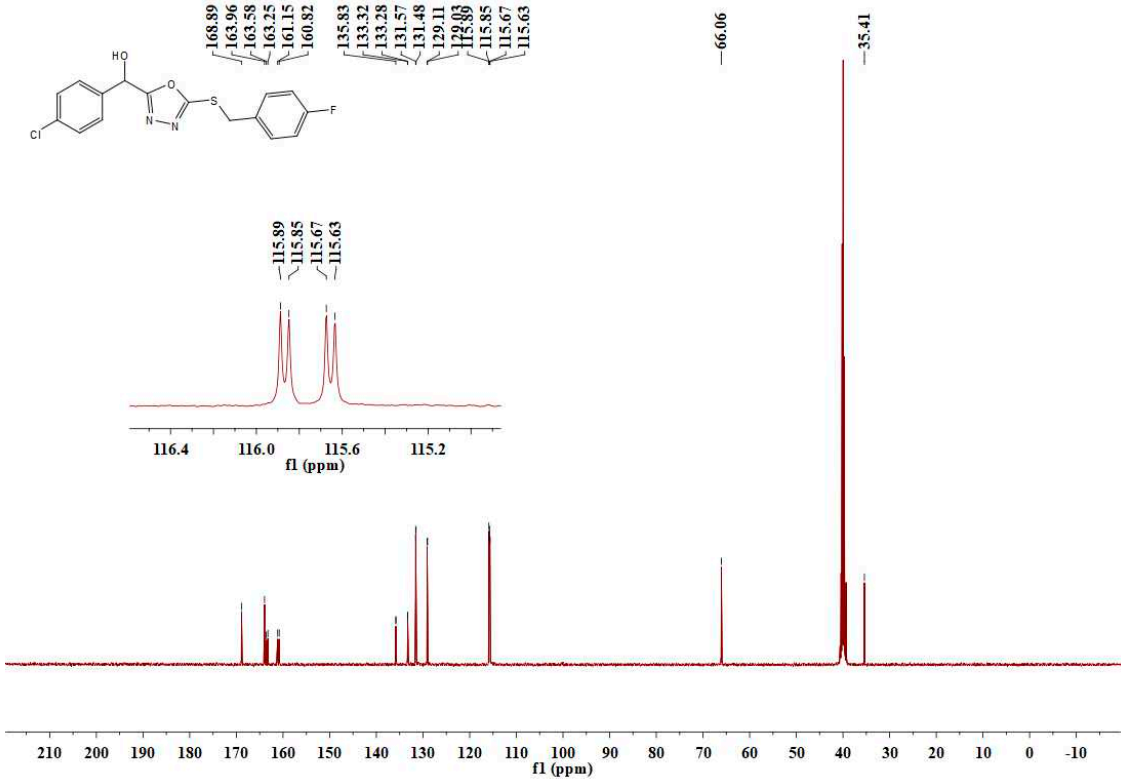
168.89
163.96
163.58
163.25
161.15
160.82
135.83
133.32
133.28
131.57
131.48
129.11
129.03
115.89
115.85
115.67
115.63
66.06
35.41
HO
O
S
N
N
F
Cl
115.89
115.85
115.67
115.63
116.4 116.0 115.6 115.2
f1 (ppm)
210 200 190 180 170 160 150 140 130 120 110 100 90 80 70 60 50 40 30 20 10 0 -10
f1 (ppm)

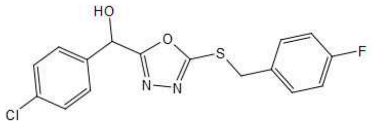
HO
O
S
F
N
N
Cl

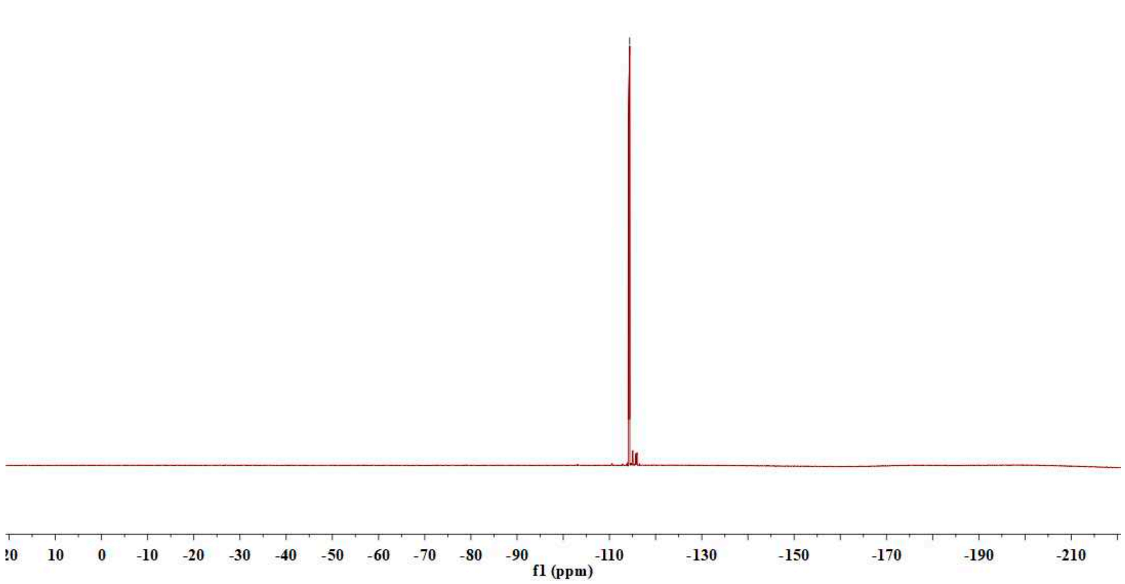
-114.38
f1 (ppm)

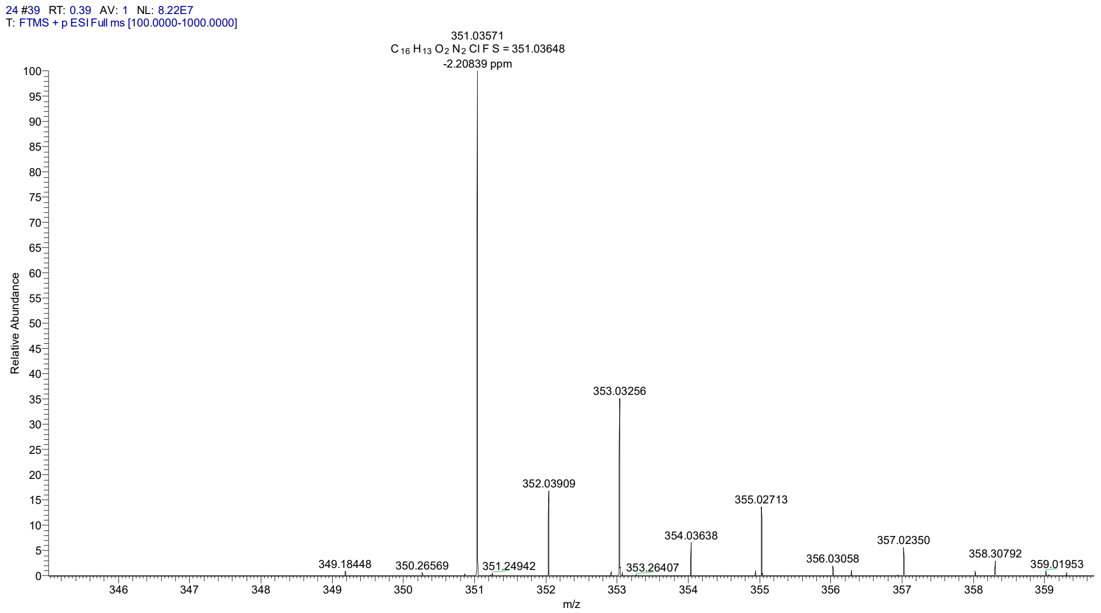
24 #39 RT: 0.39 AV: 1 NL: 8.22E7
T: FTMS + p ESI Full ms [100.0000-1000.0000]
351.03571
C 16 H 13 O 2 N 2 Cl F S = 351.03648
-2.20839 ppm
353.03256
352.03909
355.02713
354.03638
357.02350
358.30792
356.03058
349.18448
350.26569
351.24942
353.26407
359.01953
Relative Abundance
m/z

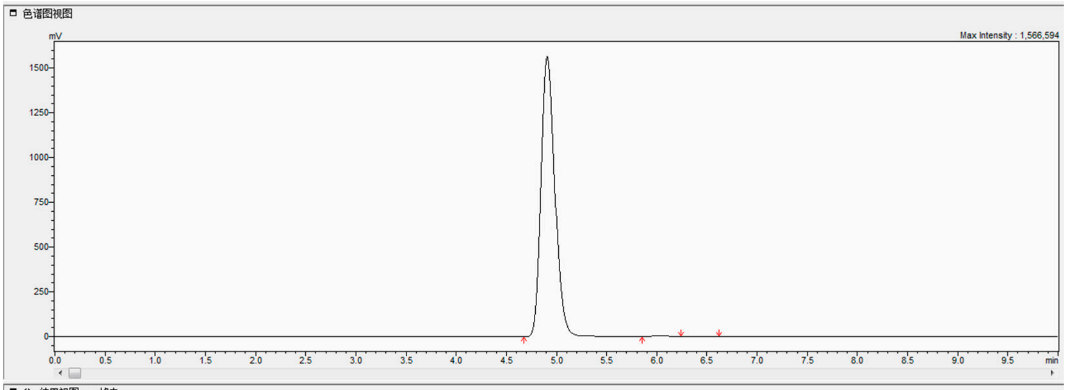

峰号	保留时间	面积	峰宽 (高度50%)	分离度 (USP)	拖尾因	高度	面积%	理论塔板数 (USP)	拖尾因子
1	4.911	14865952	0.146	--	1.164	1566549	99.640	5841	1.197
2	6.027	53658	0.168	4.048	1.109	4921	0.360	6705	--
总计		14919609				1571470	100.000		

Figure S19. ^{1}H NMR, ^{13}C NMR, ^{19}F NMR, HRMS and HPLC for **E19.**

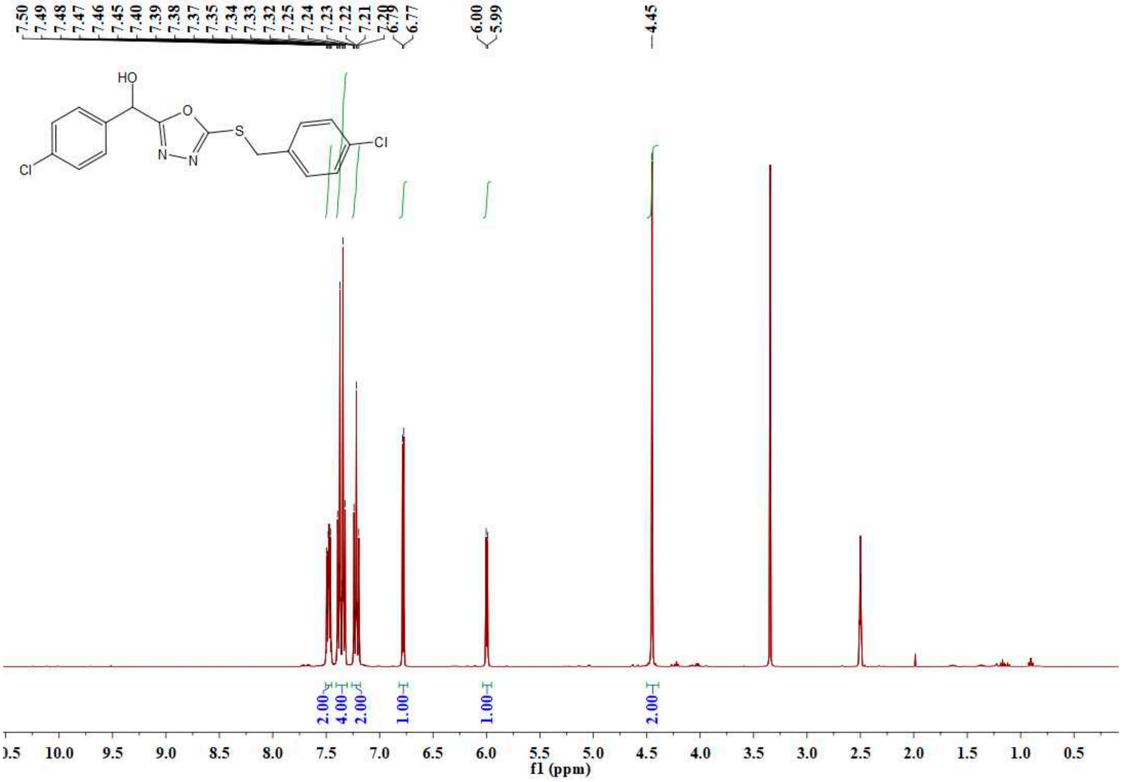

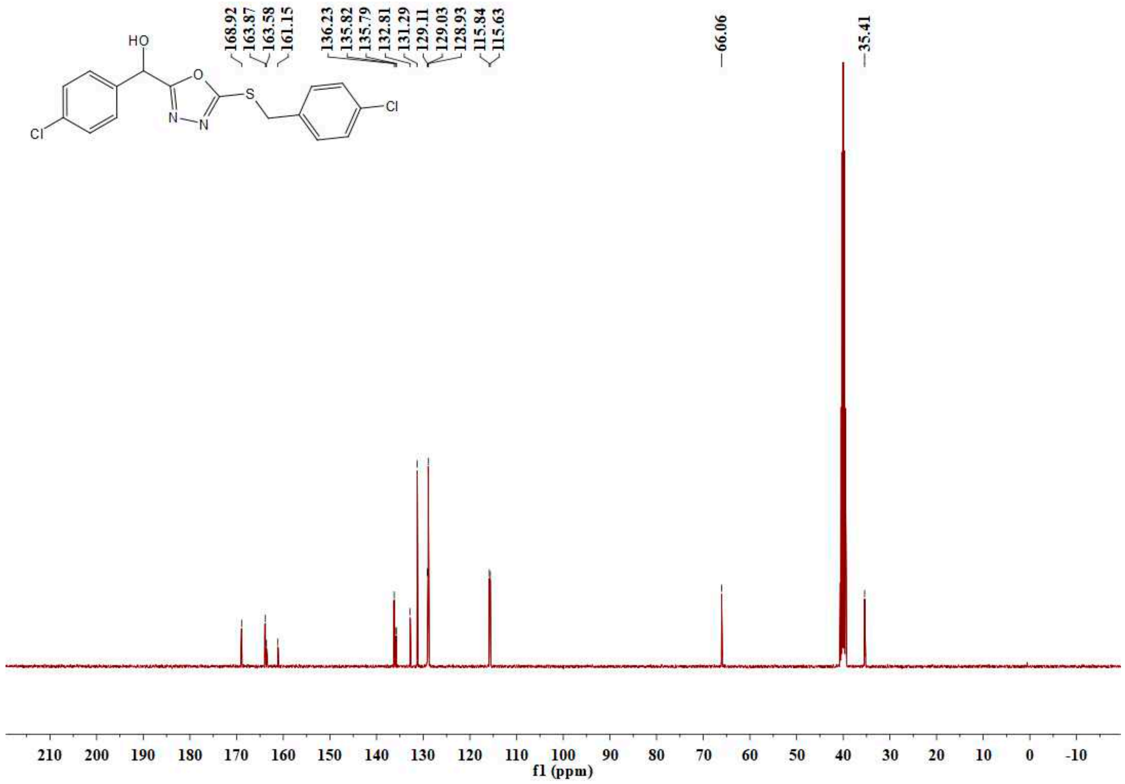
HO
Cl
O
S
N–N
Cl
168.92
163.87
163.58
161.15
136.23
135.82
135.79
132.81
131.29
129.11
129.03
128.93
115.84
115.63
66.06
35.41
210 200 190 180 170 160 150 140 130 120 110 100 90 80 70 60 50 40 30 20 10 0 -10
f1 (ppm)

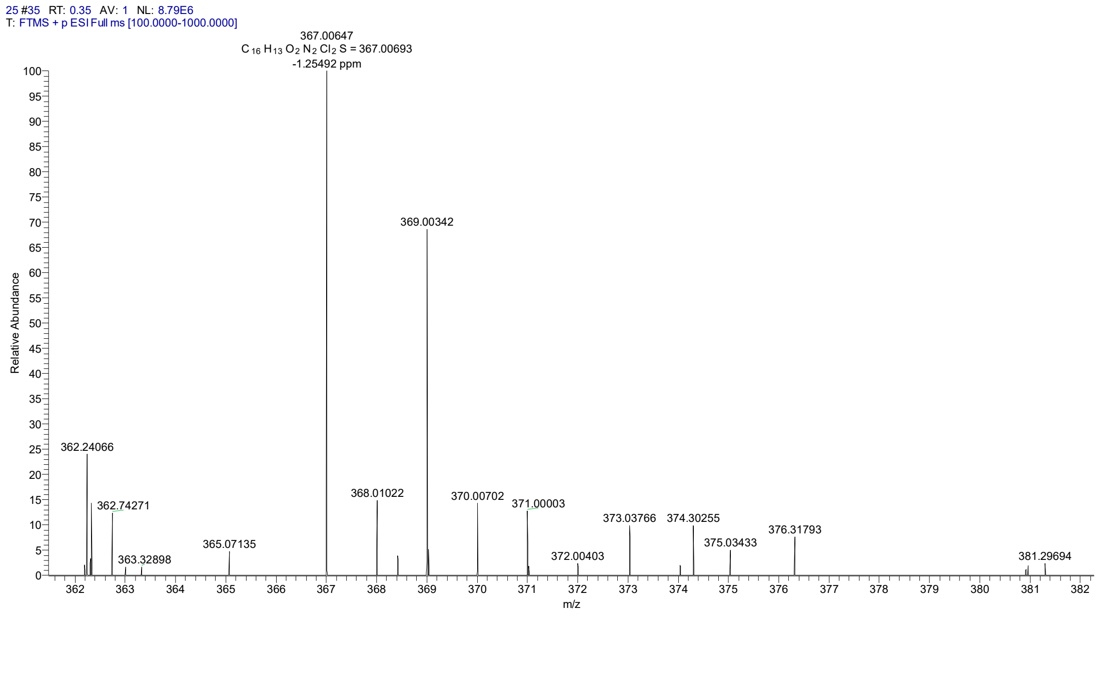
25 #35 RT: 0.35 AV: 1 NL: 8.79E6
T: FTMS + p ESI Full ms [100.0000-1000.0000]
367.00647
C 16 H 13 O 2 N 2 Cl 2 S = 367.00693
-1.25492 ppm
369.00342
362.24066
362.74271
363.32898
365.07135
368.01022
370.00702
371.00003
372.00403
373.03766
374.30255
375.03433
376.31793
381.29694
Relative Abundance
m/z

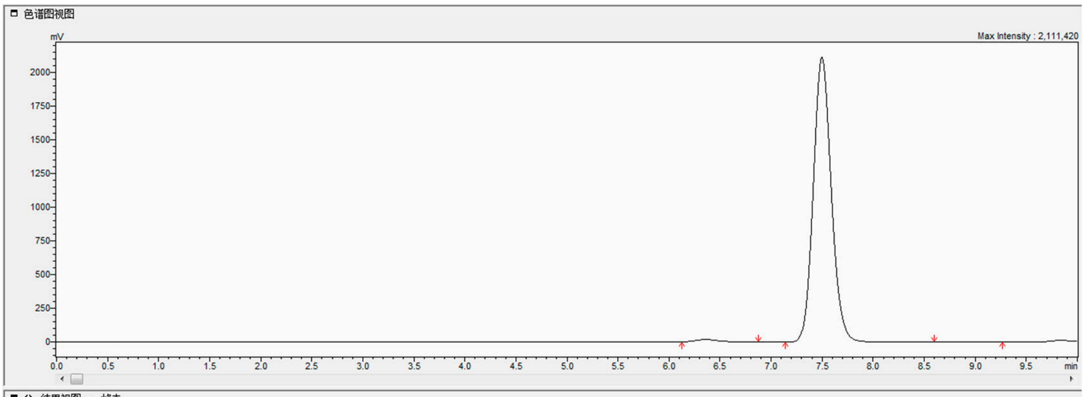

峰号	保留时间	面积	峰宽 (高度50%)	分离度 (USP)	拖尾因	高度	面积%	理论塔板数 (USP)	拖尾因子
1	6.358	282677	0.244	--	1.158	17971	1.065	3655	--
2	7.496	26076244	0.189	3.029	1.096	2111360	98.244	8199	1.123
3	9.837	183332	0.239	6.290	1.142	11721	0.691	9073	--
总计		26542252				2141052	100.000		

Figure S20. ^{1}H NMR, ^{13}C NMR, HRMS, and HPLC for **E$_{20}$.**

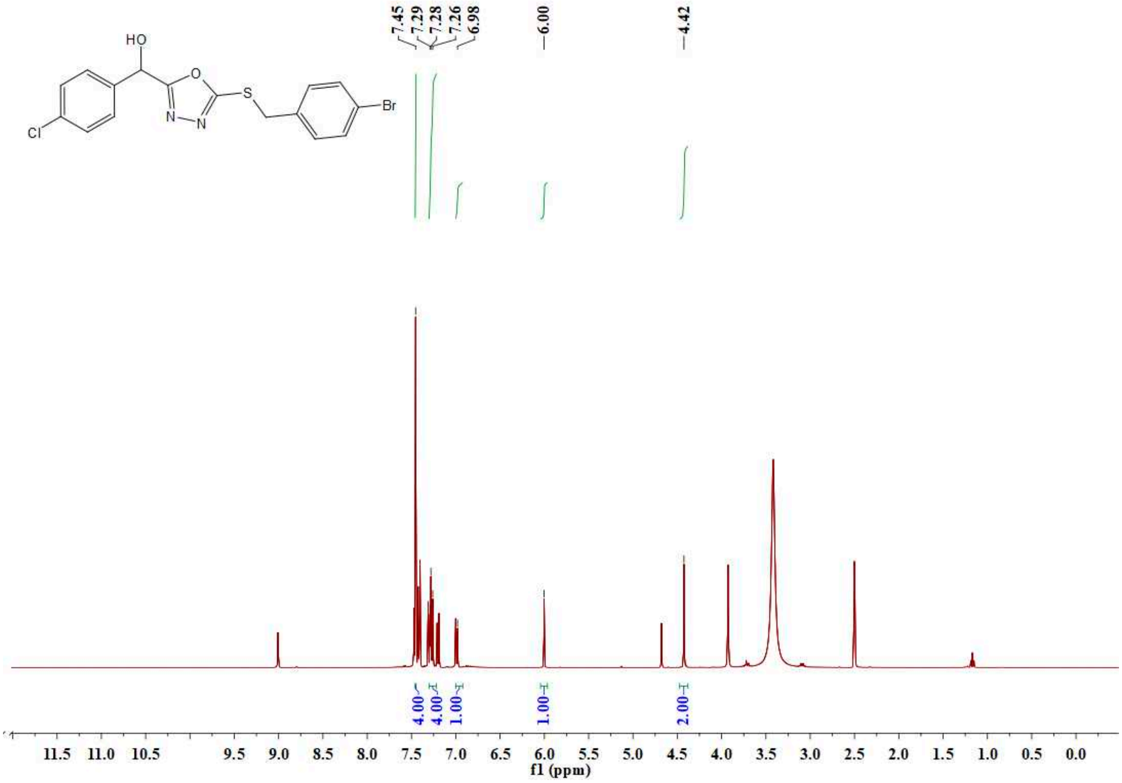

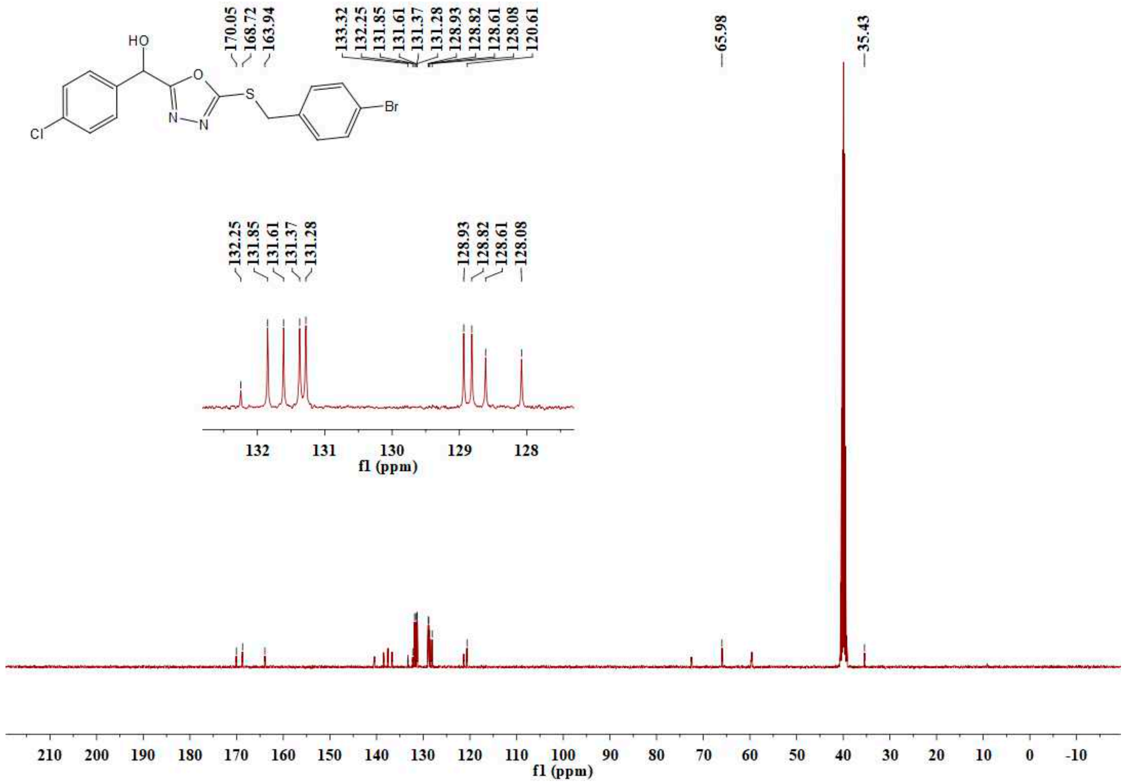
170.05
168.72
163.94
133.32
132.25
131.85
131.61
131.37
131.28
128.93
128.82
128.61
128.08
120.61
65.98
35.43
f1 (ppm)

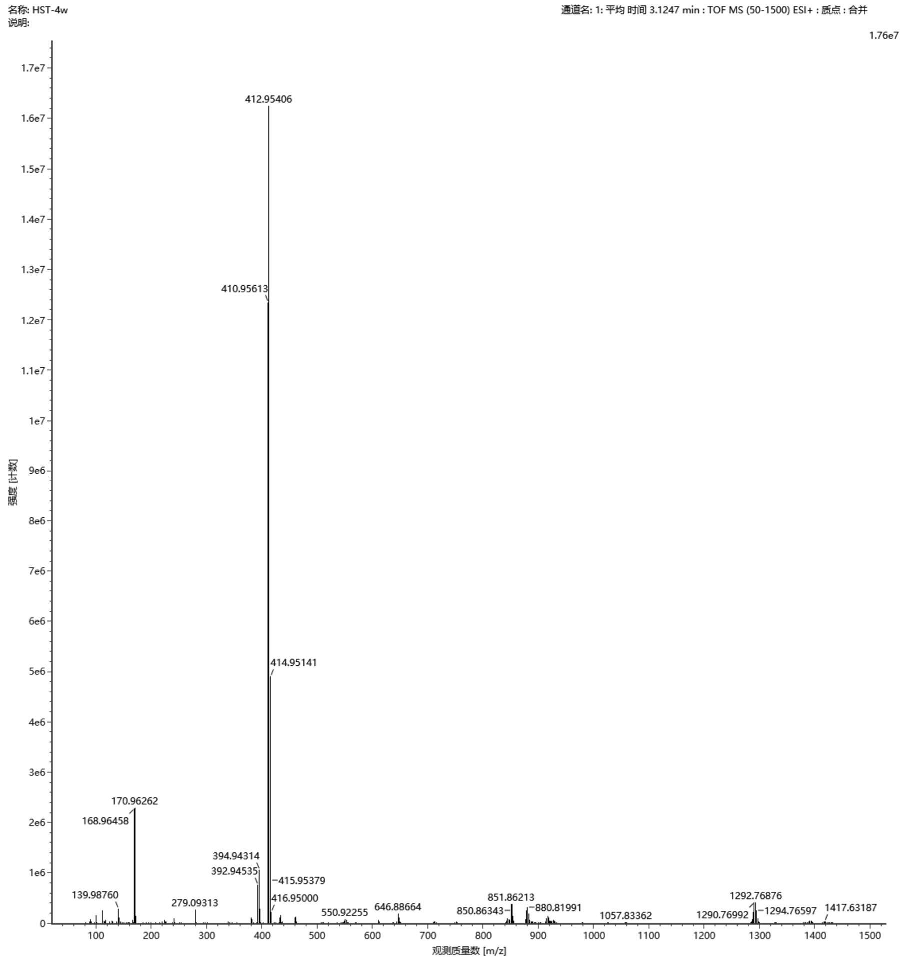
名称: HST-4w
说明:
通道名: 1: 平均 时间 3.1247 min : TOF MS (50-1500) ESI+ : 质点 : 合并
1.76e7
412.95406
410.95613
414.95141
170.96262
168.96458
394.94314
392.94535
415.95379
416.95000
139.98760
279.09313
550.92255
646.88664
851.86213
850.86343
880.81991
1057.83362
1292.76876
1290.76992
1294.76597
1417.63187
强度 [计数]
观测质量数 [m/z]

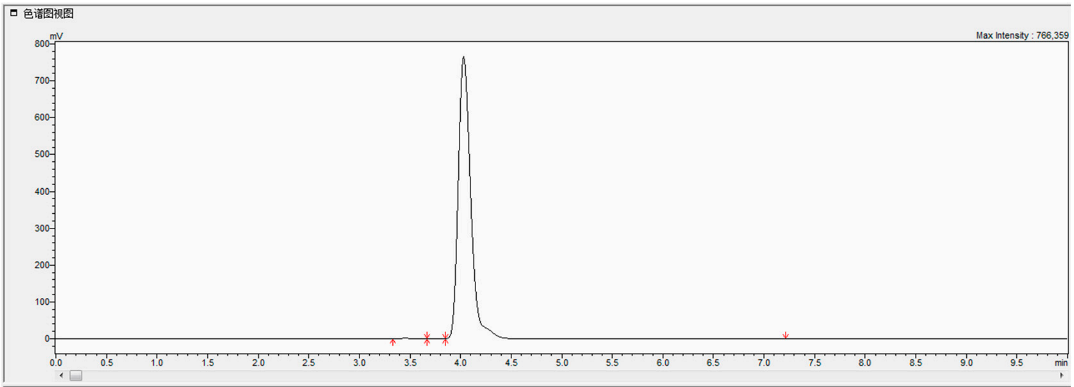

峰号	保留时间	面积	峰宽 (高度50%)	分离度 (USP)	拖尾因	高度	面积%	理论塔板数 (USP)	拖尾因子
1	3.454	13496	0.125	--	0.000	1483	0.204	3733	--
2	3.788	7978	0.000	1.203	0.000	1028	0.120	2135	--
3	4.033	6608676	0.129	0.882	1.203	766372	99.676	4942	1.290
总计		6630150				768884	100.000		

Figure S21. ^{1}H NMR, ^{13}C NMR, HRMS, and HPLC for **E$_{21}$.**

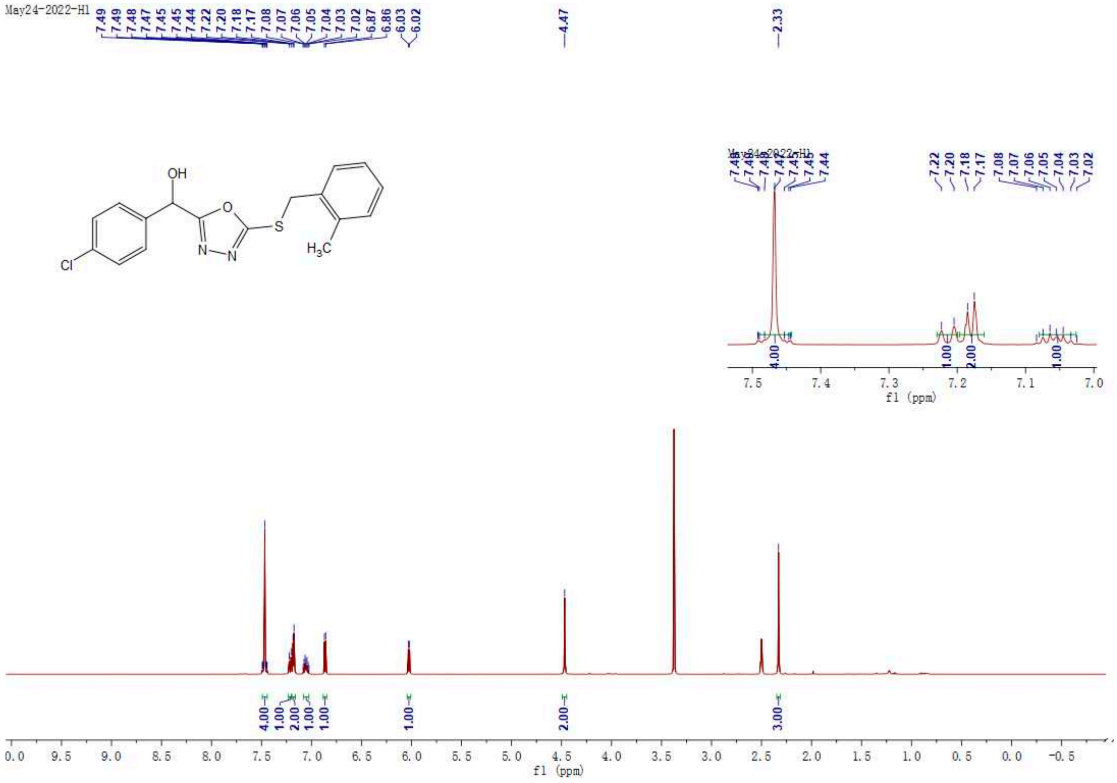

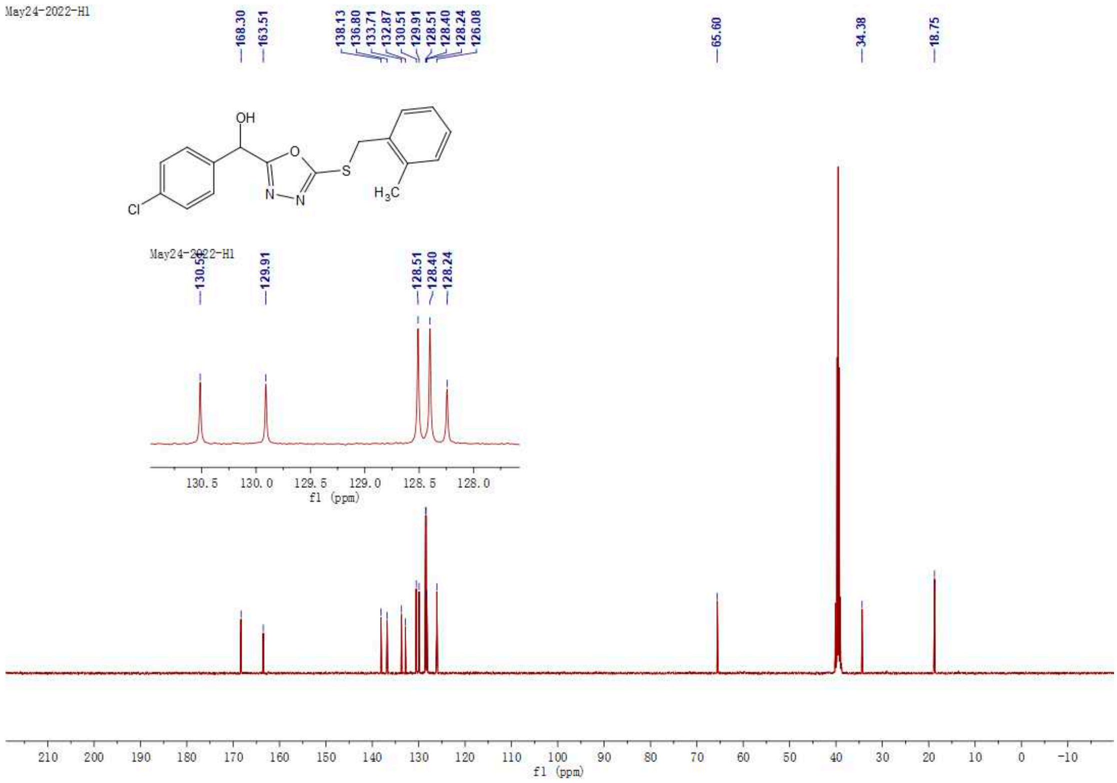
May24-2022-H1
168.30
163.51
138.13
136.80
133.71
132.87
130.51
129.91
128.51
128.40
128.24
126.08
65.60
34.38
18.75
OH
O
S
Cl
N
N
H3C
May24-2022-H1
130.51
129.91
128.51
128.40
128.24
130.5 130.0 129.5 129.0 128.5 128.0
f1 (ppm)
210 200 190 180 170 160 150 140 130 120 110 100 90 80 70 60 50 40 30 20 10 0 -10
f1 (ppm)

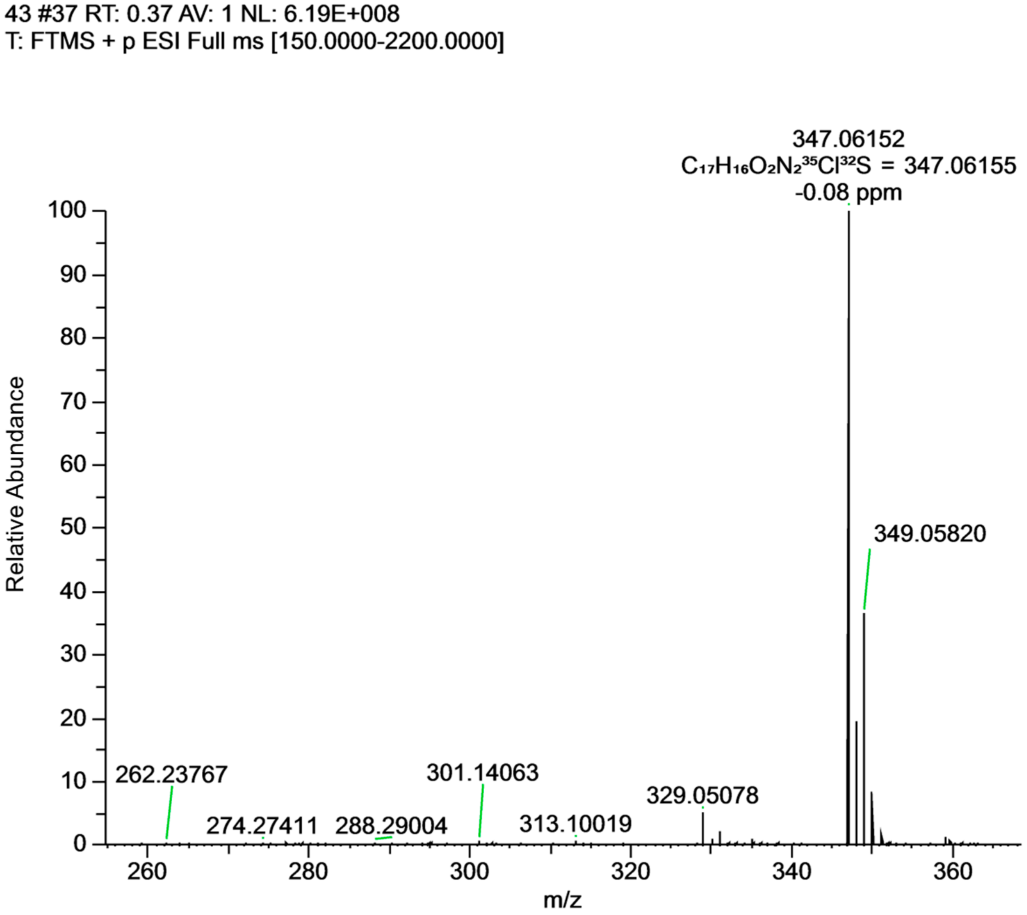
43 #37 RT: 0.37 AV: 1 NL: 6.19E+008
T: FTMS + p ESI Full ms [150.0000-2200.0000]
347.06152
C17H16O2N2 35Cl 32S = 347.06155
-0.08 ppm
349.05820
262.23767
274.27411
288.29004
301.14063
313.10019
329.05078
Relative Abundance
m/z

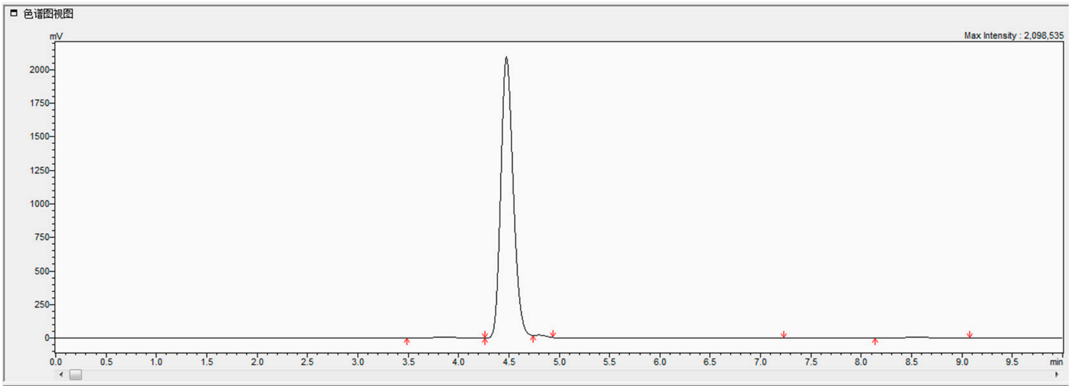

结果视图 - 峰表

峰表 | 化合物 | 组 | 校准曲线

峰号	保留时间	面积	峰宽 (高度50%)	分离度 (USP)	拖尾因	高度	面积%	理论塔板数 (USP)	拖尾因子
1	3.833	127179	0.241	--	0.000	6834	0.681	1378	--
2	4.475	18383607	0.133	1.973	1.175	2098526	98.496	5657	1.209
3	4.798	44133	0.107	1.573	1.682	7159	0.236	12412	1.686
4	8.546	109494	0.240	12.768	1.124	6970	0.587	6787	1.160
总计		18664412				2119489	100.000		

Figure S22. ^{1}H NMR, ^{13}C NMR, HRMS, and HPLC for **E_{22}**.

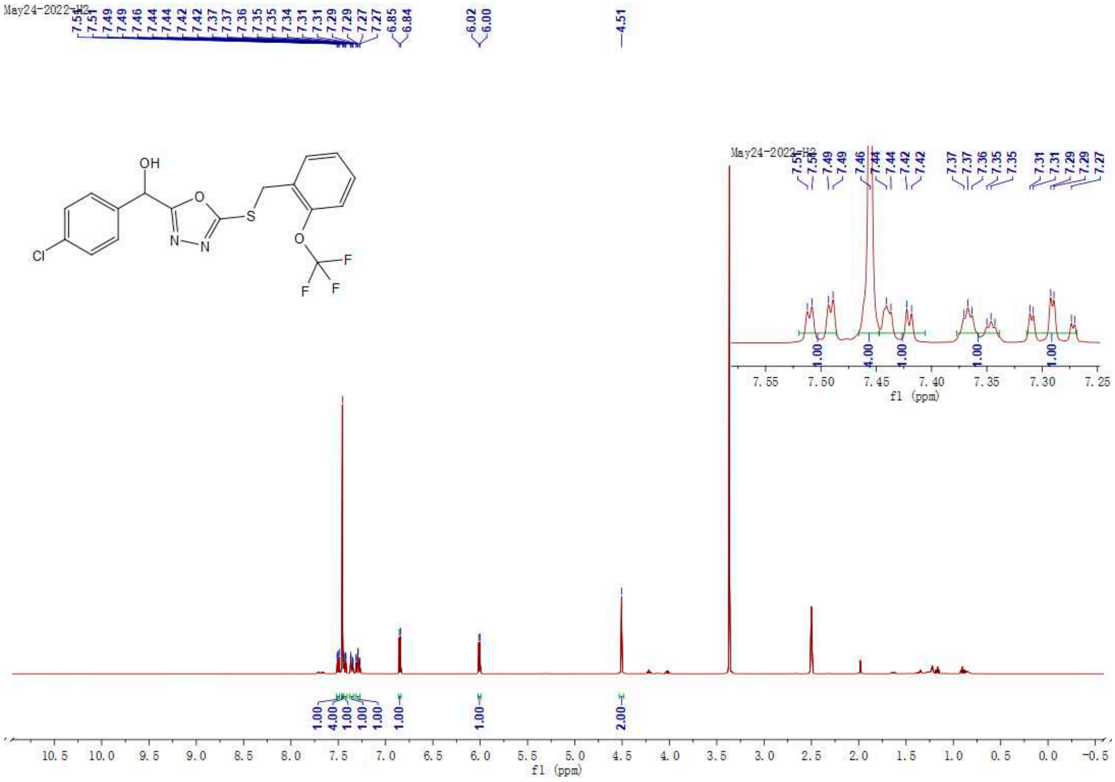

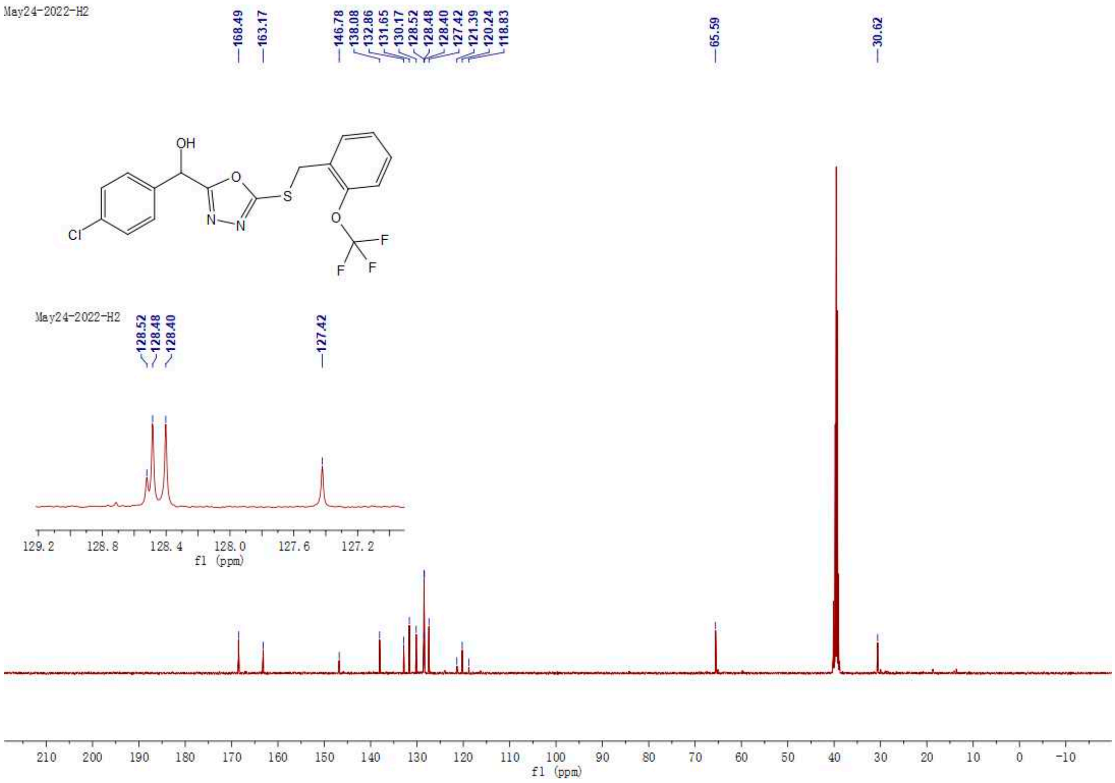
May24-2022-H2
168.49
163.17
146.78
138.08
132.86
131.65
130.17
128.52
128.48
128.40
127.42
121.39
120.24
118.83
65.59
30.62
OH
O
S
O
N
N
Cl
F
F
F
May24-2022-H2
128.52
128.48
128.40
127.42
129.2 128.8 128.4 128.0 127.6 127.2
f1 (ppm)
210 200 190 180 170 160 150 140 130 120 110 100 90 80 70 60 50 40 30 20 10 0 -10
f1 (ppm)

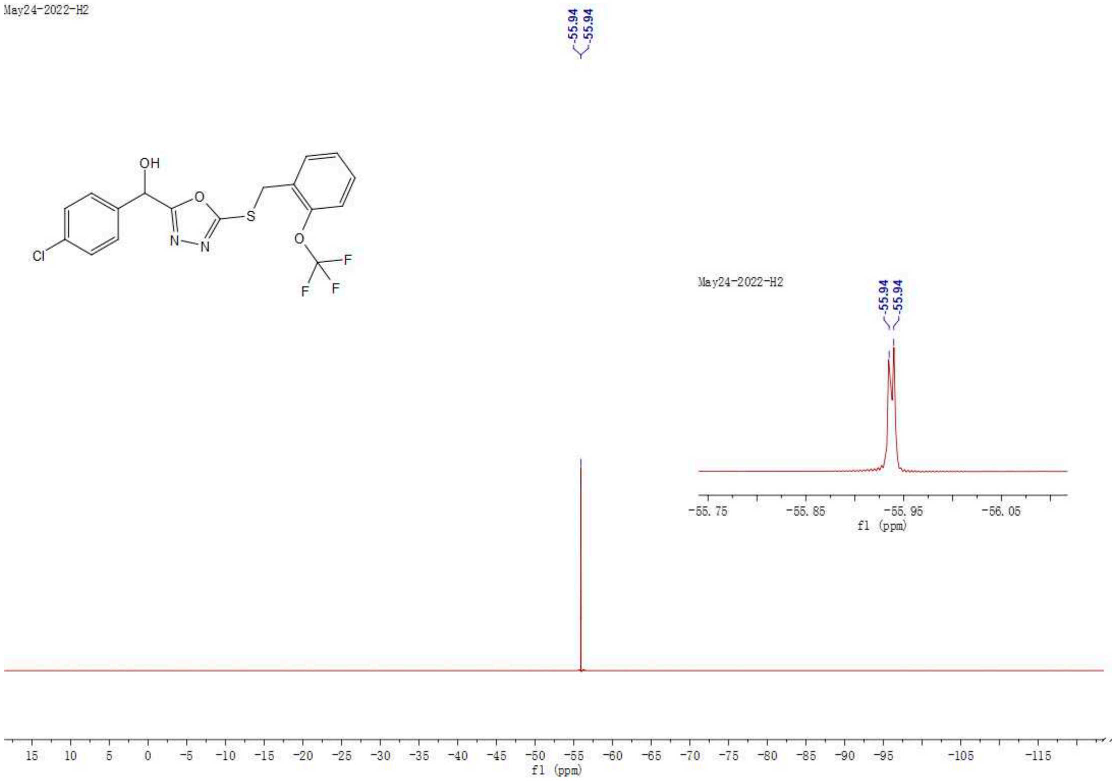
May24-2022-H2
-55.94
-55.94
OH
O
S
O
N
N
Cl
F
F
F
May24-2022-H2
-55.94
-55.94
-55.75 -55.85 -55.95 -56.05
f1 (ppm)
15 10 5 0 -5 -10 -15 -20 -25 -30 -35 -40 -45 -50 -55 -60 -65 -70 -75 -80 -85 -90 -95 -105 -115
f1 (ppm)

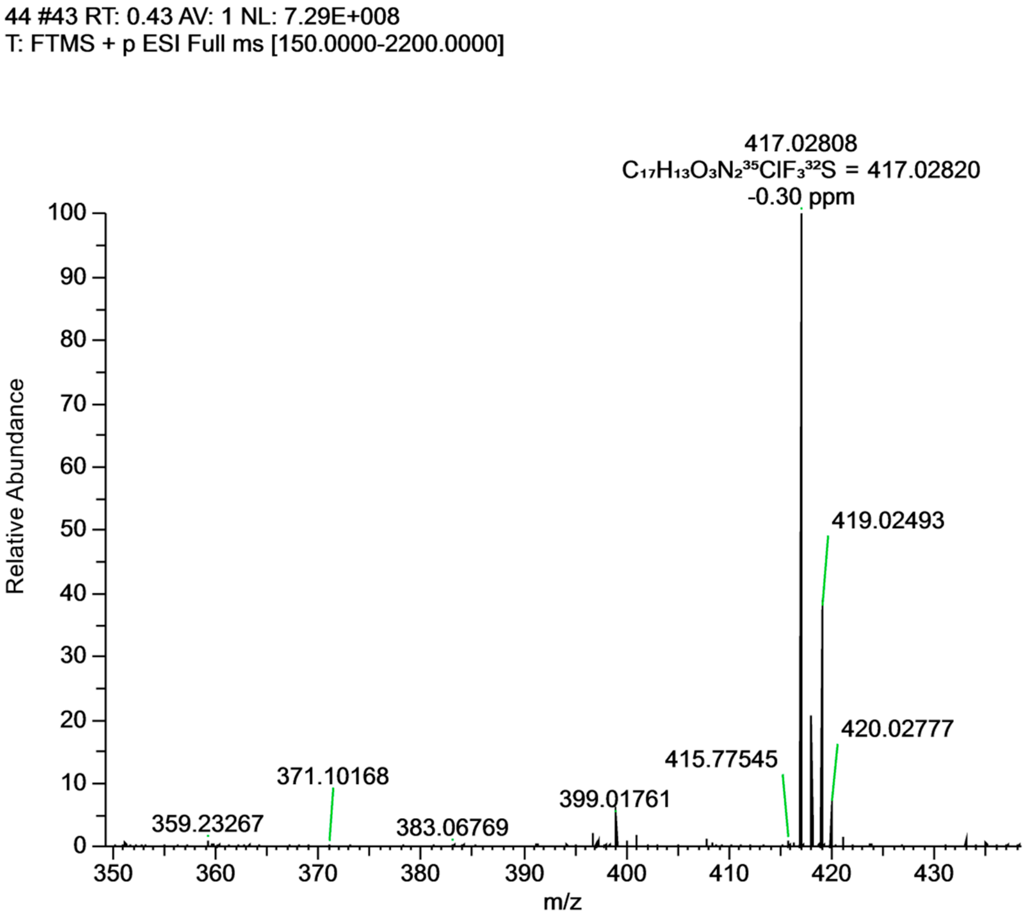

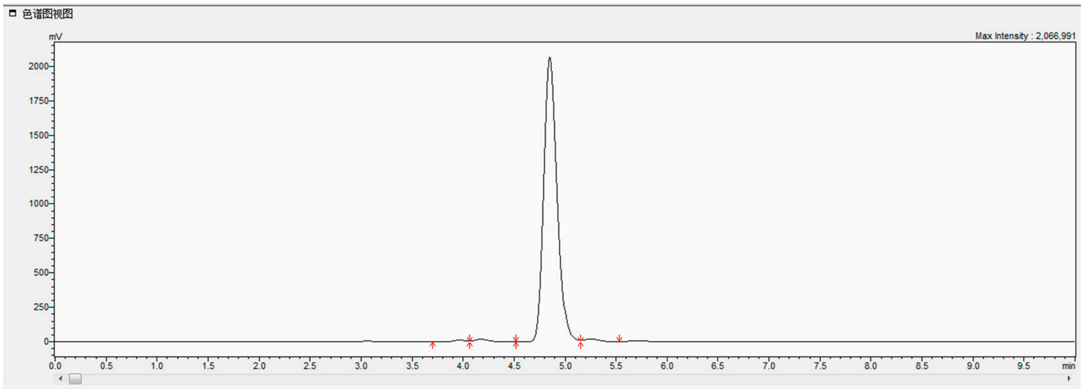

结果视图 - 峰表

峰表 | 化合物 | 组 | 校准曲线

峰号	保留时间	面积	峰宽 (高度50%)	分离度 (USP)	拖尾因	高度	面积%	理论塔板数 (USP)	拖尾因子
1	3.966	99503	0.129	--	0.000	12525	0.517	4719	--
2	4.179	171106	0.133	0.907	0.000	19529	0.889	4900	--
3	4.853	18778038	0.141	2.769	1.163	2049075	97.513	6123	1.198
4	5.255	208223	0.000	1.415	0.000	20041	1.081	4310	--
总计		19256870				2101170	100.000		

Figure S23. ^{1}H NMR, ^{13}C NMR, ^{19}F NMR, HRMS and HPLC for **E$_{23}$**.

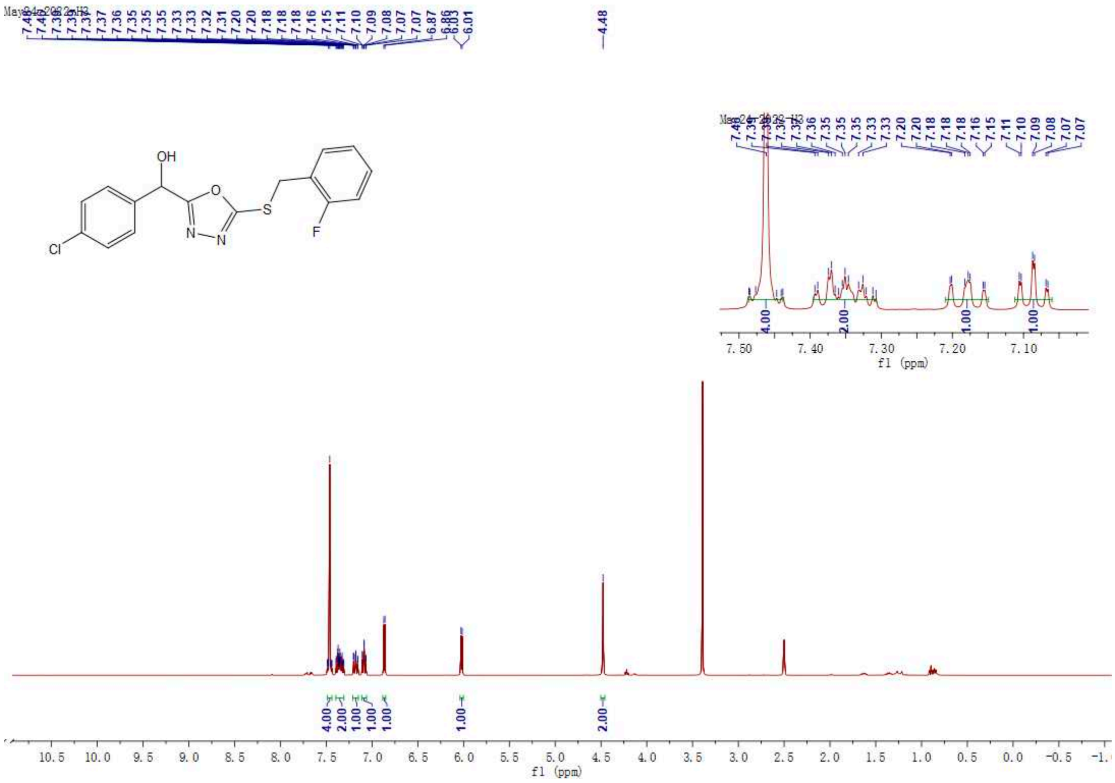
4.48
6.01
OH
O
S
N
N
Cl
F
4.00
2.00
1.00
1.00
1.00
1.00
2.00
f1 (ppm)

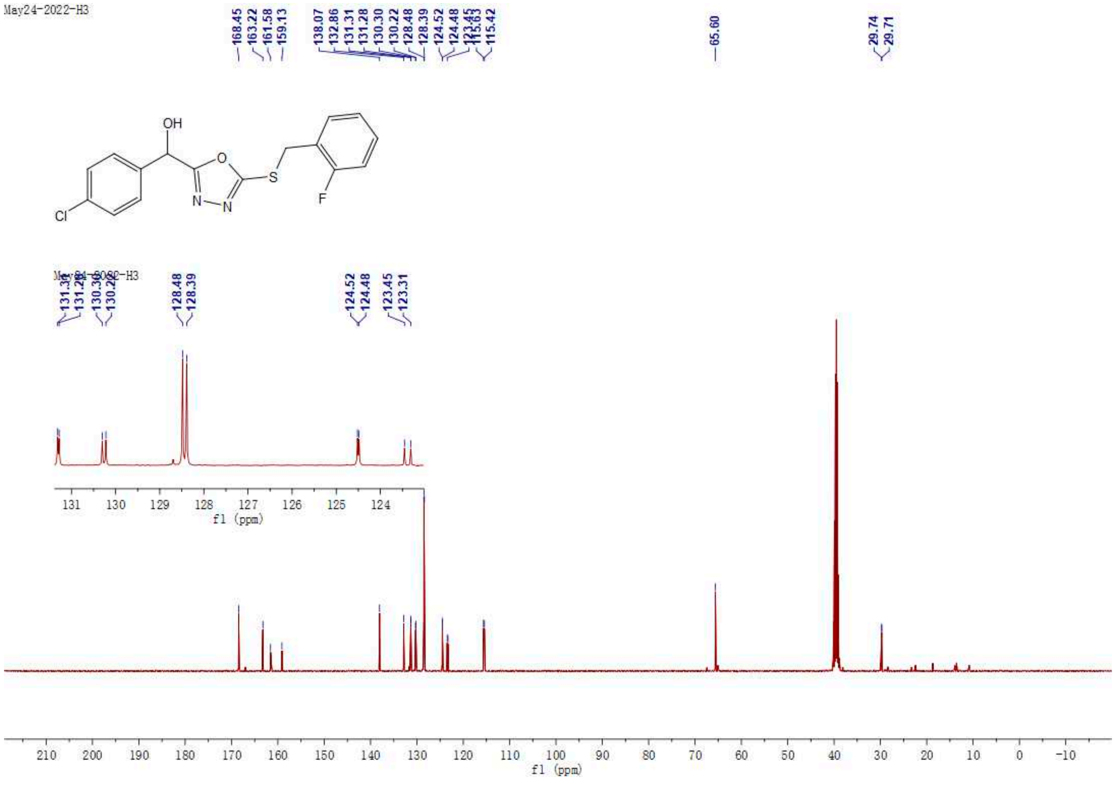
May24-2022-H3
168.45
163.22
161.58
159.13
138.07
132.86
131.31
131.28
130.30
130.22
128.48
128.39
124.52
124.48
123.45
123.31
115.63
115.42
65.60
29.74
29.71
OH
O
S
N
N
Cl
F
f1 (ppm)

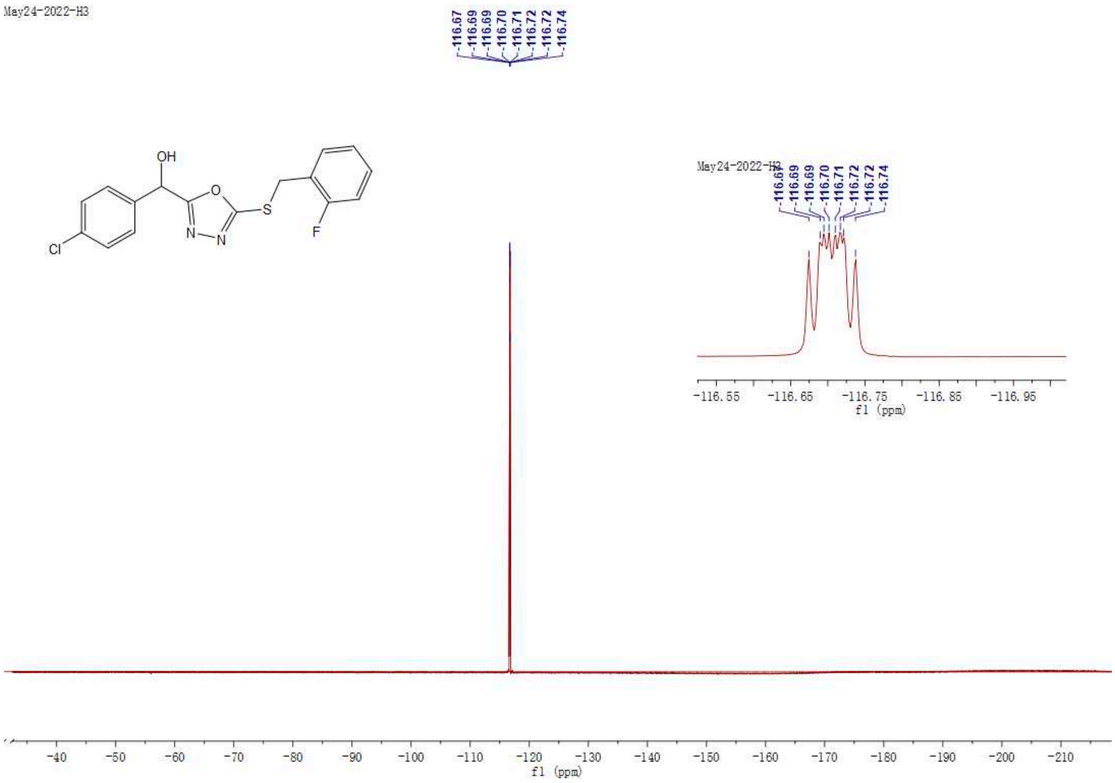

45 #31 RT: 0.32 AV: 1 NL: 9.12E+008
T: FTMS + p ESI Full ms [150.0000-2200.0000]

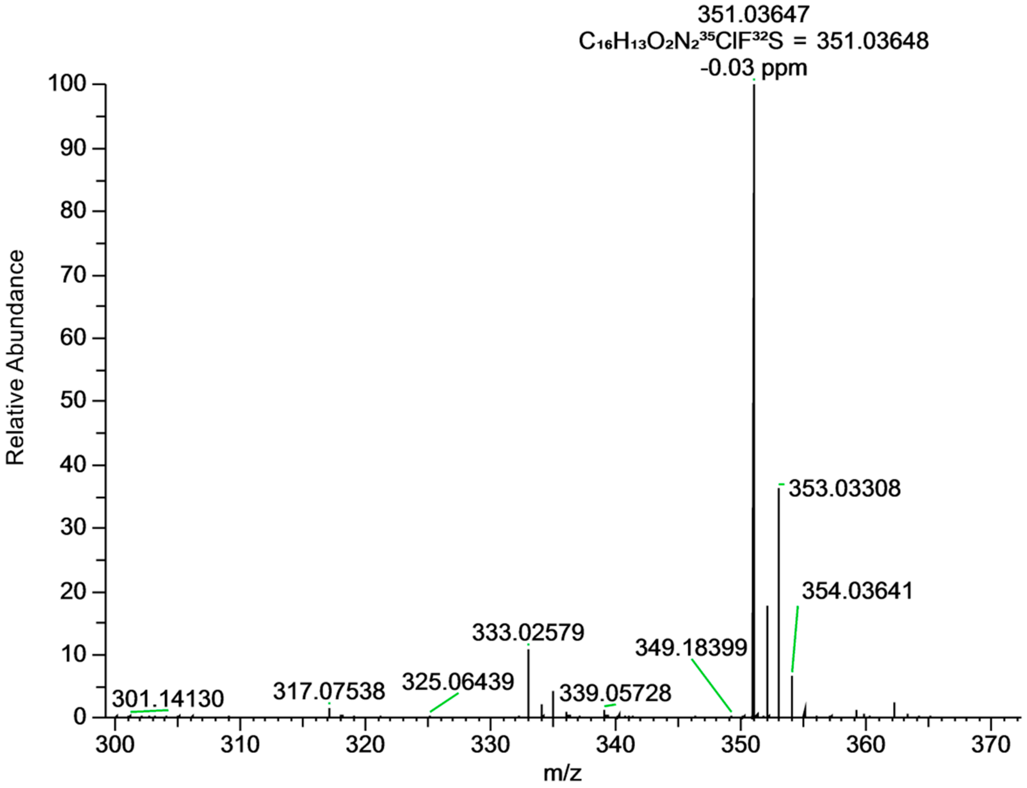

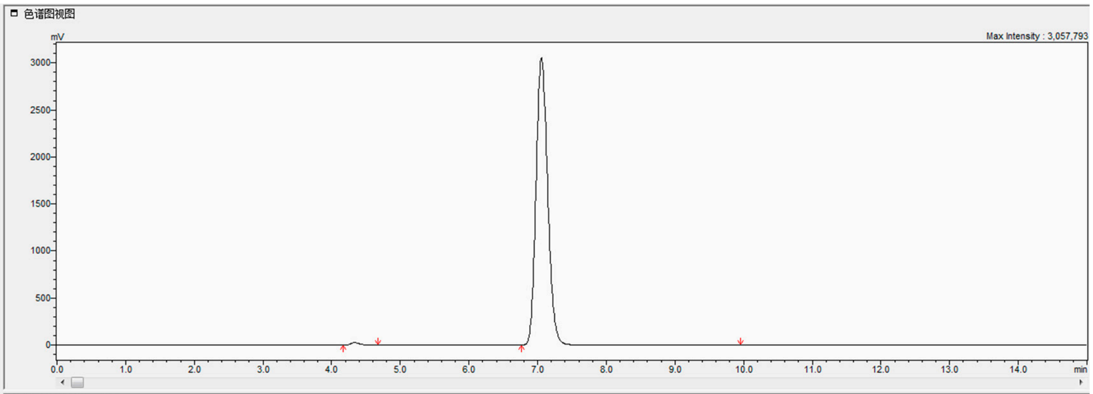

结果视图 - 峰表

峰表 | 化合物 | 组 | 校准曲线

峰号	保留时间	面积	峰宽 (高度50%)	分离度 (USP)	拖尾因	高度	面积%	理论塔板数 (USP)	拖尾因子
1	4.337	237267	0.136	--	1.198	26280	0.659	5227	1.253
2	7.057	35759822	0.180	9.848	1.116	3057774	99.341	8159	1.146
总计		35997090				3084054	100.000		

Figure S24. ^{1}H NMR, ^{13}C NMR, ^{19}F NMR, HRMS and HPLC for **E$_{24}$**.

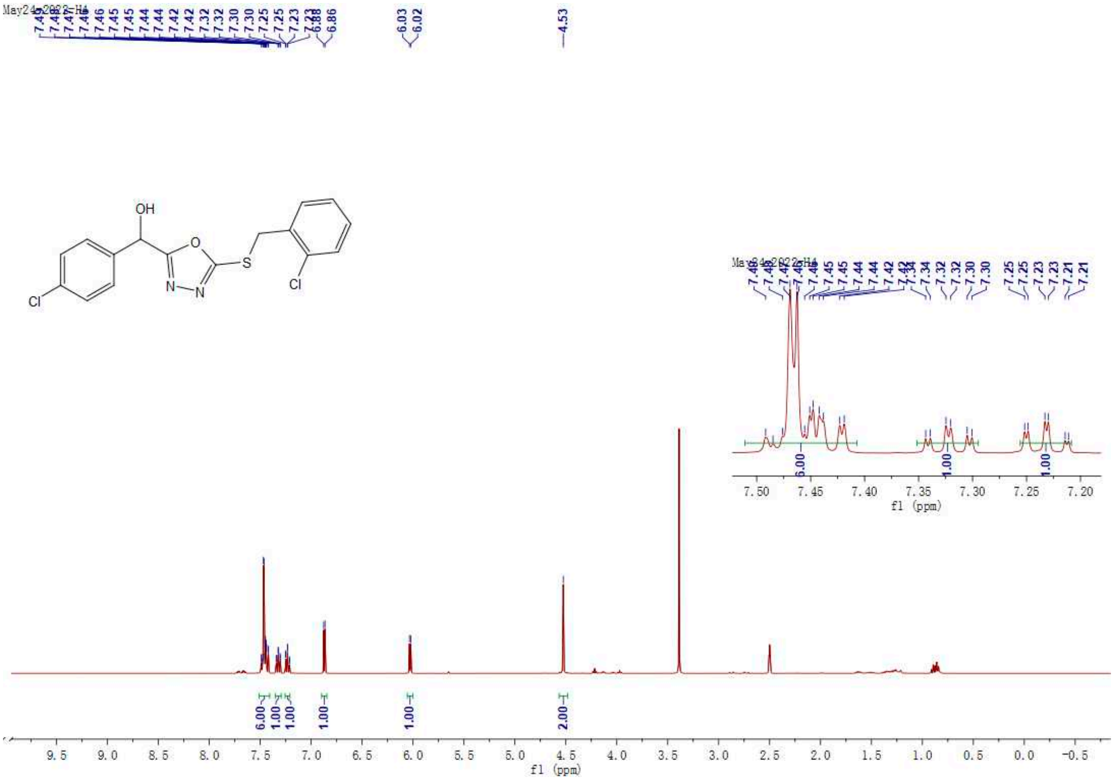

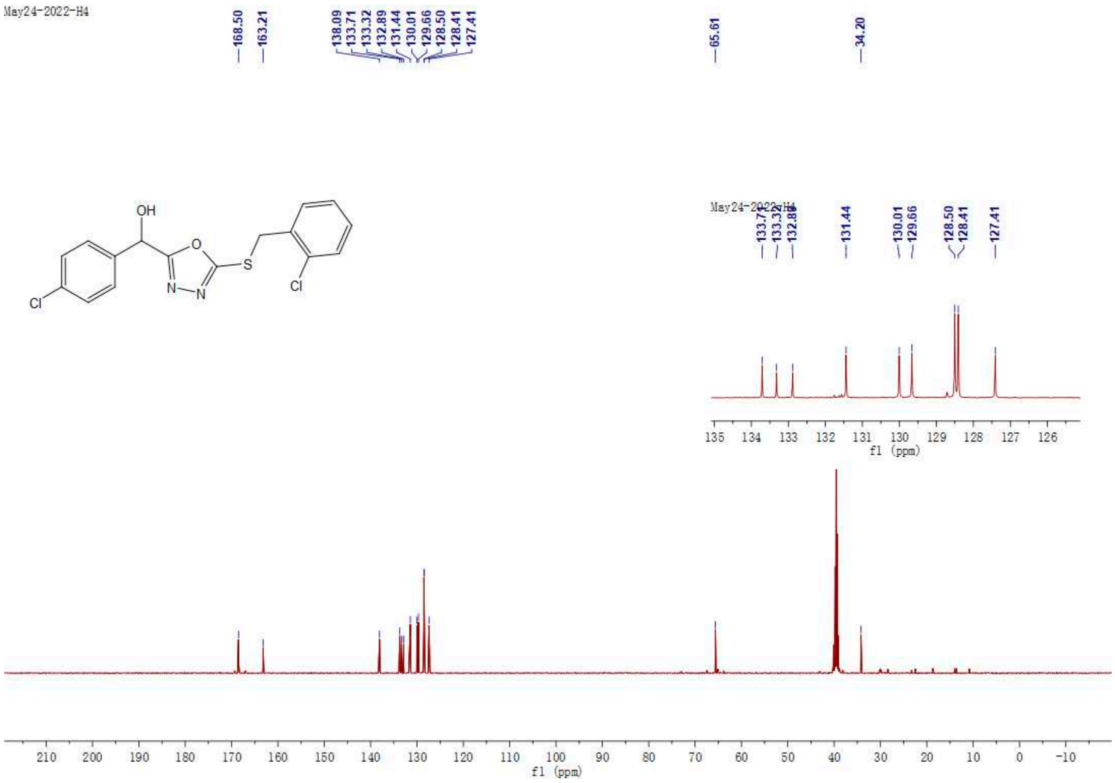
May24-2022-H4
168.50
163.21
138.09
133.71
133.32
132.89
131.44
130.01
129.66
128.50
128.41
127.41
65.61
34.20
OH
O
S
N
N
Cl
Cl
130.01
129.66
128.50
128.41
127.41
131.44
f1 (ppm)
210 200 190 180 170 160 150 140 130 120 110 100 90 80 70 60 50 40 30 20 10 0 -10

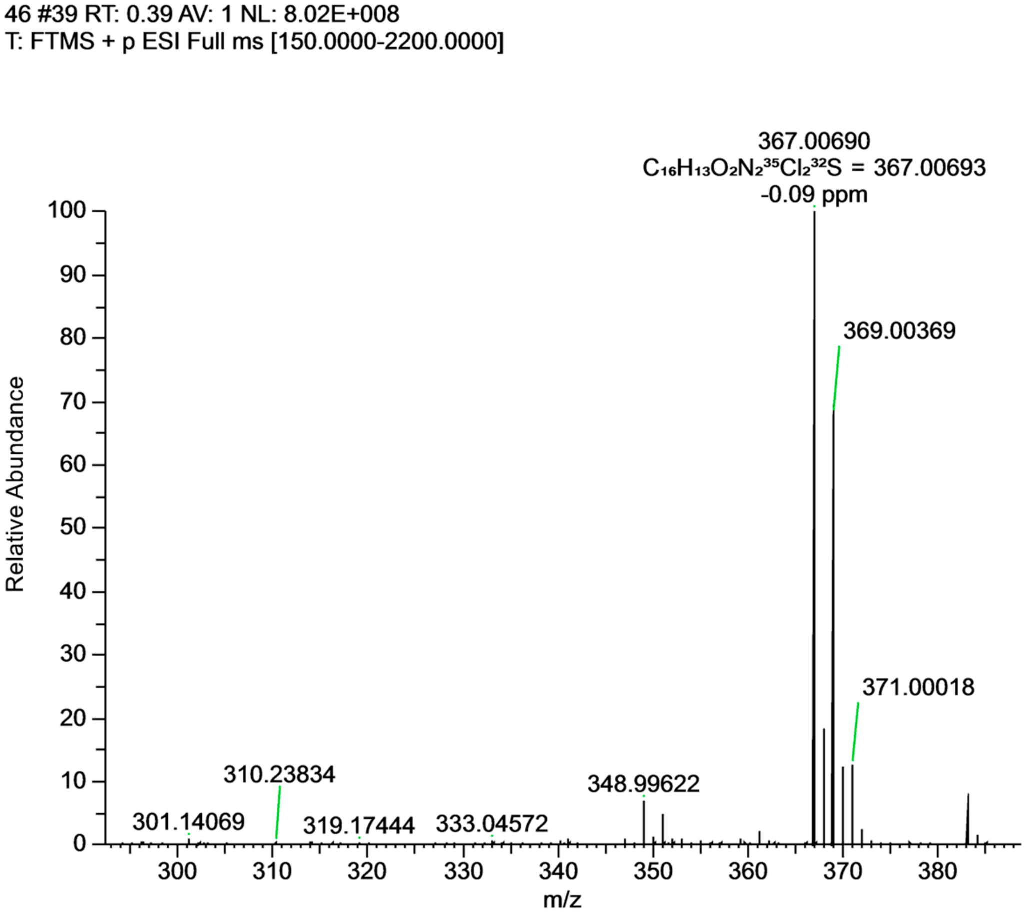
46 #39 RT: 0.39 AV: 1 NL: 8.02E+008
T: FTMS + p ESI Full ms [150.0000-2200.0000]
367.00690
$C_{16}H_{13}O_2N_2{}^{35}Cl_2{}^{32}S$ = 367.00693
-0.09 ppm
369.00369
371.00018
348.99622
310.23834
301.14069
319.17444
333.04572
Relative Abundance
m/z

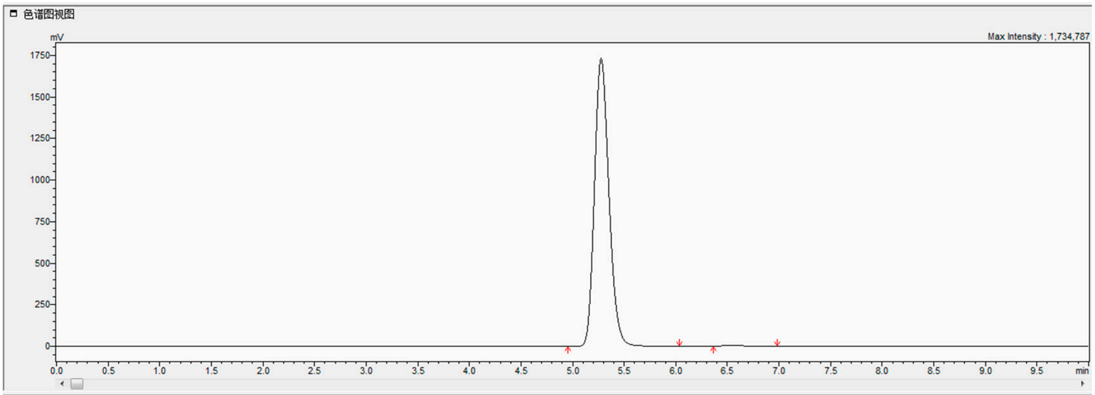

结果视图 - 峰表

峰表 | 化合物 | 组 | 校准曲线

峰号	保留时间	面积	峰宽 (高度50%)	分离度 (USP)	拖尾因	高度	面积%	理论塔板数 (USP)	拖尾因子
1	5.276	17065320	0.151	--	1.160	1734804	99.482	6286	1.192
2	6.542	88879	0.182	4.323	0.000	7360	0.518	6710	--
总计		17154198				1742164	100.000		

Figure S25. ^{1}H NMR, ^{13}C NMR, HRMS, and HPLC for $\mathbf{E_{25}}$.

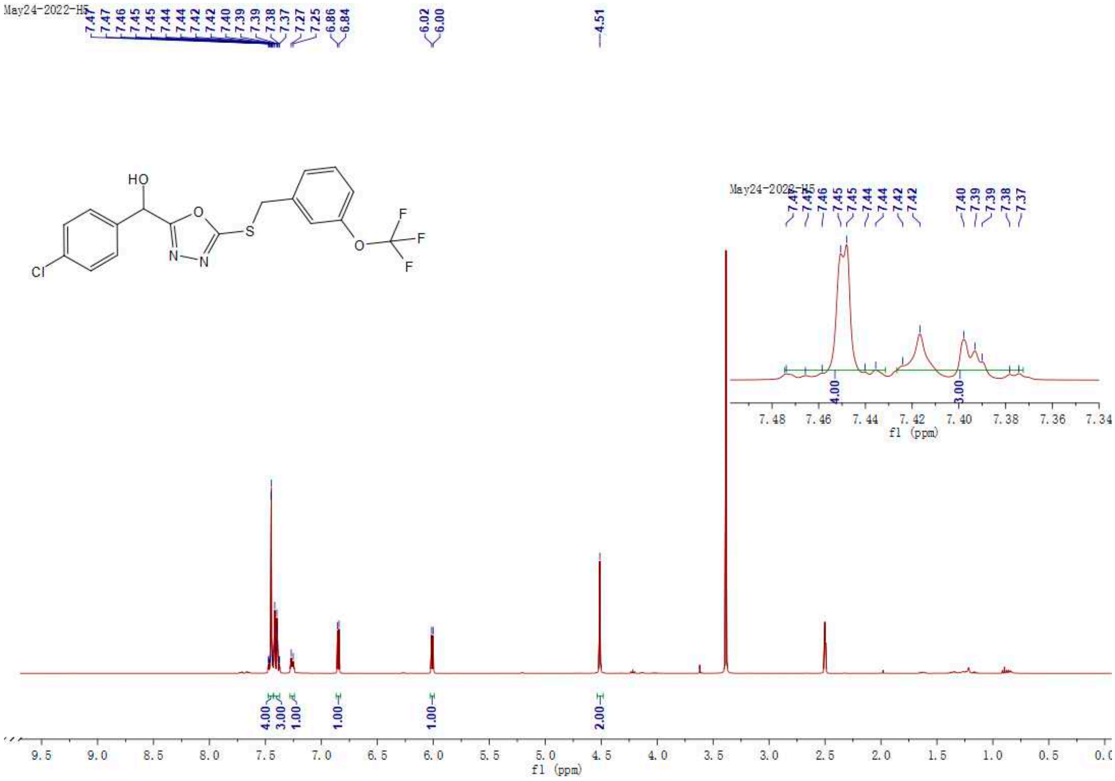

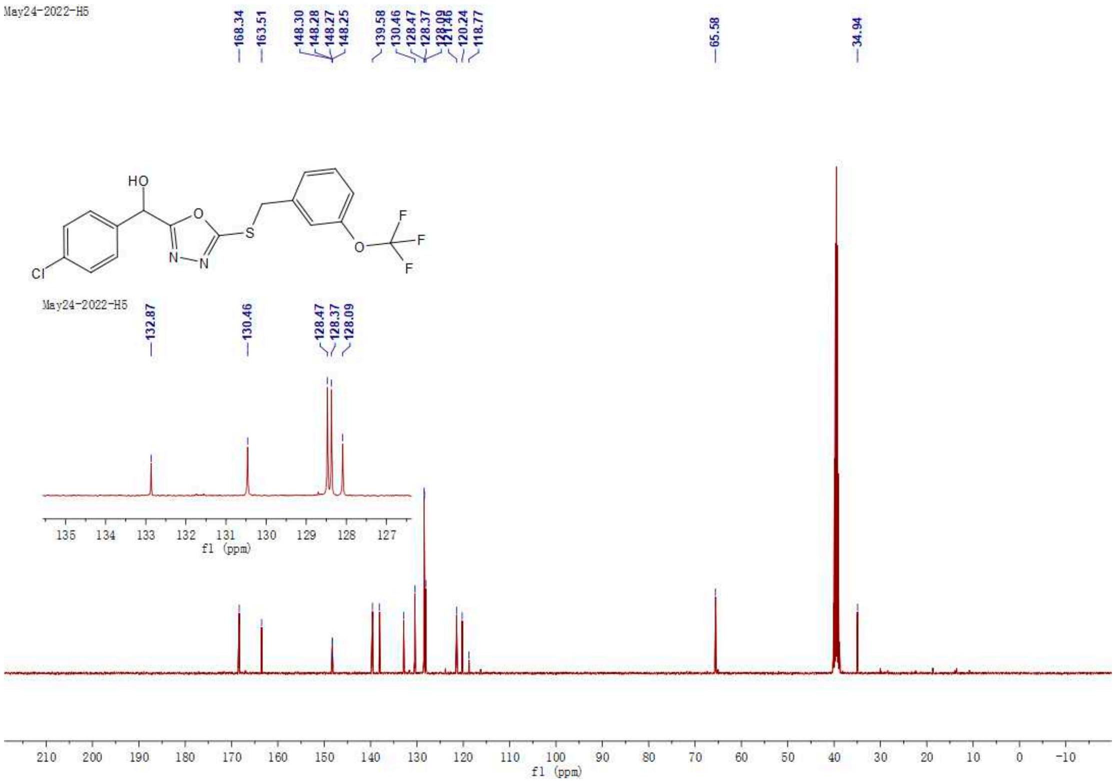
May24-2022-H5
168.34
163.51
148.30
148.28
148.27
148.25
139.58
130.46
128.47
128.37
128.09
121.46
120.24
118.77
65.58
34.94
HO
O
S
F
F
O
F
Cl
N
N
May24-2022-H5
132.87
130.46
128.47
128.37
128.09
135 134 133 132 131 130 129 128 127
f1 (ppm)
210 200 190 180 170 160 150 140 130 120 110 100 90 80 70 60 50 40 30 20 10 0 -10
f1 (ppm)

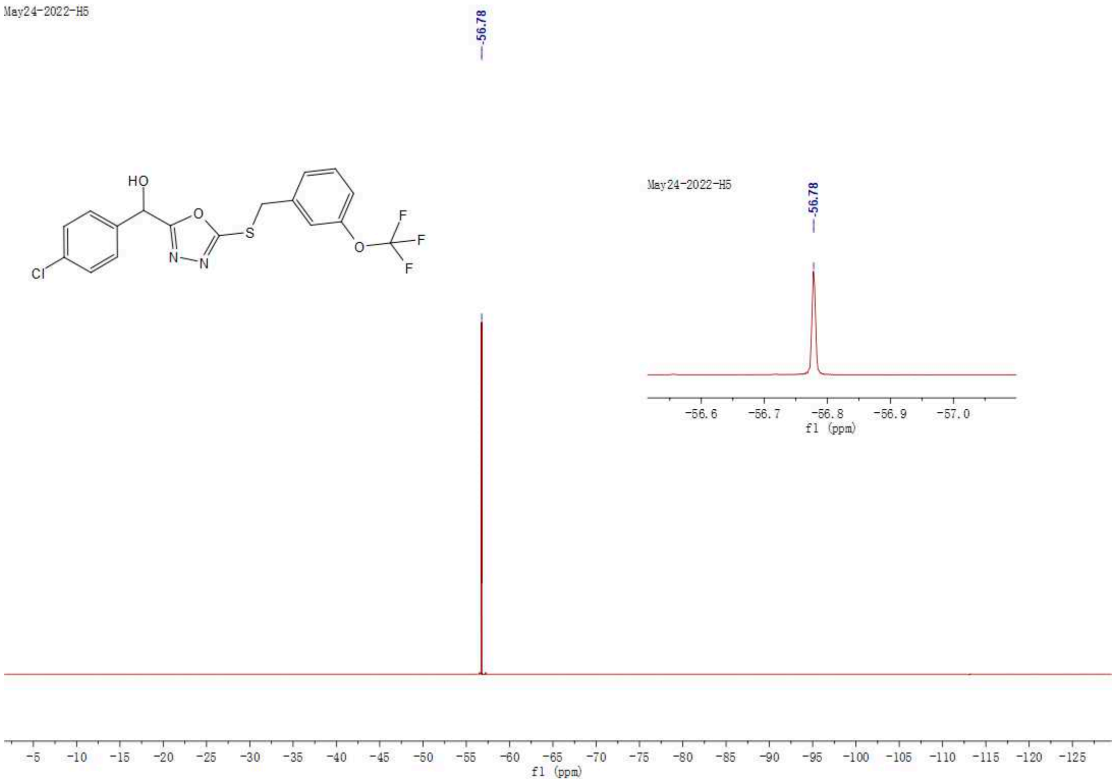
May24-2022-H5
-56.78
HO
O
S
F
F
O
F
Cl
N
N
May24-2022-H5
-56.78
-56.6 -56.7 -56.8 -56.9 -57.0
f1 (ppm)
-5 -10 -15 -20 -25 -30 -35 -40 -45 -50 -55 -60 -65 -70 -75 -80 -85 -90 -95 -100 -105 -110 -115 -120 -125
f1 (ppm)

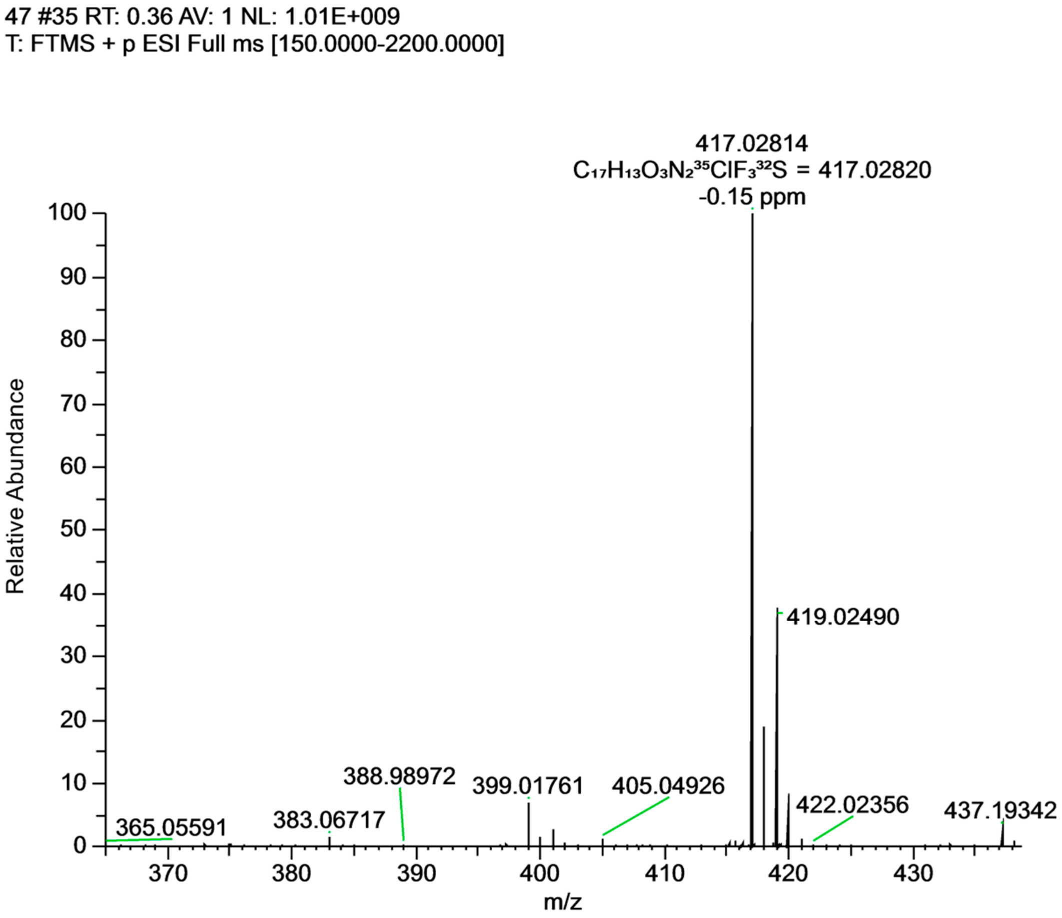

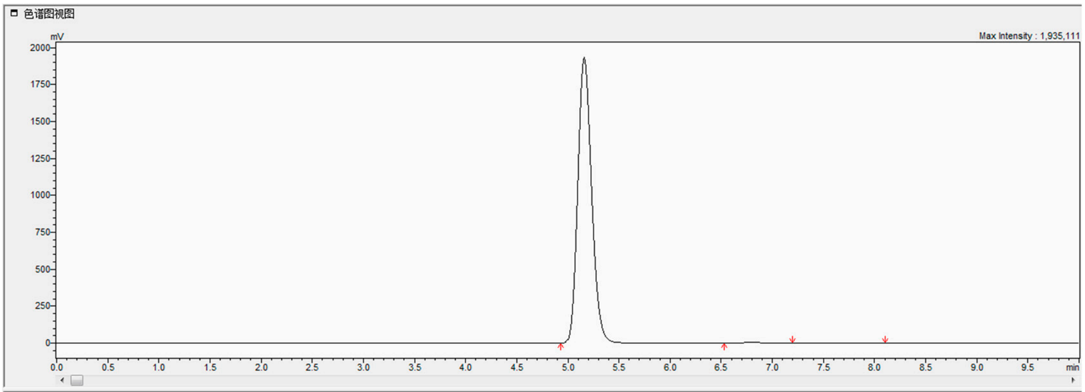

结果视图 - 峰表

峰表 | 化合物 | 组 | 校准曲线

峰号	保留时间	面积	峰宽 (高度50%)	分离度 (USP)	拖尾因	高度	面积%	理论塔板数 (USP)	拖尾因子
1	5.158	18365851	0.146	--	1.148	1935095	99.619	6402	1.180
2	6.796	70219	0.177	5.784	1.156	6076	0.381	7760	1.191
总计		18436069				1941171	100.000		

Figure S26. ^{1}H NMR, ^{13}C NMR, ^{19}F NMR, HRMS and HPLC for **E$_{26}$**.

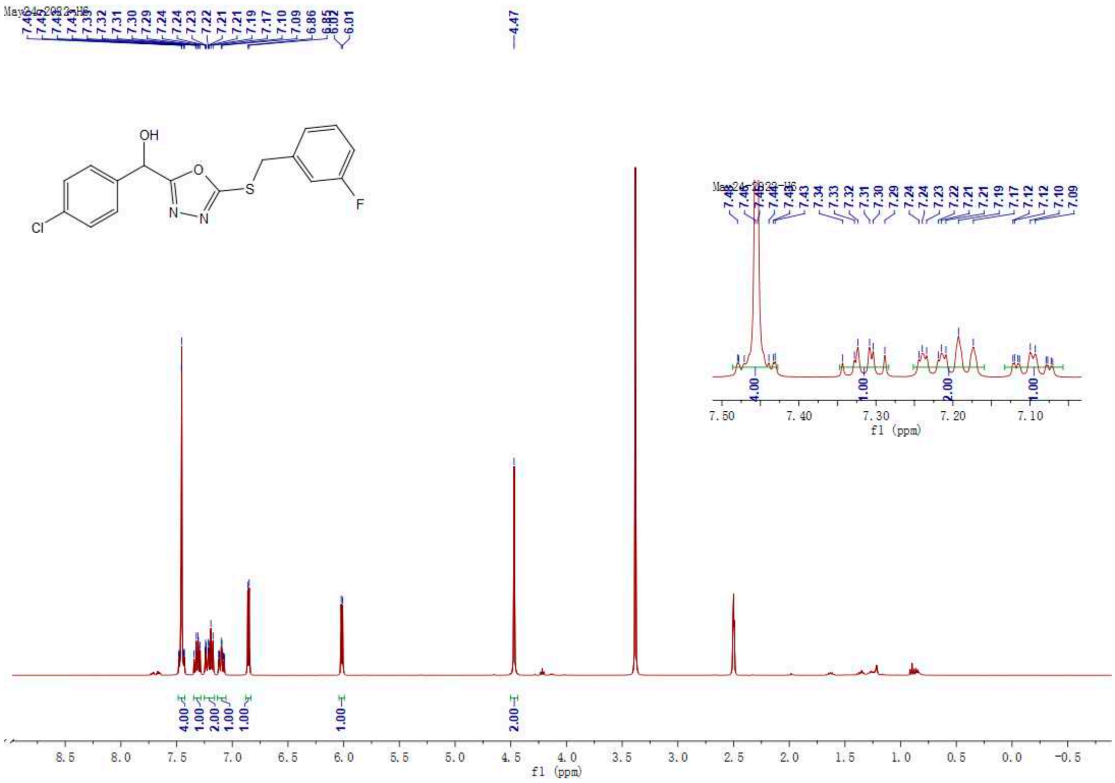
May24-2022-H6
4.47
OH
O
S
F
N
N
Cl
4.00
1.00
2.00
1.00
1.00
1.00
2.00
f1 (ppm)

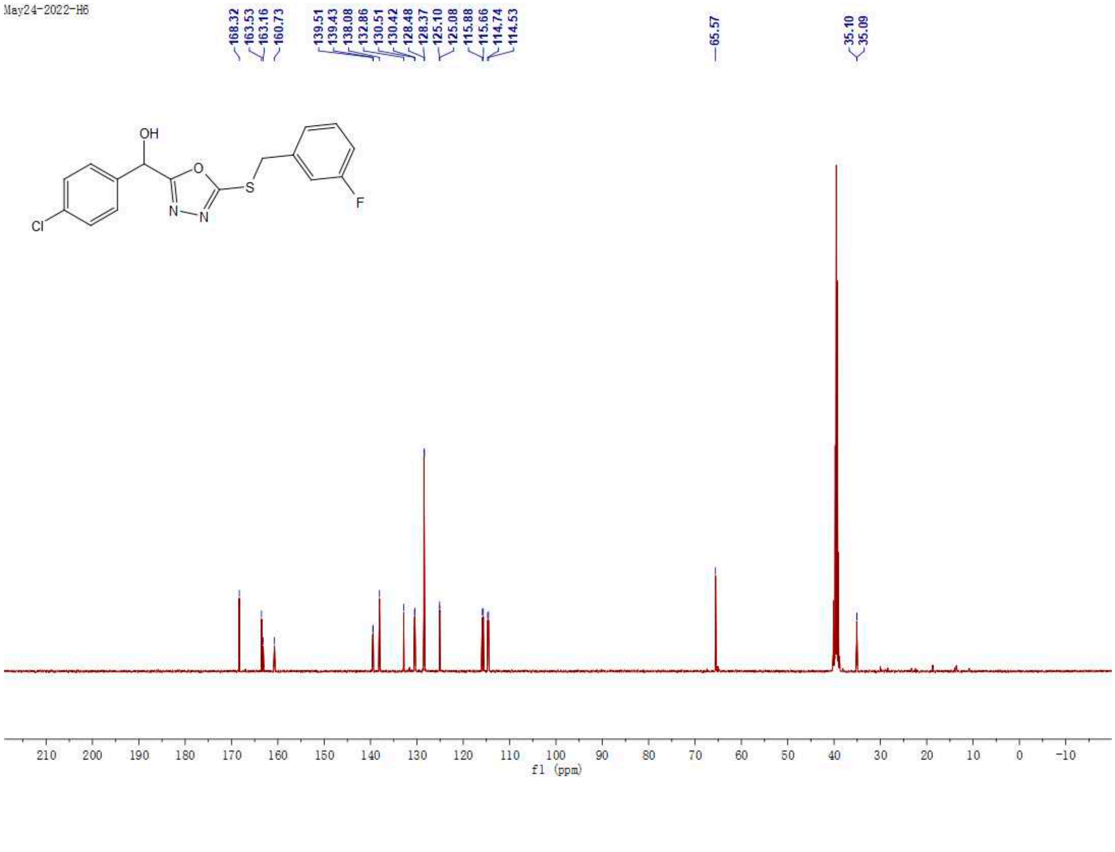
May24-2022-H6
168.32
163.53
163.16
160.73
139.51
139.43
138.08
132.86
130.51
130.42
128.48
128.37
125.10
125.08
115.88
115.66
114.74
114.53
65.57
35.10
35.09
OH
O
S
F
N
N
Cl
f1 (ppm)

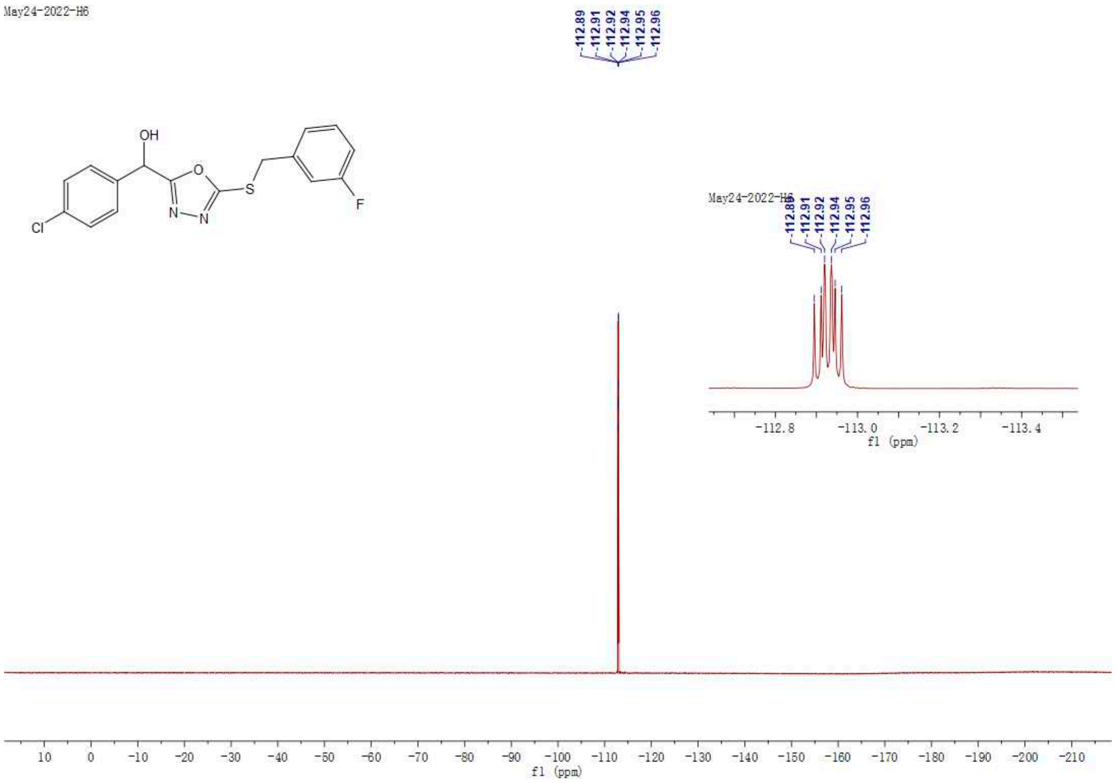

48 #33 RT: 0.34 AV: 1 NL: 7.13E+008
T: FTMS + p ESI Full ms [150.0000-2200.0000]

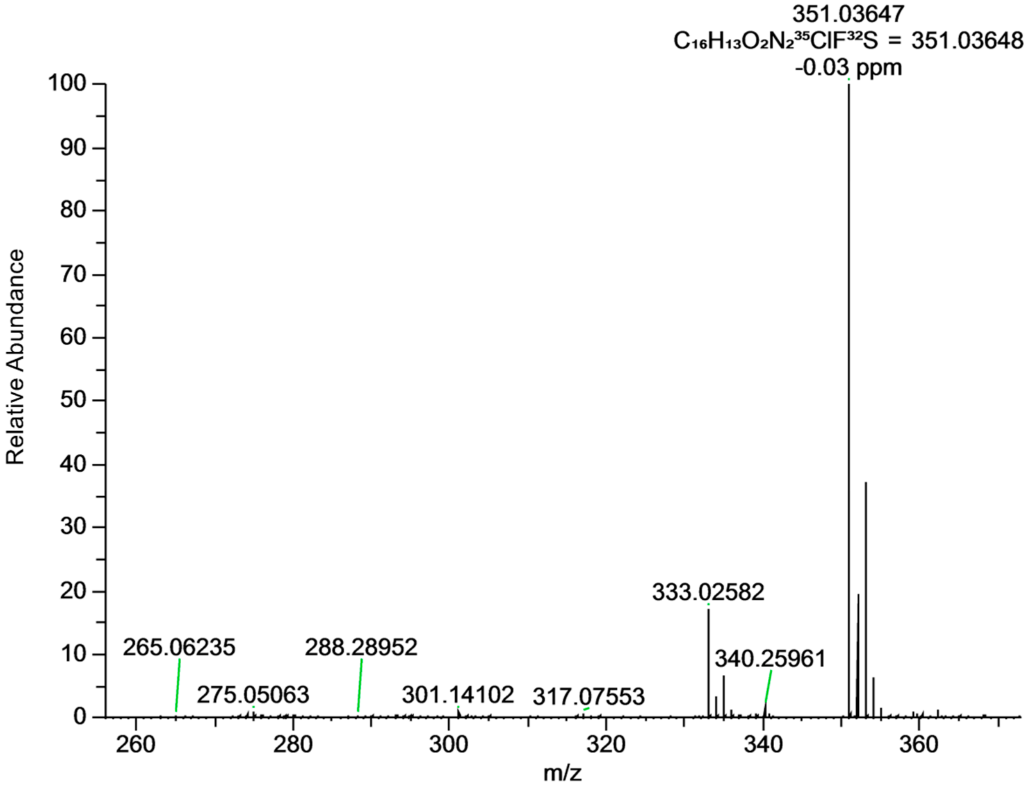

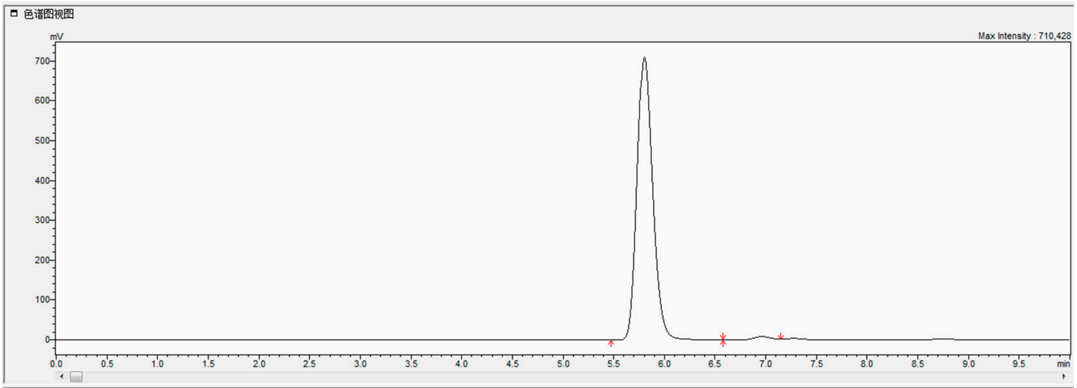

峰号	保留时间	面积	峰宽 (高度50%)	分离度 (USP)	拖尾因	高度	面积%	理论塔板数 (USP)	拖尾因子
1	5.800	7486398	0.162	--	1.158	710431	98.678	6684	1.192
2	6.963	100292	0.187	3.810	0.000	8223	1.322	7270	--
总计		7586690				718654	100.000		

Figure S27. ^{1}H NMR, ^{13}C NMR, ^{19}F NMR, HRMS and HPLC for **E_{27}**.

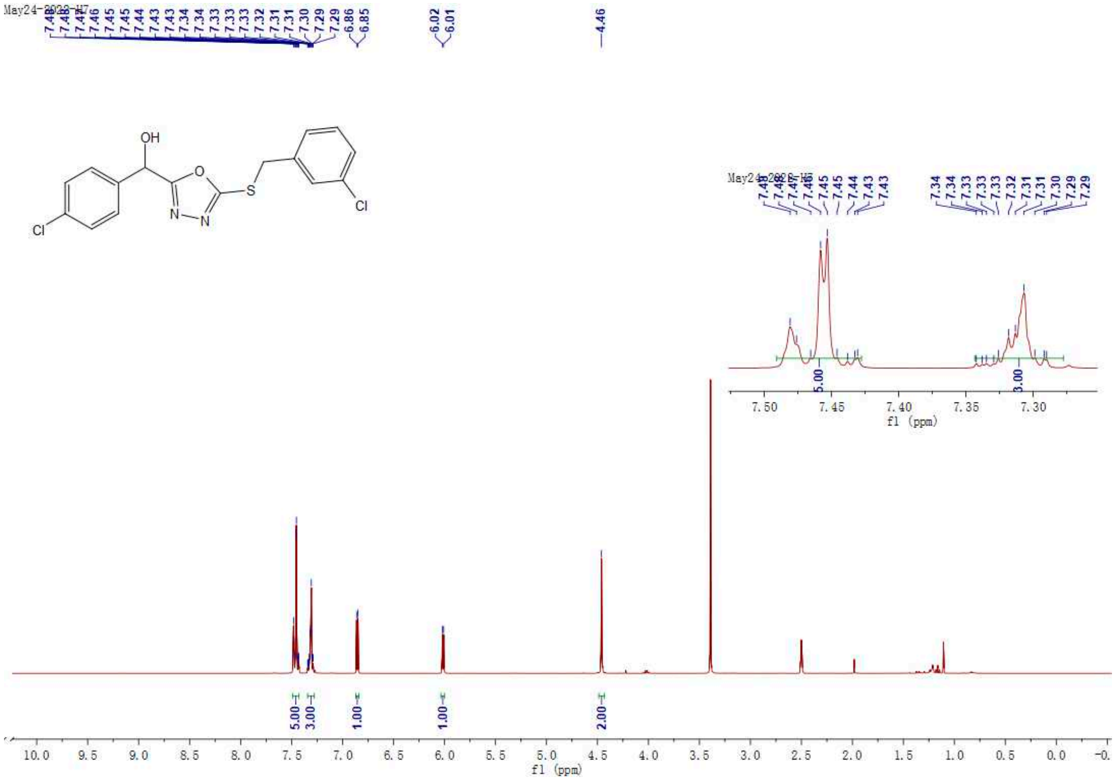

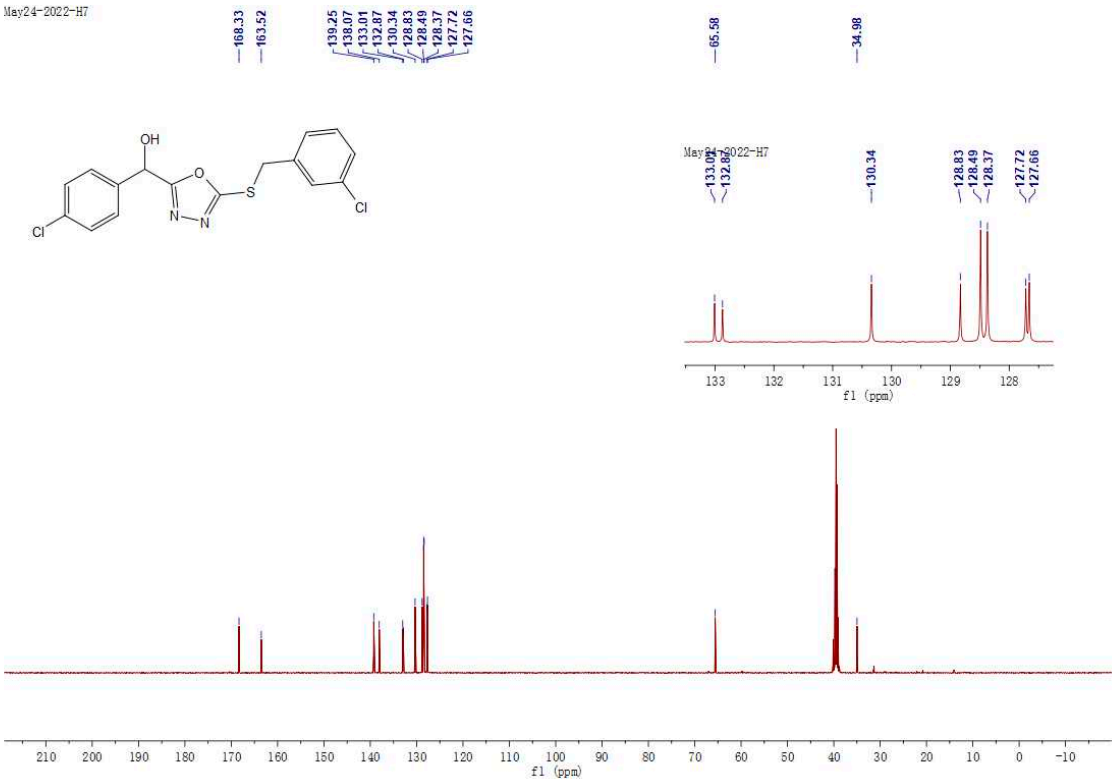

49 #39 RT: 0.39 AV: 1 NL: 4.39E+008
T: FTMS + p ESI Full ms [150.0000-2200.0000]

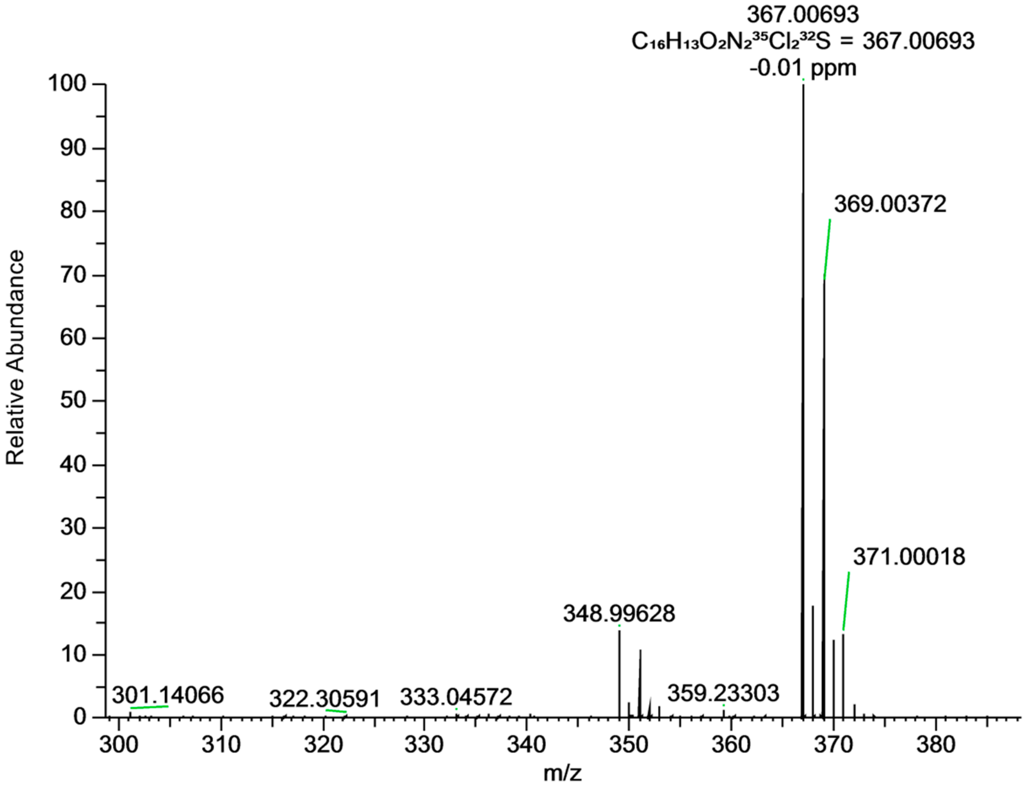

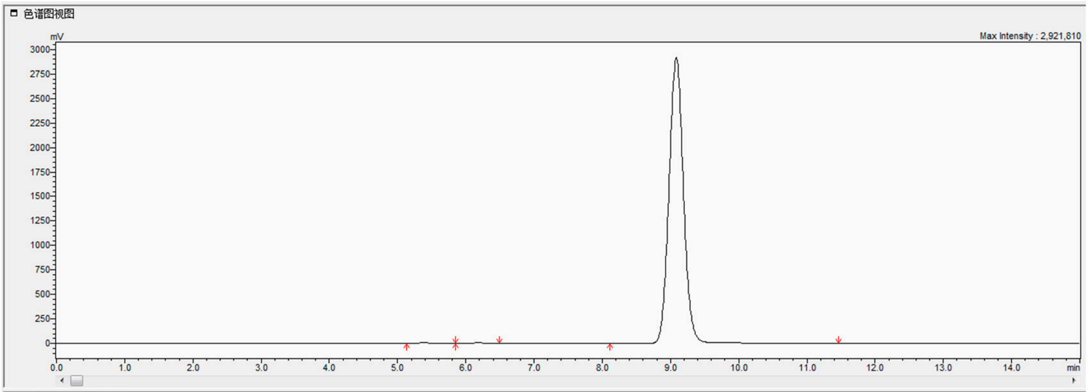

结果视图 - 峰表

峰表 | 化合物 | 组 | 校准曲线

峰号	保留时间	面积	峰宽 (高度50%)	分离度 (USP)	拖尾因	高度	面积%	理论塔板数 (USP)	拖尾因子
1	5.377	86867	0.167	--	1.228	7735	0.202	5342	1.254
2	6.179	77862	0.169	2.712	1.062	6735	0.181	6925	0.969
3	9.085	42875880	0.223	8.485	1.041	2921702	99.617	8768	1.067
总计		43040610				2936173	100.000		

Figure S28. ^{1}H NMR, ^{13}C NMR, HRMS, and HPLC for **E_{28}**.

3. Crystallographic data of compound E_9

Table S1

Crystallographic data of compound **D_9**

Chemical formula	$C_{17}H_{15}FN_2O_2S$
Formula weight	330.37
Temperature[K]	273 K
Crystal system	Orthorhombic
Space group	P c a 21
a [Å]	11.2260 (5)
b [Å]	17.9952 (8)
c [Å]	7.8108 (3)
α [°]	90
β [°]	90
γ [°]	90
V[Å^3]	1577.89 (12)
Z	4
ρ (calculated)[g/cm^3]	1.391 g cm^{-1}
μ [mm^{-1}]	2.018 mm^{-1}
F (000)	688.0
Crystal size [mm^3]	0.22 × 0.21 × 0.18
Color, shape	Water clear crystal, Peism
Radiation [Å]	MoKα (λ = 1.54178)
Theta Min-Max [°]	4.642, 72.080
h, k, l	--13 ≤ h ≤ 12, -17 ≤ k ≤ 22, -7 ≤ l ≤ 9
Reflections collected	2728
Independent reflections	2645
Data/restraints/parameters	3843/4/243
Goodness-of-fit	1.076
Final R indexes [I>=2σ (I)]	R1= 0.0435, wR2= 0.1112
Final R indexes [all data]	R1= 0.0443, wR2= 0.1112

4. The regression equation of the title compounds with EC_{50} values

Table S2

The regression equation of the title compounds with EC_{50} values against *G. saubinetii* in vitro. [a]

Compound	*G. saubinetii*		
	EC_{50} (mg L^{-1})	Regression equation	R^2
**E_1**	47.4±2.6	y = 1.6838x + 2.1778	0.977
**E_2**	37.3±1.4	y = 1.4566x + 2.7104	0.946
**E_6**	49.9±0.4	y = 1.1125x + 3.1111	0.939
**E_7**	30.6±3.0	y = 1.6075x + 2.6106	0.975
**E_8**	79.1±0.6	y = 1.2835x + 2.564	0.980
**E_9**	24.6±0.7	y = 1.1498x + 3.4001	0.931
**E_{10}**	29.4±0.6	y = 1.5341x + 2.7478	0.993
**E_{13}**	20.4±1.3	y = 1.3199x + 3.271	0.967
**E_{14}**	21.5±1.0	y = 1.3439x + 3.2081	0.974
**E_{17}**	22.0±1.5	y = 1.0217x + 3.6276	0.905
**E_{18}**	25.4±0.9	y = 1.1308x + 3.4115	0.990
**E_{19}**	56.1±0.9	y = 1.1576x + 2.8856	0.987
**E_{20}**	31.6±0.6	y = 1.3543x + 2.9688	0.972
**E_{21}**	27.3±2.0	y = 0.9419x + 3.6478	0.9241
**E_{22}**	51.1±0.2	y=1.8303x+2.1571	0.974
**E_{23}**	32.5±0.9	y=1.5487x+2.6594	0.941
**E_{24}**	30.8±1.5	y=1.4254x+2.8775	0.991
**E_{25}**	31.3±1.5	y=1.3693x+2.9521	0.975

**E_{26}**	32.7±1.1	y=1.5711x+2.6199	0.930
**E_{27}**	25.2±1.0	y=1.2166x+3.2957	0.984
**E_{28}**	24.2±0.4	y=1.2832x+3.2237	0.972

[a] Values are means ± SD of three replicates.

Table S3

The regression equation of the title compounds with EC_{50} values against *V. dahliae* in vitro. [a]

Compound	*V. dahliae*		
	EC_{50} (mg L^{-1})	Regression equation	R^2
**E_1**	50.7±2.8	y = 2.0379x + 1.5258	0.997
**E_2**	37.2±1.2	y = 1.7372x + 2.272	0.933
**E_6**	12.7±0.5	y = 1.5729x + 3.2621	0.970
**E_7**	14.3±0.1	y = 1.0199x + 3.8215	0.999
**E_8**	＞100	y = 1.1396x + 2.5042	0.979
**E_9**	29.0±2.1	y = 1.5996x + 2.6596	0.984
**E_{10}**	37.2±1.0	y = 2.9854x + 0.3104	0.969
**E_{13}**	18.5±1.2	y = 3.3564x + 0.7478	0.901
**E_{14}**	23.1±0.8	y = 2.4461x + 1.6658	0.984
**E_{17}**	16.1±0.2	y = 1.382x + 3.332	0.974
**E_{18}**	15.8±1.4	y = 1.2028x + 3.5587	0.902
**E_{19}**	65.7±0.7	y = 1.4397x + 2.3835	0.948
**E_{20}**	29.6±2.3	y = 2.6387x + 1.1189	0.951
**E_{21}**	13.4±0.6	y = 1.1821x + 3.6673	0.960
**E_{22}**	61.3±1.6	y=1.6208x+ 2.1025	0.993

**E_{23}**	32.3±0.4	y=1.7528x+2.4201	0.997
**E_{24}**	38.5±1.0	y=2.2253x+1.4731	0.996
**E_{25}**	33.7±3.5	y=1.5801x+2.5869	0.974
**E_{26}**	30.4±1.3	y=1.9004x+2.1816	0.958
**E_{27}**	43.0±1.6	y=1.6554x+ 2.2961	0.970
**E_{28}**	26.9±0.2	y=1.4439x+ 2.9346	0.917

[a] Values are means ± SD of three replicates.

Table S4

The regression equation of the title compounds with EC_{50} values against *S. sclerotiorum* in vitro. [a]

Compound	*S. sclerotiorum*		
	EC_{50} (mg L^{-1})	Regression equation	R^2
**E_1**	23.0±1.5	y = 0.7946x + 4.6336	0.962
**E_2**	27.1±1.5	y = 1.0694x + 3.4795	0.947
**E_6**	90.5±3.5	y = 1.0506x + 2.9442	0.976
**E_7**	40.1±2.7	y = 1.4621x + 2.6562	0.957
**E_8**	13.8±3.1	y = 1.1276x + 3.7162	0.976
**E_9**	10.3±1.0	y = 1.6198x + 3.3315	0.951
**E_{10}**	21.9±0.4	y = 1.5274x + 2.9524	0.903
**E_{13}**	33.5±1.8	y = 2.8357x + 0.6741	0.965
**E_{14}**	37.6±1.9	y = 1.4025x + 2.7904	0.990
**E_{17}**	27.9±0.2	y = 2.137x + 1.9092	0.980
**E_{18}**	8.0±0.3	y = 0.7293x + 4.3422	0.956
**E_{19}**	47.8±1.7	y = 1.6349x + 2.2543	0.998

**E_{20}**	39.1±1.1	y = 1.779x + 2.1667	0.919
**E_{21}**	36.3±0.3	y = 1.6215x + 2.4701	0.988
**E_{22}**	48.5±0.4	y=1.6458x+ 2.2258	0.981
**E_{23}**	81.4±2.1	y=1.4601x+ 2.2101	0.948
**E_{24}**	51.6±5.4	y=1.3459x+2.6952	0.997
**E_{25}**	75.2±3.4	y=1.5022x+ 2.1818	0.958
**E_{26}**	54.0±1.8	y=1.4843x+2.4292	0.986
**E_{27}**	37.2±0.8	y=1.8064x+2.1623	0.968
**E_{28}**	47.9±0.2	y=1.6462x+2.2342	0.984

[a] Values are means ± SD of three replicates.

Table S5

The regression equation of the title compounds with EC_{50} values against *T. cucumeris* in vitro. [a]

Compound	*T. cucumeris*		
	EC_{50} (mg L^{-1})	Regression equation	R^2
**E_5**	18.5±0.4	y = 1.6449x + 2.9151	0.943
**E_7**	5.7±0.2	y = 0.8461x + 4.3567	0.968
**E_{10}**	36.3±1.3	y = 1.6703x + 2.3936	0.955
**E_{13}**	7.1±0.1	y = 1.1273x + 4.0374	0.953
**E_{21}**	37.6±1.0	y = 1.0234x + 3.3884	0.925
hymexazol	13.8±1.5	y = 1.0312x + 3.825	0.999

[a] Values are means ± SD of three replicates.

Table S6

The regression equation of the title compounds with EC_{50} values against *P. capsici* in vitro. [a]

Compound	*P. capsici*		
	EC_{50} (mg L^{-1})	Regression equation	R^2
**E_{13}**	42.9±0.7	y = 1.95x + 1.8161	0.988
**E_{14}**	49.3±1.4	y = 1.6695x + 2.1737	0.971
**E_{20}**	50.9±1.4	y = 1.709x + 2.0825	0.965
hymexazol	17.9±1.3	y = 0.7519x + 4.0573	0.916

[a] Values are means ± SD of three replicates.
